# Study on Design, Synthesis and Herbicidal Activity of Novel 6-Indazolyl-2-picolinic Acids

**DOI:** 10.3390/molecules29020332

**Published:** 2024-01-09

**Authors:** Qing Liu, Rong-Chuan Shi, Hui-Ting Li, Wei Wei, Xiao Yuan, Shang-Zhong Liu, Yi-Ming Cao

**Affiliations:** 1 Innovation Center of Pesticide Research, Department of Applied Chemistry, College of Science, China Agricultural University, Beijing 100193, China; qingqing@cau.edu.cn (Q.L.); shirongchuan@cau.edu.cn (R.-C.S.); bs20223100772@cau.edu.cn (H.-T.L.); s20213101991@cau.edu.cn (W.W.); sy20223102171@cau.edu.cn (X.Y.); shangzho@cau.edu.cn (S.-Z.L.); 2 Key Laboratory of National Forestry and Grassland Administration on Pest Chemical Control, China Agricultural University, Beijing 100193, China

**Keywords:** 2-picolinic acid, scaffold hopping, herbicidal activity, mode of action, auxin genes

## Abstract

Thirty-eight new 4-amino-3,5-dicholo-6-(1*H*-indazolyl)-2-picolinic acids and 4-amino-3,5-dicholo-6-(2*H*-indazolyl)-2-picolinic acids were designed by scaffold hopping and synthesized to discover potential herbicidal molecules. All the new compounds were tested to determine their inhibitory activities against *Arabidopsis thaliana* and the root growth of five weeds. In general, the synthesized compounds exhibited excellent inhibition properties and showed good inhibitory effects on weed root growth. In particular, compound **5a** showed significantly greater root inhibitory activity than picloram in *Brassica napus* and *Abutilon theophrasti* Medicus at the concentration of 10 µM. The majority of compounds exhibited a 100% post-emergence herbicidal effect at 250 g/ha against *Amaranthus retroflexus* and *Chenopodium album*. We also found that 6-indazolyl-2-picolinic acids could induce the up-regulation of auxin genes ACS7 and NCED3, while auxin influx, efflux and auxin response factor were down-regulated, indicating that 6-indazolyl-2-picolinic acids promoted ethylene release and ABA production to cause plant death in a short period, which is different in mode from other picolinic acids.

## 1. Introduction

The production loss caused by diseases, pests, and weeds in different crops and regions ranges from 30% to more than 90% [1]. At present, about 250 plant species classified as weeds in arable land influence the normal growth of crops through competing limited land, water, and other nutrients. Application of synthetic herbicides is the most widely used and effective method to control weeds [2], and has been used for more than 70 years. Currently, herbicides account for more than 40% of the global pesticide market; however, weed damage still results in 8–13% global crop losses every year [3,4]. Furthermore, the long-term and extensive application of herbicides has triggered the exponential increase in some herbicide-resistant weeds, seriously threatening the productivity and profitability of the farms or companies. Until now, at least 60 countries have reported herbicide-resistant weed biotypes, including more than 500 herbicide combinations, and the annual cost of treating herbicide-resistant weeds is about USD 4 billion worldwide [5,6,7,8].

**Figure 1:**
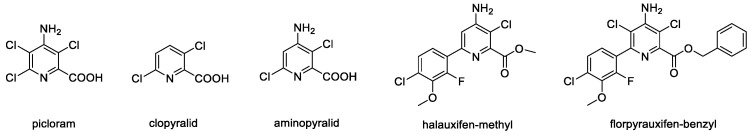
Commercial 2-picolinic acid and 2-picolinate herbicides from 1940s.

Synthetic auxinic herbicides mimic the natural plant hormone indole-3-acetic acid (IAA) and some have already been used for decades, but the weeds generate corresponding resistance against them much slower than others, which can be attributed to the potential multiple sites of actions and complex action mechanism of this kind of herbicides [6,7,9,10]. Synthetic auxinic herbicides contain several structural skeletons, in particular 2-picolinic acid. During the 1940s, Corteva (former Dow AgroSciences) discovered a series of herbicides containing structural skeleton 2-picolinic acid, such as picloram, clopyralid, aminopyralid, halauxifen-methyl (Arylex^TM^ active) and florpyrauxifen-benzyl (Rinskor^TM^ active). In recent years, the last two were launched following intensive research on the structure–activity relationship and soil metabolites (Figure 1) [11,12,13,14].

**Figure 2:**
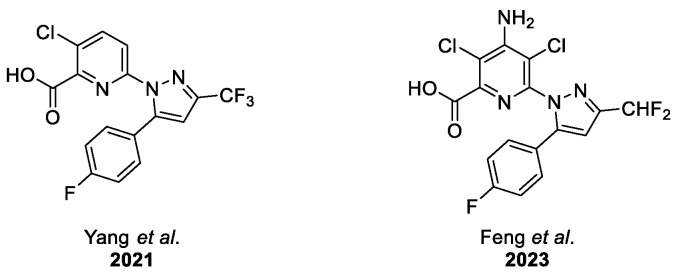
Structures of herbicidal compounds 6-phenylpyrazolyl-2-picolinic acids [18,19].

Many of the researchers also tended to introduce heterocycles or bicyclic heterocycles at the 6 position of 2-picolinic acid to develop highly active and environment-friendly herbicides. For instance, Bayer Crop Science, Corteva Agriscience, etc., introduced benzothiazole, benzofuran, indole, isoxazoline and other heterocycles at the 6 position of 2-picolinic acid [15,16,17]. In our group, we used the 1*H*-pyrazole group to replace the chlorine atom at the 6 position of clopyralid and picloram to obtain new chemotype compounds (Figure 2), and some of the resulted compounds displayed a wider herbicidal spectrum and good crop safety [18,19].

**Figure 3:**
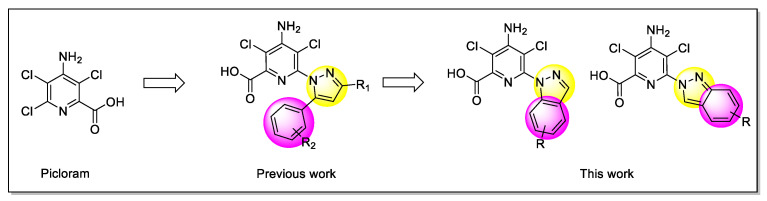
Design strategy of new 6-indazolyl-2-picolinic acid target compounds.

Indazole is a 10π electron aromatic heterocyclic ring with a unique electronic structure and chemical properties [20,21] and is a potential fragment in herbicidal compounds [22,23,24]. In our previous study, some 6-(5-substitued phenylpyrazolyl)-2-picolinic acids were found to have herbicidal activities. In addition, modifying 2-picolinic acid could alter the binding mode of lead compounds at the auxinic herbicide binding pocket. To discover potential herbicidal molecules with low resistance, we further modified 6-(5-substitued phenylpyrazolyl)-2-picolinic acids by replacing the pyrazolyl group with an indazolyl group (Figure 3).

The method of exploring the expression of auxin-related genes gave rise to researchers’ extensive attention to study active compounds’ action mechanism. In 2000, BASF [25] reported that the 1-aminocyclopropane-1-carboxylic acid synthase (ACS) activity, level of 1-aminocyclopropane-1-carboxylic acid (ACC) and ethylene significant increase in *Galium spurium* within 2 h after the application of high concentrations of IAA and picloram. The treatment also aroused the up-regulation of 9-cis-epoxy urea dioxygenase (NCED), which triggered abscisic acid (ABA) to increase 24 times compared to the control group after 24 h. Jiaqi Xu et al. [26] also found that halauxifen-methyl induced over expressions of ACS and NCED genes. Upregulated genes destroyed the homeostasis of IAA and stimulated the excessive production of ethylene and ABA, eventually leading to plant death. In 2019, Lei et al. [27] reported that genes IAA5 (auxin-induce gene), GH3.3 (auxin-regulate gene) and AUX1 (auxin-influx gene) were up-regulated after *Arabidopsis thaliana* (*A. thaliana*) was treated by a synthetic compound [28,29,30,31]. Therefore, the response of auxin-related genes was used for the initial investigation of the mechanism of action of 6-indazolyl-2-picolinic in this study.

## 2. Results and Discussion

### 2.1. Chemistry

**Scheme 1:**
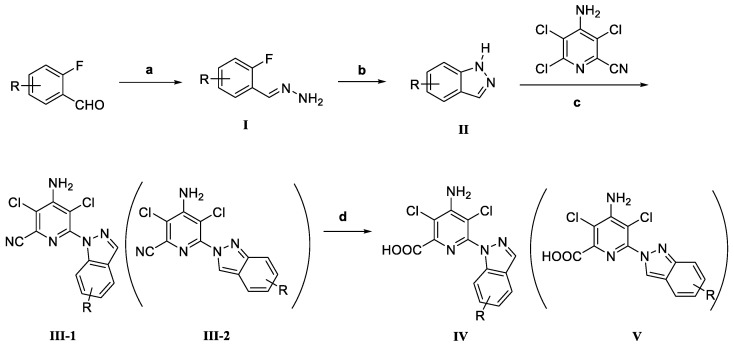
Synthetic route to 6-indazolyl-2-picolinic acids. Reagent and conditions: (**a**) NH_2_-NH_2_·H_2_O, THF, 65 °C, 2 h; (**b**) NaHCO_3_, NH_2_-NH_2_·H_2_O, 100 °C, 4 h; (**c**) NaH, extra dry 1,4-dioxane, 100 °C, 12 h; (**d**) 80% H_2_SO_4_, H_2_O, 100 °C, 3 h.

A series of new 6-indazolyl-2-picolinic acids **1A**–**7d** (Table 1) were provided via the route illustrated in Scheme 1 (some of them are mixtures). All the obtained compounds were characterized via ^1^H NMR, ^13^C NMR, and HRMS.

The substituted indazoles as intermediates were synthesized in two steps: (1) substituted *o*-fluorobenzaldehyde reacts with hydrazine hydrate in tetrahydrofuran to generate corresponding crude substituted benzylidenehydrazine; (2) intermediate indazole forms in the presence of two equivalents of sodium bicarbonate. At the beginning, substituted salicylaldehydes were used as starting materials, and intermediate benzylidenehydrazine could be easily obtained. However, the subsequent cyclization reaction for forming indazole could not proceed. Instead, when *o*-fluorobenzaldehyde was used as the starting material, the cyclization reaction could proceed smoothly by using 100% hydrazine hydrate as a solvent [32]. 1*H*-Indazole and 2*H*-indazole are tautomeric isomers, and 1*H*-indazole is the most significant one, since it is thermodynamically more stable than 2*H*-indazole. In the coupling reaction of 1*H*-indazole and 4-amino-3,5,6-chloro-2-picolinonitrile, sodium salt of 1*H*-indazole would inevitably transform to sodium salt of 2*H*-indazole at room temperature or above; thus, the isomer 4-amino-3,5-dichloro-6-(2*H*-indazolyl)-2-picolinonitrile could form alongside the designed product 4-amino-3,5-dichloro-6-(1*H*-indazolyl)-2-picolinonitrile.

When the substituent is at the 7 position of the indazole ring, only compound **V** containing 2*H*-indazolyl formed probably due to the steric effect. And when the R substituents CH_3_, F, Cl and Br were at the 6 position of 1*H*-indazole, the obtained product isomers were difficult to separate by column chromatography, and their biological activities were tested with their mixtures.

**Scheme 2:**
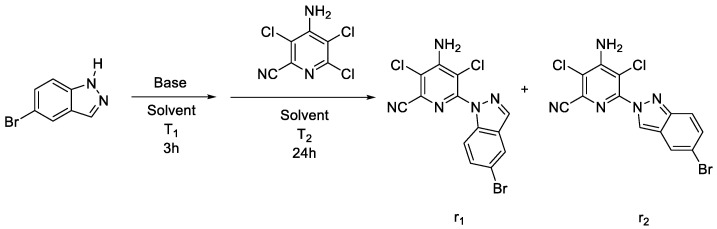
Reaction of 5-bromo-1*H*-indazole and 4-amino-3,5,6-trichloro-2-picolinonitrile.

The preliminary results of the subsequent biological assay to inhibit *A. thaliana* root growth showed that the biological activity of the new compounds containing the 1*H-*indazolyl fragment was better than those with the 2*H-*indazolyl fragment. Thus, we attempted to optimize the conditions of the coupling reaction for the synthesis of 6-(1*H-*indazolyl)-2-picolinonitrile (r_1_) (Scheme 2).

The results in Table 2 showed that inorganic bases potassium carbonate, potassium hydroxide and sodium hydroxide were uncapable of converting 1*H*-indazole to its salt due to the weak alkalinity or low solubility. Cesium carbonate with its better solubility could make the reaction proceed despite the fact that the starting materials could not be fully converted. We also found that the reaction temperature of salt formation influences the ratio of r_1_ and r_2_. For instance, the proportion of compound r_1_ can be significantly increased with the decreasing in T_1_, but the proportion of r_1_ cannot be increased by reducing T_2_. In order to further increase the proportion of r_1_, the solvent was changed to acetonitrile, of which the melting point is much lower than that of 1,4-dioxane. However, the lower boiling point acetonitrile limited the temperature of the reaction and thus resulted in lower conversion. The ratio of r_1_ was increased to 82.9% when 1,2-dimethoxyethane, which has a higher boiling point, was employed as solvent and cesium carbonate was used as the base, but 4-amino-3,5,6-trichloro-2-picolinonitrile could still not be fully consumed. When changing the solvent to 1,2-diethoxyethane with a much higher boiling point, more r_2_ was generated and the reaction was still incomplete, albeit less amounts of 5-bromo-1*H*-indazole and 4-amino-3,5,6-trichloro-2-picolinonitrile were left compared to other conditions.

In the above-mentioned experiments, it was unable to obtain a single isomer as the product, and the reaction could also not be completed. Finally, sodium hydride was employed as the base, and the obtained r_1_ and r_2_ were separated by column chromatography. For cases in which r_1_ and r_2_ cannot be easily separated, the mixture of r_1_ and r_2_ was used in biological activity investigation.

### 2.2. Phenotypic Study of Arabidopsis thaliana and SAR Analysis

**Figure 4:**
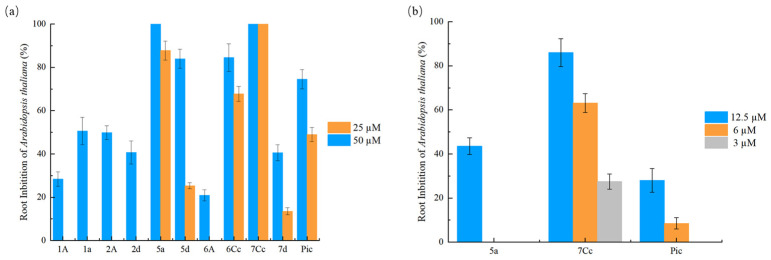
The inhibition activity of *A. thaliana* root growth for some compounds. (**a**): the assay concentrations were 50 µM and 25 µM; (**b**): the assay concentrations were 12.5 µM, 6 µM and 3 µM.

All new compounds were tested against *A. thaliana* root growth at concentrations from 200 µmol/L to 3 µmol/L. When the inhibition at a certain concentration (µmol/L) is greater than 80%, the test was continued at half the concentration used. The inhibition effect of some new compounds, picloram and DMSO (solvent), at different concentrations against *A. thaliana* root growth is shown in Figure 4.

The results from Figure 4a show that compounds **5a**, **6Cc**, and **7Cc** displayed significant inhibitory activity against *A. thaliana* root growth and were better than the commercial herbicide picloram at the concentrations 50 and 25 µM. Figure 4b shows that **7Cc** at 3 µM had the same inhibition effect with picloram at 12.5 µM. Structure–activity relationship analysis revealed that the electron-donating substituents amino and methoxy in indazolyl decreased the inhibition activity of new compounds; when the substituents are proton, methyl, and halide atoms, the inhibition activity of compound **IV** was better than that of compound **V**; when the substituents are at positions 4, 6 and 7 on the indazole ring, the new compounds have similar inhibition activity, and their inhibition activity was better than those at positions 5; when the indazole ring was substituted by electron-withdrawing groups, the inhibition activity was significantly improved. The influence of halide atoms on inhibition activity was related to their electronegativity and weaker electronegativity resulted in higher inhibition activity. Overall, the substituents in indazole ring improve the inhibition activity of the compounds in the following order: bromine > chlorine > fluorine ≈ methyl > amino > methoxy.

### 2.3. Evaluation of Herbicidal Activity

#### 2.3.1. Root Growth Inhibition of Weeds in Petri Dishes

**Figure 5:**
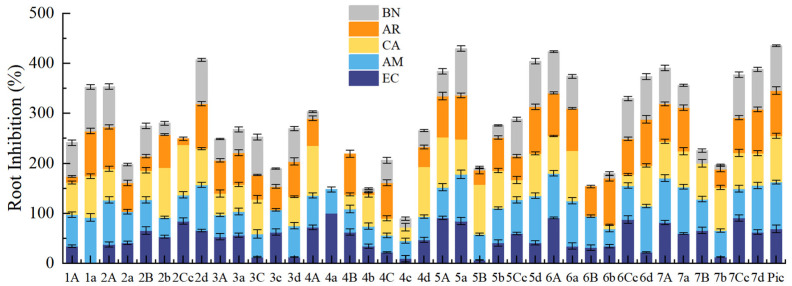
The root inhibition activity of new compounds on five grasses at 500 µM.

**Figure 6:**
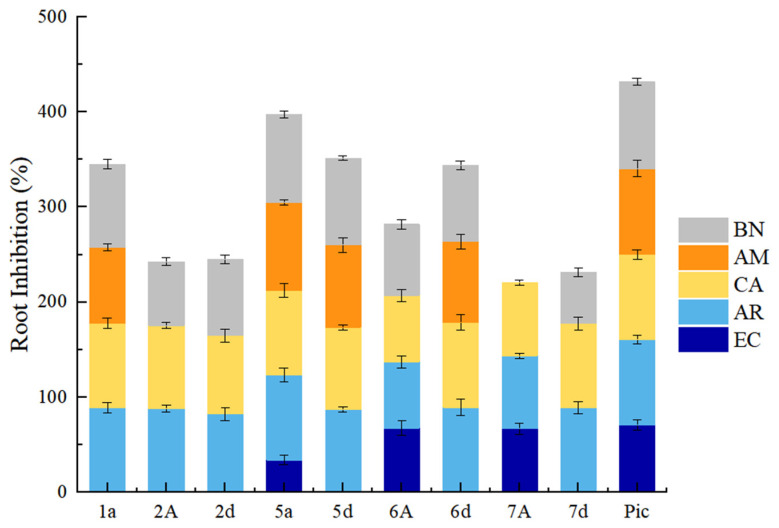
The root inhibition activity of new compounds on five grasses at 250 µM.

The herbicidal activity of new compounds was evaluated according to a reported procedure [18], in which picloram was used as the control, and each experiment had three replicates. Compounds **1A**–**7d** were tested to evaluate their effect to control the root growth of five grass seeds including *Echinochloa crusgalli* (EC), *Amaranthus retroflexus* (AR), *Chenopodium album* (CA), *Abutilon theophrasti Medicus* (AM) and *Brassica napus* (BN) at concentrations of 500 µM (Figure 5) and 250 µM (Figure 6).

The results showed that most compounds have a certain inhibitory effect on the roots of weeds but their inhibitory activity on EC was generally weak. The relationship of structure–activity showed that the positions of substituents are related with the root inhibitory activity of weeds. The inhibitory effect is much better with substituents on the 4 position of the indazole ring, while the 5 position substitution results in poor inhibitory activity. In addition, compounds with electron-withdrawing substituents on the indazole ring showed better activity. There is no significant difference in inhibitory activities between 1*H*- and 2*H*-indazolyl isomers.

**Figure 7:**
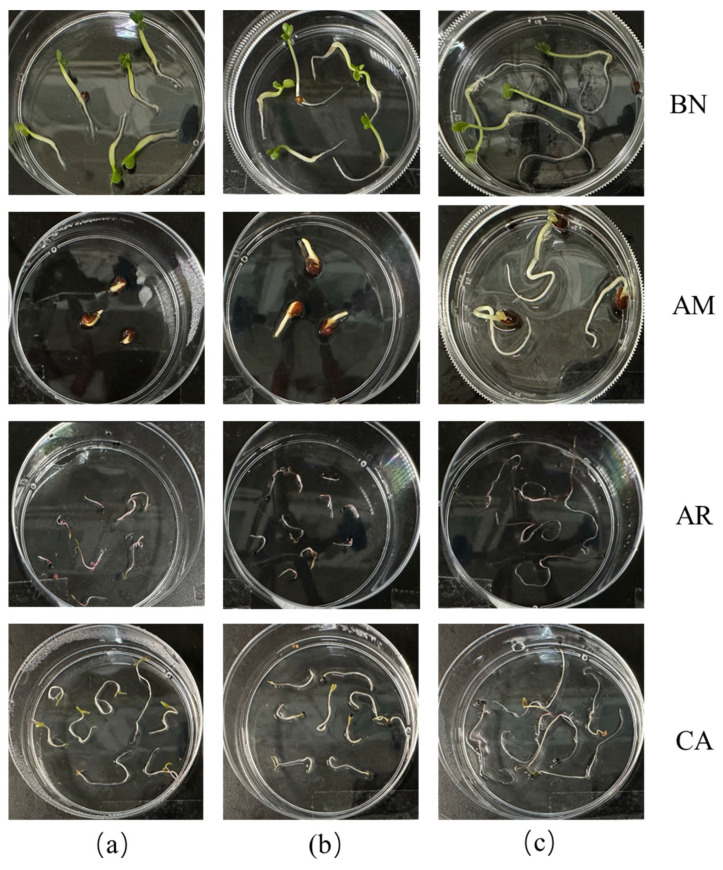
The weed root growth inhibition activity. ((**a**): **5a**; (**b**): picloram; (**c**): water).

Moreover, compound **5a** had a similar inhibitory effect compared to picloram, and their root growth inhibition on four dicotyledonous grasses at 10 µM/L was tested (Figure 7).

Compound **5a** had a higher inhibitory activity on the root growth of BN (like *A. thaliana*, a member of the Cruciferae family) compared to picloram when the concentration was decreased to 10 μM, and the inhibitory activity of compound **5a** on the roots of AM was also significantly higher than picloram. For CA and AR, two species of weeds in the Amaranthaceae family, the inhibitory effect of compound **5a** was not as good as that of picloram.

#### 2.3.2. Herbicidal Activity

**Figure 8:**
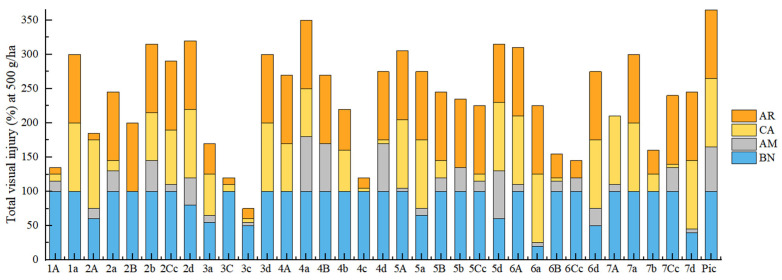
Summary of the visual injury percentages resulting from treatment of the four dicotyledonous weeds by the compounds over 14 days at the concentration of 500 g/ha.

In addition, the herbicidal activities of the 38 new compounds against the four dicotyledonous weeds and one monocotyledonous weed were tested in a glasshouse. The test was carried out at a range of concentrations (from high to low) until the visual injury effect was less than 60%. Some results are displayed in Table 3 and Figure 8, and the full results can be found in Table S1.

Most compounds exhibit excellent herbicidal activity at 1000 g/ha, and some of them (as shown in Table 3) could completely control BN, CA and AR at a dosage of 250 g/ha. The herbicidal activities of new compounds containing a 4 position substituent were superior to those containing the 7 position substituent and to those with substituent at the 5 and 6 position. The electronic properties of the substituent did not have a significant effect on the activity.

### 2.4. The Response of Auxin Relative Genes

**Figure 9:**
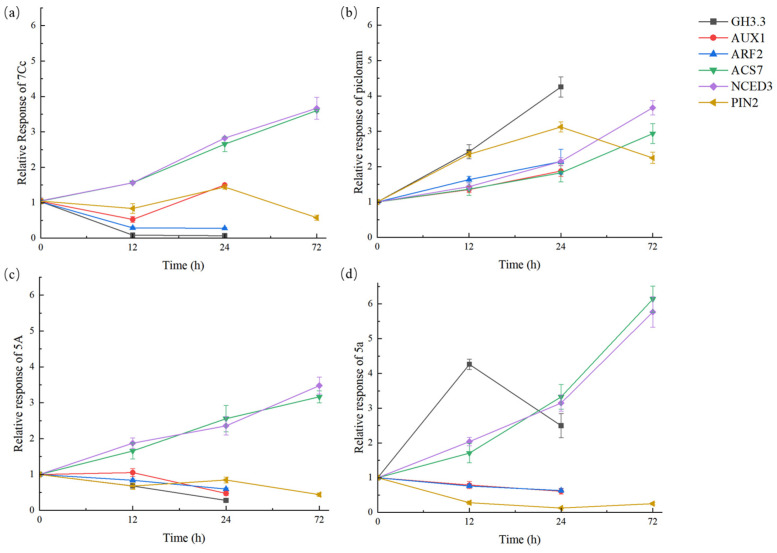
Auxin-related genes response. ((**a**): compound **7Cc**; (**b**): picloram; (**c**): compound **5A**; (**d**): compound **5a**).

Compound **7Cc** had superior inhibitory activity against the root growth of *A. thaliana* compared to picloram, and the evaluation of auxin-related gene response was carried out by treating *A. thaliana* with compound **7Cc** (Figure 9a). The results showed that compound **7Cc** did not upregulate AUX1 (auxin-influx gene), PIN2 (auxin-efflux gene) [33], GH3.3 (auxin-regulate gene), or ARF2 (auxin response factor) as picloram did (Figure 9b), but upregulated the expression of ACS7 and NCED3 genes and promoted the production of ethylene and ABA, which affect the physiological processes of plant growth. In order to figure out the impact of each isomer of 7Cc on the auxin gene response (Figure 9b), compounds **5A** and **5a** were employed to explore the auxin-related gene response, respectively.

The AUX1, PIN2, and ARF2 genes’ expression of *A. thaliana* treated with compounds **5A** and **5a** (Figure 9c,d) remained down-regulated, implying that they are unable to be transported via carrier proteins as traditional commercial picolinic herbicides, and they are unable to bind auxin receptor proteins TIR1 and AFBs to release ARFs [34,35]. Compound **5a** induced a higher level of ACS7 and NCED3 expression compared to **5A**, explaining why it inhibited *A. thaliana* root growth better than compound **5A**.

The new synthesized compounds also contain the 2-picolinic acid skeleton, promote ethylene release and ABA production by causing up-regulation of ACS and NCED genes, leading to the death of the treated plant in a short time. Such an action mechanism is different from those of other 2-picolinic acid herbicides, which might mitigate the potential growth of resistance. However, the exact mechanism of action remains unclear.

## 3. Materials and Methods

### 3.1. Chemicals, Experimental Instruments and Plant Materials

Solvents and reagents were provided by Bide Pharmatech (Beijing, China). The commercial herbicide picloram was provided by Nutrichem Company Ltd. (Beijing, China). *Arabidopsis thaliana* (*A. thaliana* ecotype Columbia-0, Col-0) and weed seeds were provided by the Laboratory of National Forestry and Grassland Administration on Pest Chemical Control, China Agricultural University, Beijing, China. ^1^H NMR and ^13^C NMR spectra were obtained at 500 MHz using a Bruker AVANCE NOE500 spectrometer (Billerica, MA, USA) in DMSO-*d*_6_ solution. HRMS was performed using an Agilent 6540 Q-TOF instrument (Santa Clara, CA, USA) instrument. The *A. thaliana* and weed root growth data were obtained using IMAGEJ software (https://imagej.nih.gov/ij/).

### 3.2. Synthesis

#### 3.2.1. General Synthetic Procedure of Intermediates **II**

Compound **I** (100 mmol) and tetrahydrofuran (250 mL) were added into a 500 mL three-mouth round-bottom flask at 25 °C, and 80% hydrazine hydrate (110 mmol) was added drop-wise to the reaction solution under stirring. Subsequently, the reaction mixture was heated to 65 °C and maintained at this temperature for 2 h. Then, the mixture was cooled to 25 °C, and concentrated under a vacuum to obtain the crude-substituted benzylidenehydrazine. This crude intermediate was dissolved in 100% hydrazine hydrate (100 mL), Na_2_CO_3_ (200 mmol) was then added under stirring at 25 °C, and the reaction mixture was heated to 100 °C and maintained at this temperature for 4 h. After the reaction was completed, the mixture was cooled to 25 °C, quenched and acidified to pH 5–6 with an aqueous hydrochloric acid solution, and extracted using ethyl acetate (3 × 15 mL). The combined organic phase was dried over anhydrous sodium sulfate, filtered, and concentrated under a vacuum. The residue was purified via flash column chromatography (n-hexane/ethyl acetate = 15:1) to afford intermediate **II** (yields 78.5–90.3%).

#### 3.2.2. General Synthetic Procedure of Intermediate **III**

In a 50 mL three-mouth round-bottom flask, sodium hydride (60%, 8.96 mmol) was added to extra dry 1,4-dioxane (15 mL), and compound **II** (5.60 mmol) in extra dry 1,4-dioxane (10 mL) was added drop-wise under stirring at 25 °C. The reaction mixture was heated to 50 °C and maintained at this temperature for 3 h. Then, 4-amino-3,5,6-trichloropicolinonitrile (5.60 mmol) was added under stirring and heated to 100 °C for 12 h. The reaction solution was cooled to 25 °C, and was quenched with water. The solid was filtered to provide a mixture of compound **III** 1 and compound **III** 2, which were separated via flash column chromatography (n-hexane/ethyl acetate = 10:1) to obtain compounds **III** 1 (yields 37.6–42.4%) and compounds **III** 2 (yields 35.2–45.0%).

#### 3.2.3. General Synthetic Procedure of Product

In a 25 mL round-bottom flask, compound **III** (1.067 mmol) was dissolved in 80% aqueous sulfuric acid (10 mL), and was heated to 100 °C and maintained at this temperature for 2 h. The reaction solution was cooled to 25 °C, and quenched with water. A white solid was collected through filtration to obtain the product (yields 90.1–99.0%).

Compound **1A** 4-amino-3,5-dichloro-6-(1*H*-indazol-1-yl)-2-picolinic acid. White solid; 181.9–182.7 °C; ^1^H NMR (500 MHz, DMSO-*d*_6_) δ 13.86 (s, 1H), 8.40 (s, 1H), 7.89 (d, *J* = 8.1 Hz, 1H), 7.58 (d, *J* = 8.1 Hz, 1H), 7.50–7.45 (m, 1H), 7.30–7.26 (m, 3H). ^13^C NMR (126 MHz, DMSO-*d*_6_) δ 166.09, 151.20, 146.84, 146.40, 139.92, 136.57, 127.93, 124.58, 122.49, 121.62, 111.93, 111.48, 110.45. HRMS calcd. For C_13_H_7_Cl_2_N_4_O_2_ ([M – H]^−^), 320.9946; found, 320.9945.

Compound **1a** 4-amino-3,5-dichloro-6-(2*H*-indazol-2-yl)-2-picolinic acid. White solid; 185.1–185.6 °C; H NMR (500 MHz, DMSO-*d*_6_) δ 13.88 (s, 1H), 8.77 (s, 1H), 7.80 (d, *J* = 8.5 Hz, 1H), 7.71 (d, *J* = 8.8 Hz, 1H), 7.41 (s, 2H), 7.34 (dd, *J* = 6.9, 1.6 Hz, 1H), 7.13 (dd, *J* = 6.9, 1.6 Hz, 1H). ^13^C NMR (126 MHz, DMSO-*d*_6_) δ 165.90, 151.00, 149.08, 147.30, 146.75, 127.52, 125.98, 122.75, 121.69, 118.07, 112.44, 110.58. HRMS calcd. For C_13_H_7_Cl_2_N_4_O_2_ ([M – H]^−^), 320.9946; found, 320.9947.

Compound **2A** 4-amino-3,5-dichloro-6-(4-methyl-1*H*-indazol-1-yl)-2-picolinic acid. White solid; 195.2–196.4 °C; ^1^H NMR (500 MHz, DMSO-*d*_6_) δ 13.95 (s, 1H), 8.81 (s, 1H), 7.51 (d, *J* = 8.7 Hz, 1H), 7.42 (s, 2H), 7.24 (dd, *J* = 8.8, 6.7 Hz, 1H), 6.88 (d, *J* = 6.7 Hz, 1H), 2.53 (s, 3H). ^13^C NMR (126 MHz, DMSO-*d*_6_) δ 166.09, 151.15, 146.87, 146.50, 139.89, 135.54, 131.68, 128.03, 124.90, 122.34, 111.44, 110.53, 109.25, 18.63. HRMS calcd. For C_14_H_9_Cl_2_N_4_O_2_ ([M – H]^−^), 335.0103; found, 335.0103.

Compound **2a** 4-amino-3,5-dichloro-6-(4-methyl-2*H*-indazol-2-yl)-2-picolinic acid. White solid; 198.9–201.1 °C; ^1^H NMR (500 MHz, DMSO-*d*_6_) δ 13.31 (s, 1H), 8.81 (s, 1H), 7.57 (d, *J* = 7.0 Hz, 1H), 7.51 (d, *J* = 8.8 Hz, 1H), 7.41 (s, 2H), 7.23 (dd, *J* = 8.8, 6.7 Hz, 1H), 6.91–6.85 (m, 1H). ^13^C NMR (126 MHz, DMSO-*d*_6_) δ 168.24, 153.85, 146.47, 143.92, 138.24, 134.72, 130.70, 129.35, 126.83, 120.32, 114.89, 110.58, 105.08, 20.43. HRMS calcd. For C_14_H_9_Cl_2_N_4_O_2_ ([M − H]^−^), 335.0103; found, 335.0104.

Compound **2B** 4-amino-3,5-dichloro-6-(5-methyl-1*H*-indazol-1-yl)-2-picolinic acid. White solid; 190.8–192.2 °C; ^1^H NMR (500 MHz, DMSO-*d*_6_) δ 12.74 (s, 1H), 8.30 (d, *J* = 0.7 Hz, 1H), 7.65–7.62 (m, 1H), 7.49 (d, *J* = 8.5 Hz, 1H), 7.31 (dd, *J* = 8.7, 1.3 Hz, 1H), 7.28 (s, 2H), 2.44 (s, 3H). ^13^C NMR (126 MHz, DMSO-*d*_6_) δ 166.10, 151.17, 146.79, 146.48, 138.60, 136.05, 131.59, 129.77, 124.99, 120.49, 111.74, 111.32, 110.19, 21.29. HRMS calcd. For C_14_H_9_Cl_2_N_4_O_2_ ([M − H]^−^), 335.0103; found, 335.0103.

Compound **2b** 4-amino-3,5-dichloro-6-(5-methyl-2*H*-indazol-2-yl)-2-picolinic acid. White solid; 193.6–192.2 °C; ^1^H NMR (500 MHz, DMSO-*d*_6_) δ 13.11 (s, 1H), 8.63 (s, 1H), 7.61 (d, *J* = 8.9 Hz, 1H), 7.52 (s, 1H), 7.39 (s, 2H), 7.18 (dd, *J* = 8.9, 1.4 Hz, 1H), 2.39 (s, 3H). ^13^C NMR (126 MHz, DMSO-*d*_6_) δ 165.91, 150.98, 148.15, 147.29, 146.72, 131.65, 130.47, 124.91, 121.96, 119.36, 117.84, 112.28, 110.41, 21.84. HRMS calcd. For C_14_H_9_Cl_2_N_4_O_2_ ([M − H]^−^), 335.0103; found, 335.0101.

Compound **2Cc** (mixture) 4-amino-3,5-dichloro-6-(6-methyl-1*H*-indazol-1-yl)-2-picolinic acid and 4-amino-3,5-dichloro-6-(6-methyl-2*H*-indazol-2-yl)-2-picolinic acid (1: 1.27). Yellow solid; 149.5–160.2 °C; ^1^H NMR (500 MHz, DMSO-d6) δ 13.27 (s, 1H), 8.34 (s, 1H), 7.93 (s, 1H), 7.83 (s, 1H), 7.30 (s, 2H), 2.45 (s, 3H). ^13^C NMR (126 MHz, DMSO-d6) δ 165.83, 151.00, 146.53, 145.97, 136.24, 131.04, 125.24, 124.92, 124.26, 122.30, 115.36, 111.65, 110.16, 23.06; ^1^H NMR (500 MHz, DMSO-*d*_6_) δ 12.76 (s, 1H), 8.72 (s, 1H), 8.04 (s, 1H), 7.77 (s, 1H), 7.41 (s, 2H), 2.42 (s, 3H). ^13^C NMR (126 MHz, DMSO-*d*_6_) δ 166.01, 151.27, 148.74, 146.95, 146.65, 139.18, 131.11, 125.88, 121.80, 121.10, 120.79, 112.61, 110.52, 23.48. HRMS calcd. For C_14_H_9_Cl_2_N_4_O_2_ ([M − H]^−^), 335.0103; found, 335.0105.

Compound **2d** 4-amino-3,5-dichloro-6-(7-methyl-2*H*-indazol-2-yl)-2-picolinic acid. White solid; 190.8–191.6 °C; ^1^H NMR (500 MHz, DMSO-*d*_6_) δ 13.94 (s, 1H), 8.73 (s, 1H), 7.60 (d, *J* = 8.4 Hz, 1H), 7.41 (s, 2H), 7.10 (d, *J* = 6.6 Hz, 1H), 7.03 (dd, *J* = 8.4, 6.7 Hz, 1H), 2.53 (s, 3H). ^13^C NMR (126 MHz, DMSO-*d*_6_) δ 165.92, 150.95, 149.55, 147.48, 146.78, 127.66, 126.15, 126.12, 123.01, 121.46, 118.99, 112.41, 110.76, 17.29. HRMS calcd. For C_14_H_9_Cl_2_N_4_O_2_ ([M − H]^−^), 335.0103; found, 335.0101.

Compound **3A** 4-amino-3,5-dichloro-6-(4-amino-1*H*-indazol-1-yl)-2-picolinic acid. Pale yellow solid; 170.5–171.4 °C; ^1^H NMR (500 MHz, DMSO-*d*_6_) δ 13.34 (s, 1H), 8.39 (s, 1H), 7.24 (s, 2H), 7.08 (dd, *J* = 8.0, 7.5 Hz, 1H), 6.58 (d, *J* = 8.2 Hz, 1H), 6.28 (d, *J* = 7.5 Hz, 1H). ^13^C NMR (126 MHz, DMSO-*d*_6_) δ 166.17, 151.01, 146.90, 143.01, 141.58, 135.01, 129.44, 114.30, 111.13, 110.55, 103.73, 98.37. HRMS calcd. For C_13_H_8_Cl_2_N_5_O_2_ ([M − H]^−^), 336.0055; found, 336.0056.

Compound **3a** 4-amino-3,5-dichloro-6-(4-amino-2*H*-indazol-2-yl)-2-picolinic acid. Pale yellow solid; 179.3–180.2 °C; ^1^H NMR (500 MHz, DMSO-*d*_6_) δ 14.03 (s, 0H), 11.14 (s, 1H), 8.98 (s, 2H), 7.82 (dd, *J* = 8.2, 0.9 Hz, 1H), 7.07 (dd, *J* = 8.2, 8.1 Hz, 1H), 6.60 (s, 2H), 6.35 (dd, *J* = 8.1, 0.8 Hz, 1H). ^13^C NMR (126 MHz, DMSO-*d*_6_)13C NMR (126 MHz, DMSO-d6) δ 171.14, 166.86, 151.82, 149.35, 148.38, 145.27, 143.65, 133.20, 109.89, 106.39, 105.05, 100.59, 99.87. HRMS calcd. For C_13_H_8_Cl_2_N_5_O_2_ ([M − H]^−^) 336.0055; found, 336.0057.

Compound **3C** 4-amino-3,5-dichloro-6-(6-amino-1*H*-indazol-1-yl)-2-picolinic acid. Pale yellow solid; 172.3–173.3 °C; ^1^H NMR (500 MHz, DMSO-*d*_6_) δ 13.42 (s, 1H), 8.02 (s, 1H), 7.46 (d, *J* = 8.6 Hz, 1H), 7.23 (s, 2H), 6.59 (dd, *J* = 8.6, 1.5 Hz, 1H), 6.44 (s, 1H). ^13^C NMR (126 MHz, DMSO-*d*_6_) δ 166.18, 150.93, 149.54, 147.06, 142.29, 136.33, 121.69, 116.36, 113.50, 111.06, 110.82, 92.11. HRMS calcd. For C_13_H_8_Cl_2_N_5_O_2_ ([M − H]^−^), 336.0055; found, 336.0060.

Compound **3c** 4-amino-3,5-dichloro-6-(6-amino-2*H*-indazol-2-yl)-2-picolinic acid. Pale yellow solid; 158.9–160.0 °C; ^1^H NMR (500 MHz, DMSO-*d*_6_) δ 13.35 (s, 1H), 8.92 (s, 1H), 7.99 (d, *J* = 8.9 Hz, 1H), 7.73 (s, 1H), 7.46 (s, 2H), 7.10 (dd, *J* = 8.9, 1.4 Hz, 1H). ^13^C NMR (126 MHz, DMSO-d6) δ 165.83, 151.03, 148.01, 146.90, 146.75, 131.20, 127.30, 124.22, 120.75, 118.79, 112.72, 111.92, 110.63. HRMS calcd. For C_13_H_8_Cl_2_N_5_O_2_ ([M − H]^−^), 335.0055; found, 336.0062.

Compound **3d** 4-amino-3,5-dichloro-6-(7-amino-2*H*-indazol-2-yl)-2-picolinic acid. Yellow solid; 214.2–214.9 °C; ^1^H NMR (500 MHz, DMSO-*d*_6_) δ 13.23 (s, 1H), 8.55 (s, 1H), 7.39 (s, 2H), 6.91 (d, *J* = 8.0 Hz, 1H), 6.85 (dd, *J* = 8.3, 7.1 Hz, 1H), 6.33 (d, *J* = 6.9 Hz, 1H). ^13^C NMR (126 MHz, DMSO-*d*_6_) δ 166.00, 150.87, 147.53, 146.88, 142.90, 138.72, 125.29, 124.53, 122.48, 112.21, 110.66, 107.65, 104.43. HRMS calcd. For C_13_H_8_Cl_2_N_5_O_2_ ([M − H]^−^), 335.0055; found, 336.0057.

Compound **4A** 4-amino-3,5-dichloro-6-(4-methoxy-1*H*-indazol-1-yl)-2-picolinic acid. White solid; 199.1–201.6 °C; ^1^H NMR (500 MHz, DMSO-*d*_6_) δ 13.36 (s, 1H), 9.16 (s, 1H), 7.95 (d, *J* = 8.3 Hz, 1H), 7.38 (dd, *J* = 8.9, 8.4 Hz, 1H), 6.75 (d, *J* = 8.3 Hz, 1H), 6.72 (s, 2H), 3.81 (s, 3H). ^13^C NMR (126 MHz, DMSO-*d*_6_) δ 169.31, 166.67, 158.44, 148.88, 148.62, 145.36, 140.35, 132.17, 112.99, 111.66, 105.79, 105.48, 99.33, 56.46. HRMS calcd. For C_14_H_9_Cl_2_N_4_O_3_ ([M − H]^−^), 351.0052; found, 351.0052.

Compound **4a** 4-amino-3,5-dichloro-6-(4-methoxy-2*H*-indazol-2-yl)-2-picolinic acid. White solid; 221.3–222.5 °C; ^1^H NMR (500 MHz, DMSO-*d*_6_) δ 13.56 (s, 1H), 7.95 (s, 1H), 7.18 (dd, *J* = 8.0, 7.9 Hz, 1H), 7.02 (d, *J* = 8.3 Hz, 1H), 6.47 (s, 1H), 6.46 (s, 2H), 3.84 (s, 3H). ^13^C NMR (126 MHz, DMSO-*d*_6_) δ 169.12, 157.35, 150.28, 145.45, 142.77, 135.39, 131.83, 128.28, 124.79, 118.32, 112.67, 105.39, 101.68, 56.92. HRMS calcd. For C_14_H_9_Cl_2_N_4_O_3_ ([M − H]^−^), 351.0052; found, 351.0050.

Compound **4B** 4-amino-3,5-dichloro-6-(5-methoxy-1*H*-indazol-1-yl)-2-picolinic acid. White solid; 179.8–180.5 °C; ^1^H NMR (500 MHz, DMSO-*d*_6_) δ 13.72 (s, 1H), 8.28 (s, 1H), 7.53 (d, *J* = 9.1 Hz, 1H), 7.31 (d, *J* = 2.3 Hz, 1H), 7.28 (s, 2H), 7.13 (dd, *J* = 9.1, 2.4 Hz, 1H), 3.83 (s, 3H). ^13^C NMR (126 MHz, DMSO-*d*_6_) δ 166.12, 155.49, 151.19, 146.89, 146.42, 135.98, 135.62, 125.25, 119.32, 113.20, 111.16, 109.80, 101.16, 55.96. HRMS calcd. For C_14_H_9_Cl_2_N_4_O_3_ ([M − H]^−^), 351.0052; found, 351.0051.

Compound **4b** 4-amino-3,5-dichloro-6-(5-methoxy-2*H*-indazol-2-yl)-2-picolinic acid. White solid; 223.0–224.3 °C; ^1^H NMR (500 MHz, DMSO-*d*_6_) δ13.67 (s, 1H), 8.58 (d, *J* = 0.9 Hz, 1H), 7.62 (d, *J* = 9.3 Hz, 1H), 7.38 (s, 2H), 7.05 (d, *J* = 2.4 Hz, 1H), 7.02 (dd, *J* = 9.3, 2.4 Hz, 1H), 3.80 (s, 3H). ^13^C NMR (126 MHz, DMSO-*d*_6_) δ 168.10, 157.32, 150.76, 148.85, 142.45, 130.63, 129.46, 125.82, 120.97, 115.23, 110.35, 104.74, 100.38, 55.52. HRMS calcd. For C_14_H_9_Cl_2_N_4_O_3_ ([M − H]^−^), 351.0052; found, 351.0056.

Compound **4C** 4-amino-3,5-dichloro-6-(6-methoxy-1*H*-indazol-1-yl)-2-picolinic acid. White solid; 193.1–194.4 °C; ^1^H NMR (500 MHz, DMSO-*d*_6_) δ 12.98 (s, 1H), 11.51 (s, 1H), 8.77 (d, *J* = 2.5 Hz, 1H), 7.94 (d, *J* = 8.9 Hz, 1H), 6.76 (s, 2H), 6.57 (dd, *J* = 8.9, 2.6 Hz, 1H), 3.82 (s, 3H). ^13^C NMR (126 MHz, DMSO-*d*_6_) δ 170.35, 166.52, 164.08, 148.87, 148.77, 145.82, 144.15, 133.37, 107.49, 107.27, 106.68, 102.96, 100.25, 55.63. HRMS calcd. For C_14_H_9_Cl_2_N_4_O_3_ ([M − H]^−^), 351.0052; found, 351.0056.

Compound **4c** 4-amino-3,5-dichloro-6-(6-methoxy-2*H*-indazol-2-yl)-2-picolinic acid. White solid; 244.6–245.0 °C; ^1^H NMR (500 MHz, DMSO-*d*_6_) δ 13.05 (s, 1H), 8.59 (s, 1H), 7.65 (d, *J* = 9.1 Hz, 1H), 7.07 (s, 2H), 6.99 (s, 1H), 6.77 (dd, *J* = 9.1, 2.1 Hz, 1H), 3.83 (s, 3H). 13C NMR (126 MHz, DMSO-*d*_6_) δ 166.95, 159.22, 150.34, 150.09, 146.92, 125.71, 122.50, 117.79, 117.45, 110.77, 108.33, 94.92, 55.60. HRMS calcd. For C_14_H_9_Cl_2_N_4_O_3_ ([M − H]^−^), 351.0052; found, 351.0054.

Compound **4d** 4-amino-3,5-dichloro-6-(7-methoxy-2*H*-indazol-2-yl)-2-picolinic acid. White solid; 203.0–203.7 °C; ^1^H NMR (500 MHz, DMSO-*d*_6_) δ 13.97 (s, 1H), 8.71 (s, 1H), 7.41 (s, 2H), 7.31 (d, *J* = 8.4 Hz, 1H), 7.03 (dd, *J* = 8.3, 7.5 Hz, 1H), 6.68 (d, *J* = 7.3 Hz, 1H), 3.93 (s, 3H). ^13^C NMR (126 MHz, DMSO-*d*_6_) δ 165.90, 150.97, 150.50, 147.32, 146.76, 142.78, 125.92, 123.51, 123.42, 113.20, 112.37, 110.60, 104.23, 55.70. HRMS calcd. For C_14_H_9_Cl_2_N_4_O_3_ ([M − H]^−^), 351.0052; found, 351.0060.

Compound **5A** 4-amino-3,5-dichloro-6-(4-fluoro-1*H*-indazol-1-yl)-2-picolinic acid. White solid; 161.2–162.3 °C; ^1^H NMR (500 MHz, DMSO-*d*_6_) δ 12.80 (s, 1H), 8.55 (d, *J* = 0.8 Hz, 1H), 7.50 (td, *J* = 8.0, 5.1 Hz, 1H), 7.42 (d, *J* = 8.4 Hz, 1H), 7.35 (s, 2H), 7.09 (dd, *J* = 10.2, 7.6 Hz, 1H). ^13^C NMR (126 MHz, DMSO-*d*_6_) δ 165.80, 156.63, 154.62, 151.19, 151.15, 151.02, 146.96, 146.77, 127.88, 127.83, 123.63, 123.59, 114.64, 114.60, 113.42, 113.26, 112.79, 110.85, 105.48, 105.35. HRMS calcd. For C_13_H_6_Cl_2_FN_4_O_2_ ([M − H]^−^) 338.9852; found, 338.9859.

Compound **5a** 4-amino-3,5-dichloro-6-(4-fluoro-2*H*-indazol-2-yl)-2-picolinic acid. White solid; 177.9–178.5 °C; ^1^H NMR (500 MHz, DMSO-*d*_6_) δ 13.78 (s, 1H), δ 8.99 (d, *J* = 0.9 Hz, 1H), 7.57 (d, *J* = 8.7 Hz, 1H), 7.46 (s, 2H), 7.34 (ddd, *J* = 8.8, 7.5, 5.4 Hz, 1H), 6.91 (dd, *J* = 10.7, 7.4 Hz, 1H). ^13^C NMR (126 MHz, DMSO-*d*_6_) δ 165.98, 156.14, 154.15, 151.24, 146.87, 145.92, 142.43, 142.36, 132.50, 129.47, 129.41, 114.41, 114.23, 111.98, 110.82, 108.43, 108.40, 107.01, 106.86. HRMS calcd. For C_13_H_6_Cl_2_FN_4_O_2_ ([M − H]^−^) 338.9852; found, 338.9858.

Compound **5B** 4-amino-3,5-dichloro-6-(5-fluoro-1*H*-indazol-1-yl)-2-picolinic acid. White solid; 197.8–198.4 °C; ^1^H NMR (500 MHz, DMSO-*d*_6_) δ 13.23 (s, 1H), 8.40 (d, *J* = 0.9 Hz, 1H), 7.69 (dd, *J* = 8.9, 2.5 Hz, 1H), 7.66 (dd, *J* = 9.2, 4.4 Hz, 1H), 7.39 (ddd, *J* = 9.1, 2.5, 2.3 Hz, 1H), 7.33 (s, 2H). ^13^C NMR (126 MHz, DMSO-*d*_6_) δ 166.01, 159.20, 157.32, 151.28, 146.74, 146.13, 136.95, 136.43, 136.38, 124.86, 124.78, 117.29, 117.08, 113.73, 113.66, 111.61, 110.25, 105.98, 105.79. HRMS calcd. For C_13_H_6_Cl_2_FN_4_O_2_ ([M − H]^−^), 338.9852; found, 338.9850.

Compound **5b** 4-amino-3,5-dichloro-6-(5-fluoro-2*H*-indazol-2-yl)-2-picolinic acid. White solid; 193.5–194.8 °C; ^1^H NMR (500 MHz, DMSO-*d*_6_) δ 14.02 (s, 1H), 8.77 (s, 1H), 7.81 (dd, *J* = 9.4, 4.7 Hz, 1H), 7.54 (dd, *J* = 9.4, 2.2 Hz, 1H), 7.44 (s, 2H), 7.28 (ddd, *J* = 9.3, 2.4, 2.2 Hz, 1H). ^13^C NMR (126 MHz, DMSO-*d*_6_) δ 165.83, 159.17, 157.27, 150.98, 146.93, 146.61, 146.55, 126.48, 126.41, 120.88, 120.78, 120.64, 120.56, 119.35, 119.12, 112.60, 110.49, 104.08, 103.89. HRMS calcd. For C_13_H_6_Cl_2_FN_4_O_2_ ([M − H]^−^), 338.9852; found, 338.9850.

Compound **5Cc** (mixture) 4-amino-3,5-dichloro-6-(6-fluoro-1*H*-indazol-1-yl)-2-picolinic acid and 4-amino-3,5-dichloro-6-(6-fluoro-2*H*-indazol-2-yl)-2-picolinic acid (1: 0.47). Yellow solid; 160.1–167.9 °C; ^1^H NMR (500 MHz, DMSO-*d*_6_) δ 13.94 (s, 1H), 8.77 (s, 1H), 7.80 (dd, *J* = 9.4, 4.7 Hz, 1H), 7.53 (dd, *J* = 9.4, 2.4 Hz, 1H), 7.43 (s, 2H), 7.28 (ddd, *J* = 9.4, 2.5, 2.2 Hz, 1H). ^13^C NMR (126 MHz, DMSO-*d*_6_) δ 166.00, 157.33, 151.30, 146.75, 146.15, 136.40, 124.87, 124.78, 120.78, 120.70, 117.28, 117.06, 113.70, 111.61, 111.23, 105.96, 105.77; ^1^H NMR (500 MHz, DMSO-*d*_6_) δ 13.11 (s, 1H), 8.40 (s, 1H), 7.68 (dd, *J* = 8.9, 2.5 Hz, 1H, 7.65 (dd, *J* = 8.9, 2.5 Hz, 1H), 7.39 (td, *J* = 9.1, 2.5 Hz, 1H), 7.33 (s, 2H). ^13^C NMR (126 MHz, DMSO-*d*_6_) δ 165.85, 159.22, 151.04, 147.08, 146.61, 136.97, 126.43, 126.36, 120.89, 120.80, 119.24, 119.01, 113.77, 112.53, 110.50, 104.04, 103.84. HRMS calcd. For C_13_H_6_Cl_2_FN_4_O_2_ ([M − H]^−^), 338.9852; found, 338.9852.

Compound **5d** 4-amino-3,5-dichloro-6-(5-fluoro-2*H*-indazol-2-yl)-2-picolinic acid. White solid; 203.6–204.2 °C; ^1^H NMR (500 MHz, DMSO-*d*_6_) δ 13.62 (s, 1H), 8.24–8.15 (m, 1H), 7.60 (d, *J* = 8.0 Hz, 1H), 7.18 (dd, *J* = 11.4, 7.6 Hz, 1H), 7.09 (ddd, *J* = 7.8, 4.5, 4.4 Hz, 1H). ^13^C NMR (126 MHz, DMSO-*d*_6_) δ 165.84, 153.62, 151.61, 151.04, 146.97, 146.77, 140.21, 140.08, 127.33, 124.89, 124.84, 122.78, 122.73, 118.18, 118.15, 112.77, 110.78, 110.40, 110.27. HRMS calcd. For C_13_H_6_Cl_2_FN_4_O_2_ ([M − H]^−^), 338.9852; found, 338.9851.

Compound **6A** 4-amino-3,5-dichloro-6-(4-chloro-1*H*-indazol-1-yl)-2-picolinic acid. White solid; 195.3–195.8 °C; ^1^H NMR (500 MHz, DMSO-*d*_6_) δ 13.28 (s, 1H), 8.50 (s, 1H), 7.57 (d, *J* = 8.4 Hz, 1H), 7.49 (dd, *J* = 7.5, 0.9 Hz, 1H), 7.38 (d, *J* = 7.3 Hz, 2H), 7.38 (s, 1H). ^13^3C NMR (126 MHz, DMSO-*d*_6_) δ 165.99, 151.27, 146.85, 145.89, 140.99, 134.46, 129.09, 125.58, 123.34, 122.15, 112.08, 111.03, 110.87. HRMS calcd. For C_13_H_6_Cl_3_N_4_O_3_ ([M − H]^−^), 354.9556; found, 354.9559.

Compound **6a** 4-amino-3,5-dichloro-6-(4-chloro-2*H*-indazol-2-yl)-2-picolinic acid. White solid; 206.2–207.3 °C; ^1^H NMR (500 MHz, DMSO-*d*_6_) δ 13.98 (s, 1H), 8.93 (s, 1H), 7.73 (d, *J* = 8.8 Hz, 1H), 7.48 (s, 2H), 7.36 (dd, *J* = 8.7, 7.2 Hz, 1H), 7.25 (d, *J* = 7.1 Hz, 1H). ^13^C NMR (126 MHz, DMSO-*d*_6_) δ 165.78, 151.03, 149.42, 146.93, 146.84, 128.13, 125.62, 125.25, 122.11, 121.65, 117.34, 112.77, 110.74. HRMS calcd. For C_13_H_6_Cl_3_N_4_O_3_ ([M − H]^−^), 354.9556; found, 354.9532.

Compound **6B** 4-amino-3,5-dichloro-6-(5-chloro-1*H*-indazol-1-yl)-2-picolinic acid. White solid; 198.5–199.1 °C; ^1^H NMR (500 MHz, DMSO-*d*_6_) δ 13.17 (s, 1H), 8.41 (d, *J* = 0.7 Hz, 1H), 8.00 (d, *J* = 1.7 Hz, 1H), 7.64 (d, *J* = 8.9 Hz, 1H), 7.51 (dd, *J* = 8.9, 2.0 Hz, 1H), 7.36 (s, 2H). ^13^C NMR (126 MHz, DMSO-*d*_6_) δ 165.83, 151.05, 147.36, 146.92, 146.71, 128.59, 127.20, 126.18, 122.01, 120.45, 120.24, 112.67, 110.53. HRMS calcd. For C_13_H_6_Cl_3_N_4_O_3_ ([M − H]^−^), 354.9556; found, 354.9555.

Compound **6b** 4-amino-3,5-dichloro-6-(5-chloro-2*H*-indazol-2-yl)-2-picolinic acid. White solid; 204.2–205.3 °C; ^1^H NMR (500 MHz, DMSO-*d*_6_) δ 13.40 (s, 1H), 8.80 (s, 1H), 7.92 (d, *J* = 1.3 Hz, 2H), 7.78 (d, *J* = 9.2 Hz, 1H), 7.46 (s, 2H), 7.34 (dd, *J* = 9.2, 2.0 Hz, 1H). ^13^C NMR (126 MHz, DMSO-*d*_6_) δ 165.83, 151.05, 147.36, 146.94, 146.74, 128.58, 127.18, 126.20, 122.01, 120.46, 120.27, 112.63, 110.52. HRMS calcd. For C_13_H_6_Cl_3_N_4_O_3_ ([M − H]^−^), 354.9556; found, 354.9554.

Compound **6Cc** (mixture) 4-amino-3,5-dichloro-6-(6-chloro-1*H*-indazol-1-yl)-2-picolinic acid and 4-amino-3,5-dichloro-6-(6-chloro-2*H*-indazol-2-yl)-2-picolinic acid (0.48: 1). Yellow solid; 158.0–171.4 °C; ^1^H NMR (500 MHz, DMSO-*d*_6_) δ 13.27 (s, 1H), 8.91 (s, 1H), 7.92 (d, *J* = 9.0 Hz, 1H), 7.88 (s, 1H), 7.48 (s, 2H), 7.18 (dd, *J* = 8.9, 1.6 Hz, 1H). ^13^C NMR (126 MHz, DMSO-*d*_6_) δ 165.96, 151.34, 146.67, 145.92, 140.33, 133.40, 132.33, 123.38, 123.26, 123.21, 112.63, 111.85, 110.59; ^1^H NMR (500 MHz, DMSO-*d*_6_) δ 13.27 (s, 1H), 8.49 (s, 1H), 7.96 (d, *J* = 8.6 Hz, 1H), 7.79 (s, 1H), 7.37(s, 2H), 7.36 (dd, *J* = 8.5, 1.7 Hz, 1H). ^13^C NMR (126 MHz, DMSO-*d*_6_) δ 165.83, 151.05, 149.05, 147.00, 146.78, 136.73, 127.06, 123.87, 123.86, 120.21, 116.87, 111.82, 110.54. HRMS calcd. For C_13_H_6_Cl_3_N_4_O_2_ ([M − H]^−^), 338.9556; found, 338.9555.

Compound **6d** 4-amino-3,5-dichloro-6-(7-chloro-2*H*-indazol-2-yl)-2-picolinic acid. White solid; 211.5–212.1 °C; ^1^H NMR (500 MHz, DMSO-*d*_6_) δ 13.89 (s, 1H), 8.94 (s, 1H), 7.81 (d, *J* = 8.1 Hz, 1H), 7.48 (dd, *J* = 6.8, 0.4 Hz, 1H), 7.46 (s, 2H), 7.13 (dd, *J* = 8.4, 7.2 Hz, 1H). ^13^C NMR (126 MHz, DMSO-*d*_6_) δ 165.84, 151.02, 147.06, 146.82, 146.41, 127.88, 126.94, 123.24, 122.98, 122.28, 121.11, 112.79, 110.89. HRMS calcd. For C_13_H_6_Cl_3_N_4_O_3_ ([M − H]^−^), 354.9556; found, 354.9555.

Compound **7A** 4-amino-3,5-dichloro-6-(4-bromo-1*H*-indazol-1-yl)-2-picolinic acid. Yellow solid;181.5–182.3 °C; ^1^H NMR (500 MHz, DMSO-*d*_6_) δ 13.51 (s, 1H), 8.39 (s, 1H), 7.61 (d, *J* = 8.4 Hz, 1H), 7.53 (d, *J* = 7.4 Hz, 1H), 7.42 (dd, *J* = 7.8, 0.4 Hz, 1H), 7.37 (s, 2H). ^13^C NMR (126 MHz, DMSO-*d*_6_) δ 165.95, 151.29, 146.93, 145.95, 140.68, 135.85, 129.39, 125.36, 125.18, 113.85, 111.98, 111.53, 110.83. HRMS calcd. For C_13_H_6_BrCl_2_N_4_O_3_ ([M − H]^−^), 398.9051; found, 398.9055.

Compound **7a** 4-amino-3,5-dichloro-6-(4-bromo-2*H*-indazol-2-yl)-2-picolinic acid. Yellow solid; 206.4–207.2 °C; ^1^H NMR (500 MHz, DMSO-*d*_6_) δ 13.60 (s, 1H), 8.83 (s, 1H), 7.76 (d, *J* = 8.7 Hz, 1H), 7.45 (s, 2H), 7.41 (d, *J* = 7.1 Hz, 1H), 7.30 (dd, *J* = 8.6, 7.2 Hz, 1H). ^13^C NMR (126 MHz, DMSO-*d*_6_) δ 165.77, 151.03, 148.90, 146.92, 146.69, 128.56, 126.92, 125.51, 123.40, 117.74, 113.30, 112.79, 110.76. HRMS calcd. For C_13_H_6_BrCl_2_N_4_O_3_ ([M − H]^−^), 398.9051; found, 398.9054.

Compound **7B** 4-amino-3,5-dichloro-6-(5-bromo-1*H*-indazol-1-yl)-2-picolinic acid. Yellow solid; 207.5–208.6 °C; ^1^H NMR (500 MHz, DMSO-*d*_6_) δ 13.45 (s, 1H), 8.41 (s, 1H), 8.15 (s, 1H), 7.66–7.57 (m, 2H), 7.35 (s, 2H). ^13^C NMR (126 MHz, DMSO-*d*_6_) δ 165.98, 151.29, 146.81, 145.97, 138.76, 135.95, 130.68, 126.27, 124.03, 114.75, 114.04, 111.76, 110.43. HRMS calcd. For C_13_H_6_BrCl_2_N_4_O_3_ ([M − H]^−^), 398.9051; found, 398.9056.

Compound **7b** 4-amino-3,5-dichloro-6-(5-bromo-2*H*-indazol-2-yl)-2-picolinic acid. Yellow solid; 209.9–210.2 °C; ^1^H NMR (500 MHz, DMSO-*d*_6_) δ 13.39 (s, 1H), 8.79 (s, 1H), 8.08 (s, 1H), 7.71 (d, *J* = 9.2 Hz, 1H), 7.44–7.42 (m, 3H). ^13^C NMR (126 MHz, DMSO-*d*_6_) δ 165.81, 151.06, 147.39, 146.95, 146.77, 130.78, 126.02, 123.85, 122.87, 120.48, 115.36, 112.60, 110.52. HRMS calcd. For C_13_H_6_BrCl_2_N_4_O_3_ ([M − H]^−^), 398.9051; found, 398.9053.

Compound **7Cc** (mixture) 4-amino-3,5-dichloro-6-(6-bromo-1*H*-indazol-1-yl)-2-picolinic acid and 4-amino-3,5-dichloro-6-(6-bromo-2*H*-indazol-2-yl)-2-picolinic acid (1: 1.03). Yellow solid; 143.7–158.9 °C; ^1^H NMR (500 MHz, DMSO-*d*_6_) δ 13.73 (s, 1H), 8.86 (s, 1H), 8.01 (s, 1H), 7.86 (d, *J* = 8.5 Hz, 1H), 7.43 (s, 2H), 7.43 (dd, *J* = 8.5, 1.5 Hz, 1H). ^13^C NMR (126 MHz, DMSO-*d*_6_) δ 165.83, 149.59, 146.99, 146.80. 146.78, 140.68, 127.12, 123.60, 120.90, 120.32, 112.62, 110.58; ^1^H NMR (500 MHz, DMSO-*d*_6_) δ 13.11 (s, 1H), 8.44 (s, 1H), 7.90 (s, 1H), 7.82 (d, *J* = 8.9 Hz, 1H), 7.33 (s, 2H), 7.25 (d, *J* = 9.0 Hz, 1H). ^13^C NMR (126 MHz, DMSO-*d*_6_) δ 165.96, 151.34, 151.05, 146.68, 145.91, 136.78, 126.14, 125.73, 123.98, 123.46, 121.53, 120.19, 114.80, 111.85. HRMS calcd. For C_13_H_6_BrCl_2_N_4_O_3_ ([M − H]^−^), 398.9051; found, 398.9050.

Compound **7d** 4-amino-3,5-dichloro-6-(7-bromo-2*H*-indazol-2-yl)-2-picolinic acid. Yellow solid; 212.3–213.2 °C; ^1^H NMR (500 MHz, DMSO-*d*_6_) δ 13.21 (s, 1H), 8.97 (s, 1H), 7.85 (d, *J* = 8.4 Hz, 1H), 7.65 (d, *J* = 7.1 Hz, 1H), 7.46 (s, 2H), 7.07 (dd, *J* = 7.0, 1.0 Hz, 1H). ^13^C NMR (126 MHz, DMSO-*d*_6_) δ 165.85, 151.01, 147.47, 147.07, 146.83, 130.35, 128.01, 123.67, 122.52, 121.63, 112.78, 110.99, 110.92. HRMS calcd. For C_13_H_6_BrCl_2_N_4_O_3_ ([M − H]^−^), 398.9051; found, 398.9053.

### 3.3. Determination of the Biological Activities

#### 3.3.1. Phenotypic Study for the Inhibition of *A. thaliana* Root Growth

Seeds of *Col-0* were surface-sterilized using 1% sodium hypochlorite solution, and were sown onto 1/2 Modified Medium (with vitamins, sucrose and phytagel) with compounds at 200, 100, 50, 25, 12, 6 and 3 µM in Petri dishes. Subsequently, seeds were incubated at 4 °C for two days under darkness, and were cultured on vertically oriented Petri dishes at 22 °C for 7 d under light/dark (16 h/8 h) cycling in a plant incubator. The taproot length of 7-day-old seedlings was measured using IMAGEJ software. The inhibition percentage of *A. thaliana* root growth was calculated based on the following equation:

$$P_{0}=\frac{L_{a0}-L_{a}}{L_{a0}}\times 100\%$$

where *P*_0_ is the inhibition percentage, and *L*_a_ and *L*_a0_ are the average lengths of the roots if *A. thaliana* in the presence of compounds and untreated controls, respectively.

#### 3.3.2. Evaluation of the Herbicidal Activity

The herbicidal activity of synthetic compounds was evaluated according to a reported procedure, and picloram was used as the positive control and the results represent the bioactivity triplicate [18]. Preliminary herbicidal activities of synthetic compounds against BN, AM, EC, AR and CA were screened at concentrations of 500 and 250 μM in Petri dish tests. Emulsions of new compounds and picloram were prepared by dissolving them in the mixture of DMSO (0.1 mL) and Tween-80 (0.1 mL) followed by dispersing in deionized water (10 mL). A mixture of the same amount of water, DMSO and Tween-80 was used as the untreated control. The seeds were soaked in warm water (25 °C) for 15 h before use, and about 10 seeds of BN, AM, AR, CA and EC were placed onto germinating paper (6 cm) wetted with 5 mL emulsions in a 6 cm Petri dish. The plates were placed in a dark room and allowed to germinate for 36 h at 25 ± 1 °C, then transferred to 22 °C for four to seven days under light/dark (16 h/8 h) cycling. The lengths of five BN, AM, AR, CA and EC root radicles randomly selected from each plate were measured, respectively. The average of the root lengths was calculated, and the inhibition percentage was calculated using the following equation:

$$P_{1}=\frac{L_{w0}-L_{w}}{L_{w0}}\times 100\%$$

where *P*_1_ is the inhibition percentage, and *L*_w_ and *L*_w0_ are the average lengths of the five weed’s roots in the presence of compounds and untreated controls, respectively.

Furthermore, the vivo post-emergence herbicidal activity of new compounds against four dicotyledonous weeds, BN, AM, AR and CA, and one monocotyledonous weed, EC, was tested at a dosage of 1000, 500 and 250 g ha^−1^ in a glasshouse (Xian Zheng Fei Greenhouse, Science and Technology Park, China Agricultural University, Beijing, China). The plant growth medium was obtained by mixing peat soil, flower soil and vermiculite at a mass ratio of 1:3:2. Preparation of the emulsions of new compounds and picloram was the same for tests in Petri dishes. The emulsions were sprayed using a spray bottle at a dosage of 1000, 500 and 250 g ha^−1^ after the plants reached the two-leaf stage. Subsequently, the seedings grew in the greenhouse (natural environment, no additional lighting, 25–35 °C). Weed growth and toxic symptoms were observed regularly after treatment and the growth inhibitory activities of each compound were visually evaluated 14 d after treatment based on the following index: all dead: 100%; stems atrophy and dead leaves: 80%; stems atrophy and partially dead leaves: 60%, normal stems and partially dead leaves: 40%; normal stems and partially curled leaves: 20%, and normal stems and leaves = 0.

### 3.4. Quantitative Real-Time PCR

*A. thaliana* was cultured on 1/2 Modified Medium (with vitamins, sucrose, and phytagel) in Petri dishes (10 × 10 cm), and placed in a plant incubator after the seeds were incubated at 4 °C for two days under darkness. The seeds were cultured on vertically oriented Petri dishes at 22 °C for two weeks under light/dark (16 h/8 h) cycling. Seedlings then were carefully placed on filter paper wetted with the solution of compound **7Cc**, **5A**, **5a**, picloram at a concentration of 100 μM and deionized water in a Petri dish (10 × 10 cm), respectively. After 12 h, 24 h and 72 h, the whole seedlings were rapid frozen with liquid nitrogen, collected and stored at −80 °C. Detailed steps are described in the Supporting Information.

## 4. Conclusions

In this study, based on scaffold hopping, 38 4-amino-3,5-dicholor-6-(1*H*-indazol-1-yl)-2-picolinic acid and 4-amino-3,5-dicholor-6-(2*H*-indazol-1-yl)-2-picolinic acid compounds were designed and synthesized via a four-step synthetic route with good yields. The results of primary bioassay on the root growth inhibition of *A. thaliana* demonstrated that most compounds had an excellent inhibitory effect, especially with substituents at the 4, 6, 7 position and electron-withdrawing groups on the indazole ring. Compound **5a** had a comparable performance against the commercial herbicide picloram, while **7Cc** was superior to picloram. The herbicidal activity in Petri dishes showed that most of the compounds were able to inhibit root growth in five weeds, while compound **5a** showed significantly better activity than picloram in the root inhibition assay of BN and AM at a concentration of 10 µM. Most of the compounds exhibited excellent herbicidal activity in post-emergence at 1000 and 500 g/ha, while compound **5a** also had an injurious effect on AR and CA at 250 g/ha. Overall, 6-indazolyl-2-picolinic acids with an electron-withdrawing substituent on the indazole ring and substituent at the 4 position exhibited excellent root inhibitory and herbicidal activities. Compounds **7Cc**, **5A**, and **5a** did not arouse the up-regulation of auxin-related gene as picloram did, but they promoted ethylene release and ABA production through the up-regulation of ACS and NCED genes to cause plant death in a short period of time. These results provide new perspectives and insights for the future design of compounds with similar structures.

## Figures and Tables

**Table 1:** The structures of new compounds.

No.	Compd.	R	No.	Compd.	R
**1A**	**IV**	H	**3c**	**V**	6-NH_2_
**2A**	**IV**	4-CH_3_	**3d**	**V**	7-NH_2_
**2B**	**IV**	5-CH_3_	**4a**	**V**	4-OCH_3_
**3A**	**IV**	4-NH_2_	**4b**	**V**	5-OCH_3_
**3C**	**IV**	6-NH_2_	**4c**	**V**	6-OCH_3_
**4A**	**IV**	4-OCH_3_	**4d**	**V**	7-OCH_3_
**4B**	**IV**	5-OCH_3_	**5a**	**V**	4-F
**4C**	**IV**	6-OCH_3_	**5b**	**V**	5-F
**5A**	**IV**	4-F	**5d**	**V**	7-F
**5B**	**IV**	5-F	**6a**	**V**	4-Cl
**6A**	**IV**	4-Cl	**6b**	**V**	5-Cl
**6B**	**IV**	5-Cl	**6d**	**V**	7-Cl
**7A**	**IV**	4-Br	**7a**	**V**	4-Br
**7B**	**IV**	5-Br	**7b**	**V**	5-Br
**1a**	**V**	H	**7d**	**V**	7-Br
**2a**	**V**	4-CH_3_	**2Cc**	**IV+V**	6-CH_3_
**2b**	**V**	5-CH_3_	**5Cc**	**IV+V**	6-F
**2d**	**V**	7-CH_3_	**6Cc**	**IV+V**	6-Cl
**3a**	**V**	4-NH_2_	**7Cc**	**IV+V**	6-Br

**Table 2:** Reaction condition optimization for increasing the percentage of r_1_ in the product.

Solvent	Base	Temperature (T_1_) ^a^	Temperature (T_2_) ^b^	Reaction Progress Conversion/Ratio of r_1_:r_2_
Extra dry dioxane	NaH	50 °C	100 °C	Complete/r_1_:r_2_ = 1:1
Extra dry dioxane	NaH	25 °C	100 °C	Incomplete (5%) ^c^/r_1_:r_2_ = 1:1
Extra dry dioxane	K_2_CO_3_	50 °C	100 °C	No reaction
Extra dry dioxane	KOH	50 °C	100 °C	No reaction
Extra dry dioxane	NaOH	50 °C	100 °C	No reaction
Extra dry dioxane	CsCO_3_	50 °C	100 °C	Incomplete (10%) ^c^/r_1_:r_2_ = 2.5:1
Extra dry dioxane	CsCO_3_	25 °C	100 °C	Incomplete (15%) ^c^/r_1_:r_2_ = 3.4:1
Extra dry dioxane	CsCO_3_	25 °C	50 °C	Incomplete (20%) ^c^/r_1_:r_2_ = 1.3:1
Acetonitrile	CsCO_3_	−13 °C	80 °C	Incomplete (25%) ^c^/r_1_:r_2_ = 3.9:1
1,2-Dimethoxyethane	CsCO_3_	−10 °C	85 °C	Incomplete (15%) ^c^/r_1_:r_2_ = 4.9:1
1,2-Diethoxyethane	CsCO_3_	−10 °C	110 °C	Incomplete (5%) ^c^/r_1_:r_2_ = 2.25:1

^a^ 3-h reaction time at T_1_ temperature. ^b^ 12-h reaction time at T_2_ temperature. ^c^ Percentage of unreacted 4-amino-3,5,6-trichloropicolinonitrile.

**Table 3:** Herbicidal activities of some compounds against five weeds.

Compound	Dosage (g ha^−1^)	BN (%)	AM (%)	CA (%)	AR (%)	EC (%)
**1a**	1000	100	30	100	100	0
	500	100	/	100	100	0
	250	100	/	15	100	0
**2b**	1000	100	100	100	100	0
	500	100	45	70	100	0
	250	100	/	45	100	0
**2d**	1000	100	100	100	100	0
	500	80	40	100	100	0
	250	60	/	100	100	0
**3d**	1000	100	0	100	100	0
	500	100	/	100	100	0
	250	100	/	30	80	0
**4A**	1000	100	100	100	100	0
	500	100	0	70	100	0
	250	100	/	10	100	0
**4d**	1000	100	80	100	100	0
	500	100	70	65	100	0
	250	100	10	30	95	0
**5A**	1000	100	100	100	100	0
	500	100	5	100	100	0
	250	100	/	75	100	0
**5a**	1000	100	100	100	100	0
	500	65	10	100	100	0
	250	15	/	40	75	0
**6A**	1000	100	85	100	100	0
	500	100	10	100	100	0
	250	100	/	45	85	0
**6a**	1000	100	80	100	100	0
	500	20	5	100	100	0
	250	/	/	45	90	0
**6b**	1000	100	10	80	100	0
	500	100	/	60	100	0
	250	100	/	10	100	0
**6d**	1000	100	70	100	100	0
	500	50	25	100	100	0
	250	/	/	65	90	0
**7a**	1000	100	70	100	100	0
	500	100	0	100	100	0
	250	100	/	50	80	0
**7d**	1000	100	75	100	100	0
	500	40	5	100	100	0
	250	/	/	30	85	0
Picloram	1000	100	100	100	100	10
	500	100	65	100	100	0
	250	100	40	80	85	0

## Data Availability

Data are contained within the article and Supplementary Materials.

## Supplementary Materials

The following supporting information can be downloaded at: https://www.mdpi.com/article/10.3390/molecules29020332/s1, Figure S1: The root inhibition of compounds on EC at 500 µM and 250 µM; Figure S2: The root inhibition of compounds on AR at 500 µM and 250 µM; Figure S3: The root inhibition of compounds on CA at 500 µM and 250 µM; Figure S4: The root inhibit ion of compounds on AM at 500 µM and 250 µM; Figure S5: The root inhibition of compounds on BN at 500 µM and 250 µM; Figure S6: Summary of the visual injury percentages resulting from treatment of the five dicotyledonous weeds 14 days with the compounds at concentrations 1000 g/ha; Table S1: Herbicidal activities of some compounds against five weeds (reflected in terms of the visual injury effect %); Table S2: Primers for real-time PCR used in this study; Additional Experimental Details for qPCR; The^1^H and ^13^C NMR spectra of compounds **1A**–**7d**.

## References

[1] Li L. Analysis of Global Pesticide Market Overview, Market Capacity, Product Structure and Market Competition Pattern in 2017. http://www.chyxx.com/industry/201707/539506.html.

[2] Oliveira M.C., Osipitanc O.A., Begcyd K., Werle R. (2020). Cover crops, hormones and herbicides: Priming an integrated weed management strategy. Plant Sci..

[3] Bagavathiannan M.V., Graham S., Ma Z., Barney J.N., Coutts S.R., Caicedo A.L., De Clerck-Floate R., West N.M., Blank L., Metcalf A.L. (2019). Considering weed management as a social dilemma bridges individual and collective interests. Nat. Plants.

[4] Barratt B.I.P., Moran V.C., Bigler F. (2017). The status of biological control and recommendations for improving uptake for the future. BioControl.

[5] Heap I. International Survey of Herbicide Resistant Weeds. http://weedscience.org.

[6] Busi R., Goggin D.E., Heap I.M., Horak M.J., Jugulam M., Masters R.A., Napier R.M., Riar D.S., Satchivi N.M., Torra J. (2018). Weed resistance to synthetic auxin herbicides. Pest Manag. Sci..

[7] Eerd L.L.V., Mclean M.D., Stephenson G.R., Hall J.C. (2004). Resistance to quinclorac and ALS-inhibitor herbicides in Galium spurium is conferred by two distinct genes. Weed Res..

[8] Heap I. (2014). Global perspective of herbicide-resistant weeds. Pest Manag. Sci..

[9] Walsh T.A., Neal R., Merlo A.O., Honma M., Hicks G.R., Wolff K., Matsumura W., Davies J.P. (2006). Mutations in an auxin receptor homolog AFB5 and in SGT1b confer resistance to synthetic picolinate auxins and not to 2,4-dichlorophenoxyacetic acid or indole-3-acetic acid in Arabidopsis. Plant Physiol..

[10] Walsh M.J., Maguire N., Powles S.B. (2008). Combined effects of wheat competition and 2,4-D amine on phenoxy herbicide resistant Raphanus raphanistrum populations. Weed Res..

[11] Schmitzer P.R., Balko T.W., Daeuble J.F., Epp J.B., Satchivi N.M., Siddall T.L., Weimer M.R., Yerkes C.N. (2015). Discovery and SAR of Halauxifen Methyl: A Novel Auxin Herbicide. Discovery and Synthesis of Crop Protection Products.

[12] Epp J.B., Alexander A.L., Balko T.W., Buysse A.M., Brewster W.K., Bryan K., Daeuble J.F., Fields S.C., Gast R.E., Green R.A. (2016). The discovery of Arylex active and Rinskor active: Two novel auxin herbicides. Bioorganic. Med. Chem..

[13] Walsh T., Schmitzer P., Masters R.A., Lo W.C., Gast R.E., Claus J.S., Finkelstein B.L. (2019). New Auxin Mimics and Herbicides. Modern Crop Protection Compounds.

[14] Schmitzer P., Epp J., Gast R., Lo W., Nelson J. (2016). Herbicidal Carboxylic Acids as Synthetic Auxins. Bioactive Carboxylic Compound Classes.

[15] Hoffmann M.G., Brünjes M., Döller U., Dietrich H. (2015). Substituted Picolinic Acid and Pyrimidine-4-carboxylic Acids, Method for the Production Thereof, and Use Thereof as Herbicides and Plant Growth Regulators.

[16] Eckelbarger J.D., Epp J.B., Fischer L.G., Lowe C.T., Petkus J., Roth J., Satchivi N.M., Schmitzer P.R., Siddall T.L. (2014). 4-Amino-6-(heterocyclic)picolinates and 6-Amino-2-(heterocyclic)pyrimidine-4-carboxylates and Their Use as Herbicides.

[17] Ko Y.K., Kim E.A., Lee I.Y., Koo D.W., Ryu J.W., Yon G.H. (2018). Pyridine-Based Compound Including Isoxazoline ring, and Use Thereof as Herbicide.

[18] Yang Z., Li Q., Yin J., Liu R., Tian H., Duan L., Li Z., Wang B., Tan W., Liu S. (2021). Design, synthesis and mode of action of novel 3-chloro-6-pyrazolyl picolinate derivatives as herbicide candidates. Pest Manag. Sci..

[19] Feng T., Liu Q., Xu Z.Y., Li H.T., Wei W., Shi R.C., Zhang L., Cao Y.M., Liu S.Z. (2023). Design, Synthesis, Herbicidal Activity, and Structure-Activity Relationship Study of Novel 6-(5-Aryl-Substituted-1-Pyrazolyl)-2-Picolinic Acid as Potential Herbicides. Molecules.

[20] Cerecetto H., Gerpea A., González M., Aránb V.J., Ocáriz C.O.d. (2005). Pharmacological Properties of Indazole Derivatives: Recent Developments. Mini-Rev. Med. Chem..

[21] Gaikwad D.D., Chapolikar A.D., Devkate C.G., Warad K.D., Tayade A.P., Pawar R.P., Domb A.J. (2015). Synthesis of indazole motifs and their medicinal importance: An overview. Eur. J. Med. Chem..

[22] James D.R., Baker D.R., Mielich S.D., Michaely W.J., Fitzjohn S., Knudsen C.G., Mathews C., Gerdes J.M. (1993). Preparation of Arylindazoles and Their Use as Herbicides.

[23] Hwang I.T., Kim H.R., Jeon D.J., Hong K.S., Song J.H., Chung C.K., Cho K.Y. (2005). New 2-phenyl-4,5,6,7-tetrahydro-2H-indazole derivatives as paddy field herbicides. Pest Manag. Sci..

[24] Jayachandran D., Amaruka H., Christopher K., Melissa L., Fangzheng L. (2021). Improved Synthesis of 4-Amino-6-(heterocyclic)picolinates.

[25] Hansen H., Grossmann K. (2000). Auxin-Induced Ethylene Triggers Abscisic Acid Biosynthesis and Growth Inhibition. Plant Physiol..

[26] Xu J., Liu X., Napier R., Dong L., Li J. (2022). Mode of Action of a Novel Synthetic Auxin Herbicide Halauxifen-Methyl. Agronomy.

[27] Lei K., Li P., Yang X.F., Wang S.B., Wang X.K., Hua X.W., Sun B., Ji L.S., Xu X.H. (2019). Design and Synthesis of Novel 4-Hydroxyl-3-(2-phenoxyacetyl)-pyran-2-one Derivatives for Use as Herbicides and Evaluation of Their Mode of Action. J. Agric. Food Chem..

[28] Yang F., Song Y., Yang H., Liu Z., Zhu G., Yang Y. (2014). An auxin-responsive endogenous peptide regulates root development in Arabidopsis. J. Integr. Plant Biol..

[29] Dharmasiri S., Swarup R., Mockaitis K., Dharmasiri N., Singh S.K., Kowalchyk M., Marchant A., Mills S., Sandberg G., Bennett M.J. (2006). AXR4 Is Required for Localization of the Auxin Influx Facilitator AUX1. Science.

[30] Dezfulian M.H., Jalili E., Roberto D.K., Moss B.L., Khoo K., Nemhauser J.L., Crosby W.L. (2016). Oligomerization of SCFTIR1 Is Essential for Aux/IAA Degradation and Auxin Signaling in Arabidopsis. PLoS Genet..

[31] Kelley K.B., Riechers D.E. (2007). Recent developments in auxin biology and new opportunities for auxinic herbicide research. Pestic. Biochem. Physiol..

[32] Chen X., Zhou S., Qian C., He C. (2012). Synthesis of 1H-indazole: A combination of experimental and theoretical studies. Res. Chem. Intermed..

[33] Pan J., Fujioka S., Peng J., Chen J., Li G., Chen R. (2009). The E3 ubiquitin ligase SCFTIR1/AFB and membrane sterols play key roles in auxin regulation of endocytosis, recycling, and plasma membrane accumulation of the auxin efflux transporter PIN2 in Arabidopsis thaliana. Plant Cell.

[34] Tan X., Calderon-Villalobos L.I., Sharon M., Zheng C., Robinson C.V., Estelle M., Zheng N. (2007). Mechanism of auxin perception by the TIR1 ubiquitin ligase. Nature.

[35] Calderon Villalobos L.I., Lee S., De Oliveira C., Ivetac A., Brandt W., Armitage L., Sheard L.B., Tan X., Parry G., Mao H. (2012). A combinatorial TIR1/AFB-Aux/IAA co-receptor system for differential sensing of auxin. Nat. Chem. Biol..

