# Supplementary material for: Study on Design, Synthesis and Herbicidal Activity of Novel 6-Indazolyl-2-picolinic Acids
Source: Molecules. 2024 Jan 9;29(2):332. doi: 10.3390/molecules29020332 (PMC10819873; doi:10.3390/molecules29020332)
Supplement: Supplementary file 1 [file molecules-29-00332-s001.zip › molecules-2795524-supplementary.pdf]

Additional Experimental Details for qPCR

The total RNA was extracted from the leaves using an RNAprep Pure Plant Kit (TIANGEN BIOTECH, Beijing, China) according to the manufacturer's instructions. The yield of RNA was determined using a Nanodrop 2000 spectrophotometer (Thermo Fisher Scientific, Waltham, MA, USA), and the integrity was evaluated using agarose gel electrophoresis and ethidium bromide staining. Quantification was performed using reverse transcription (RT) combined with the PCR. All relative solvent were provided by TIANGEN BIOTECH (Beijing, China). Each RT reaction consisted of two steps. In the first step, DNA was removed from the genome by adding a solution of RNA (1 μg), 5×DNA wiper mix (2 μL), and gDNA Eraser (1 μL) to nuclease-free H_2O (10 μL). The RT reaction was then performed using an ABI7500 RT-PCR instrument (Applied Biosystems, Foster City, CA, USA) for 2 min at 42 °C. In the second step, PrimeScript RT Enzyme Mix Ⅰ (1 μL), RT Primer Mix (1 μL), and 5×PrimeScript Buffer 2 (4 μL) were added to the mixture obtained from step 1 (10 μL), and nuclease-free H_2O (4 μL) was added. The reaction was then performed using an ABI7500 RT-PCR instrument (Applied Biosystems) for 15 min at 37 °C, then for 5 s at 85 °C. Subsequently, RT-PCR was performed using the same ABI7500 instrument. The PCR reaction mixture (20 μL) contained cDNA (2 μL), 2×Master Mix (10 μL), forward primer (0.5 μL), reverse primer (0.5 μL), and nuclease-free water (7 μL). Each reaction mixture was incubated in a 96-well optical plate (Roche) at 95 °C for 30 s, followed by 40 cycles of 95 °C for 5 s and 60 °C for 40 s. Each sample was run in triplicate. After completion of the PCR cycles, melting curve analysis was performed to validate the specific generation of the expected PCR product. The primer sequences were designed in the laboratory and synthesized by Tsingke Biotech (Shanghai, China) based on the mRNA sequences obtained from the NCBI database (see Table S2). The mRNA expression levels were normalized to the ACTIN-2 gene and calculated using the $2^{-\Delta\Delta Ct}$ method.

Table S1. Herbicidal activities of some compounds against five weeds (reflected in terms of the visual injury effect %)

Compound	Dosage (g ha^{-1})	BN (%)	AM (%)	CA (%)	AR (%)	EC (%)
1A	1000	100	100	100	65	0
	500	100	15	10	10	0
	250	100	/	/	/	0
1a	1000	100	30	100	100	0
	500	100	/	100	100	0
	250	100	/	15	100	0
2A	1000	70	100	100	100	0
	500	60	15	100	10	0
	250	5	/	100	/	0
2a	1000	100	100	100	100	0
	500	100	30	15	100	0
	250	100	/	/	100	0

2B	1000	100	40	100	100	0
	500	100	/	0	100	0
	250	100	/	/	70	0
2b	1000	100	100	100	100	0
	500	100	45	70	100	0
	250	100	/	45	100	0
2Cc	1000	100	100	100	100	0
	500	100	10	80	100	0
	250	100	/	20	85	0
2d	1000	100	100	100	100	0
	500	80	40	100	100	0
	250	60	/	100	100	0
3A	1000	100	10	80	100	0
	500	85	/	0	15	0
	250	65	/	/	/	0
3a	1000	100	20	85	100	0
	500	55	10	60	45	0
	250	/	/	45	/	0
3C	1000	100	100	50	100	0
	500	100	0	10	10	0
	250	100	/	/	/	0
3c	1000	100	100	100	100	0
	500	50	5	5	15	0
	250	/	/	/	/	0
3d	1000	100	0	100	100	0
	500	100	/	100	100	0
	250	100	/	30	80	0
4A	1000	100	100	100	100	0
	500	100	0	70	100	0
	250	100	/	10	100	0
4a	1000	100	100	100	100	0
	500	100	80	70	100	0
	250	100	15	25	75	0
4B	1000	100	100	25	100	0
	500	100	70	/	100	0
	250	100	5	/	100	0
4b	1000	100	15	100	100	0
	500	100	/	60	60	0
	250	100	/	15	55	0
4C	1000	100	15	10	15	0
	500	100	/	/	/	0
	250	100	/	/	/	0
4c	1000	100	0	100	100	0

	500	100	/	5	15	0
	250	100	/	/	/	0
4d	1000	100	80	100	100	0
	500	100	70	65	100	0
	250	100	10	30	95	0
5A	1000	100	100	100	100	0
	500	100	5	100	100	0
	250	100	/	75	100	0
5a	1000	100	100	100	100	0
	500	65	10	100	100	0
	250	15	/	40	75	0
5B	1000	100	100	100	100	0
	500	100	20	25	100	0
	250	100	/	/	100	0
5b	1000	100	100	100	100	0
	500	100	35	0	100	0
	250	100	/	/	75	0
5Cc	1000	100	100	100	100	0
	500	100	15	10	100	0
	250	100	/	/	65	0
5d	1000	85	80	100	100	0
	500	60	70	100	85	0
	250	5	0	35	75	0
6A	1000	100	85	100	100	0
	500	100	10	100	100	0
	250	100	/	45	85	0
6a	1000	100	80	100	100	0
	500	20	5	100	100	0
	250	/	/	45	90	0
6B	1000	100	100	75	80	0
	500	100	15	5	35	0
	250	100	/	/	/	0
6b	1000	100	10	80	100	0
	500	100	/	60	100	0
	250	100	/	10	100	0
6Cc	1000	100	100	5	100	0
	500	100	20	/	25	0
	250	100	/	/	/	0
6d	1000	100	70	100	100	0
	500	50	25	100	100	0
	250	/	/	35	90	0
7A	1000	100	100	100	55	0
	500	100	10	100	/	0

	250	100	/	40	/	0
7a	1000	100	70	100	100	0
	500	100	0	100	100	0
	250	100	/	50	80	0
7B	1000	100	80	10	5	0
	500	50	0	/	/	0
	250	/	/	/	/	0
7b	1000	100	40	100	100	0
	500	100	/	25	35	0
	250	100	/	/	/	0
7Cc	1000	100	100	100	100	0
	500	100	35	5	100	0
	250	100	/	/	65	0
7d	1000	100	75	100	100	0
	500	40	5	100	100	0
	250	/	/	30	85	0
Picloram	1000	100	100	100	100	10
	500	100	65	100	100	0
	250	100	40	80	85	0

Table S2. Primers for real-time PCR used in this study

**Primer names**	**sequence (5'-3')**
AUX1-F	GAAGGAGTAGAAGCGATAGT
AUX1-R	CCGTGCCATAGGAAAT
GH3.3-F	CAAACCAATCCTCCAAATGAC
GH3.3-R	ACTTATCCGCAACCCGACT
ARF2-F	AAACGTGCCGTCTTCTGTTATA
ARF2-R	GCTCGTCCTGGGTTTGTAGT
PIN2-F	GTTATCCTCGCCGCACTCTT
PIN2-R	CTTAGCCCCACGGAACTCAA
ACS7-F	TCCGGTGTCATCCAAATGGG

ACS7-R	AAAGTGTGACGTACCCTGGA
NCED3-F	TCGACCTCTTGTGTTGGTGG
NCED3-R	AGAGGGAACTCTTTTTGCTTCT
ACTIN2-F	GGTAACATTGTGCTCAGTGGTGG
ACTIN2-R	AACGACCTTAATCTTCATGCTGC

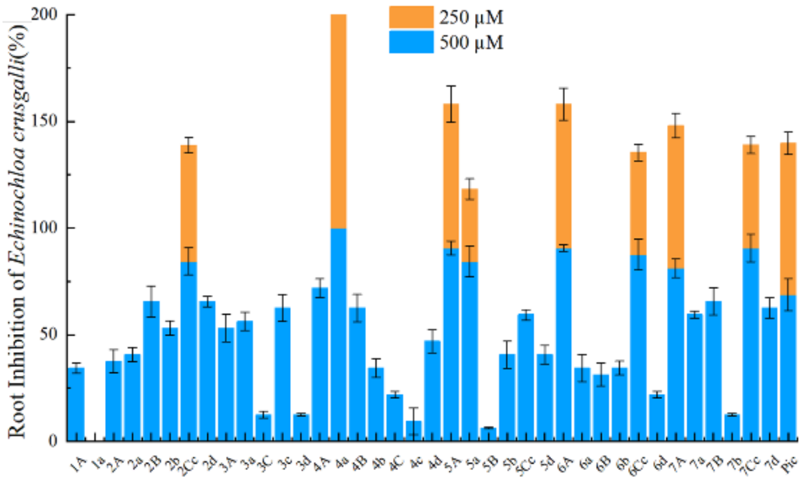

Figure S1. The root inhibition of compounds on EC at 500 μM and 250 μM.

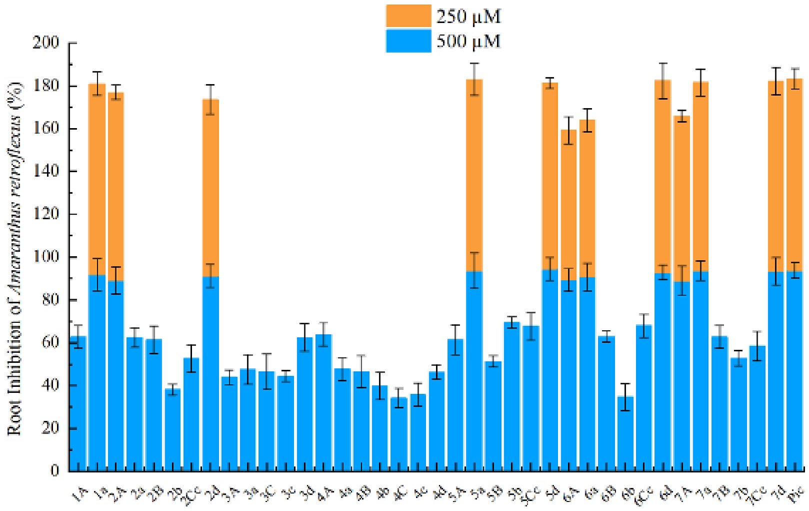

Figure S2. The root inhibition of compounds on AR at 500 μM and 250 μM.

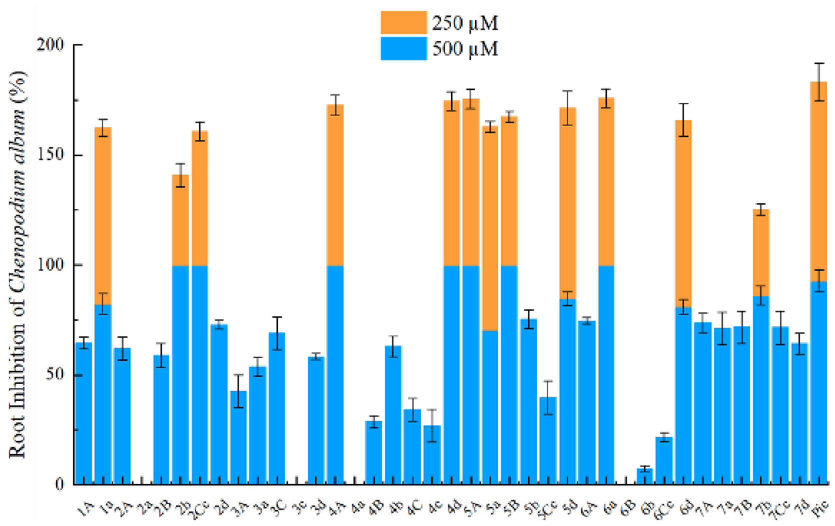

Figure S3. The root inhibition of compounds on CA at 500 μM and 250 μM.

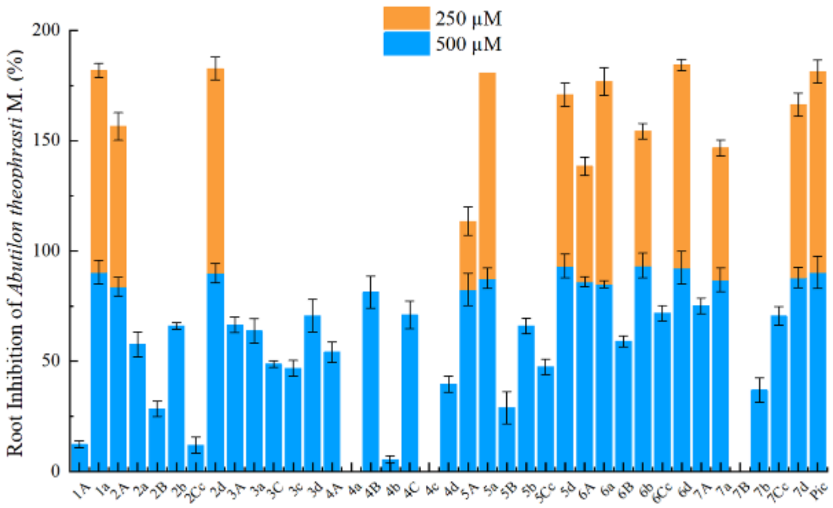

Figure S4. The root inhibition of compounds on AM at 500 μM and 250 μM.

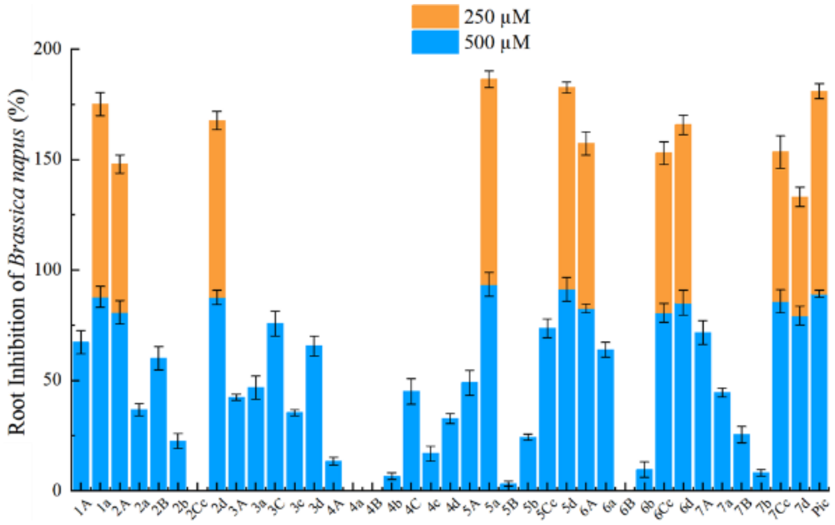

Figure S5. The root inhibition of compounds on BN at 500 μM and 250 μM.

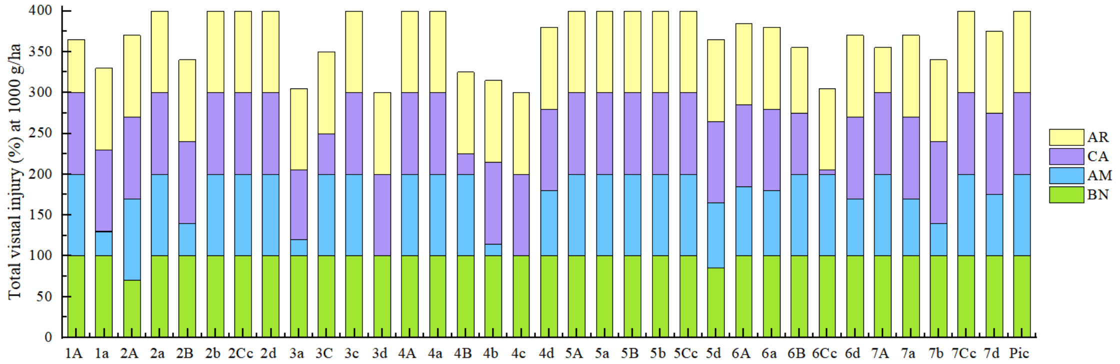

Figure S6. Summary of the visual injury percentages resulting from treatment of the five dicotyledonous weeds 14 days with the compounds at concentrations 1000 g/ha.

The ¹H NMR and ¹³C NMR Spectrum of Target Compounds

Compound 1A

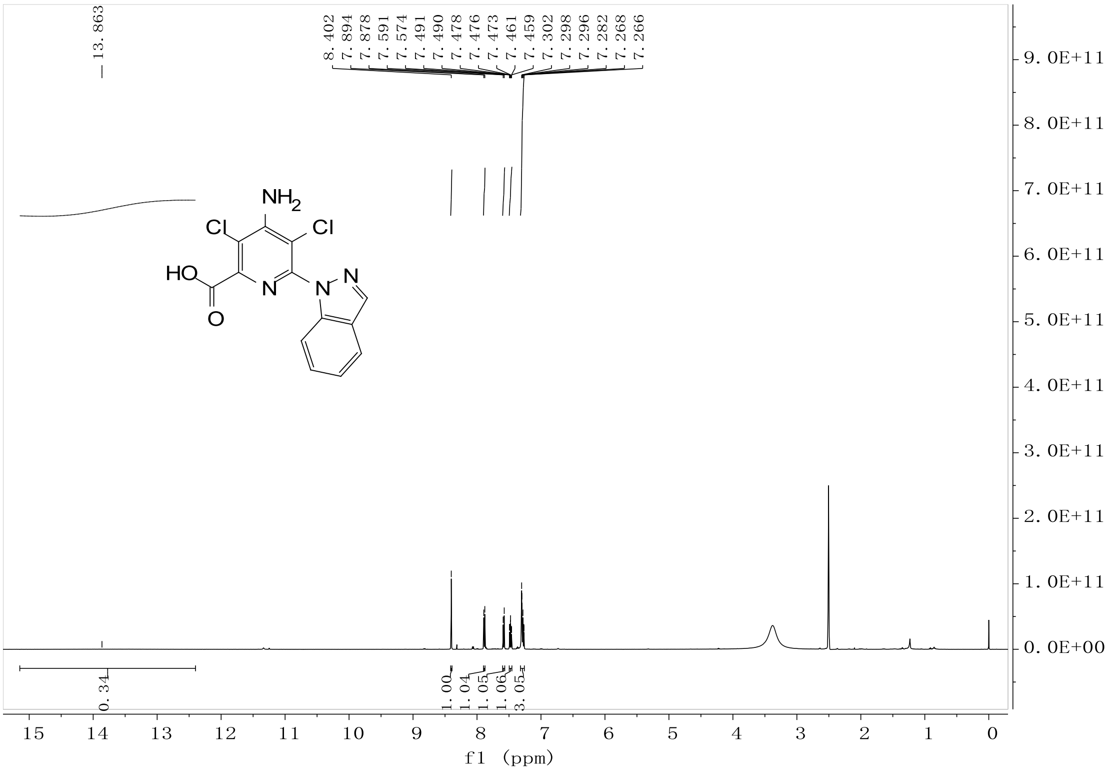

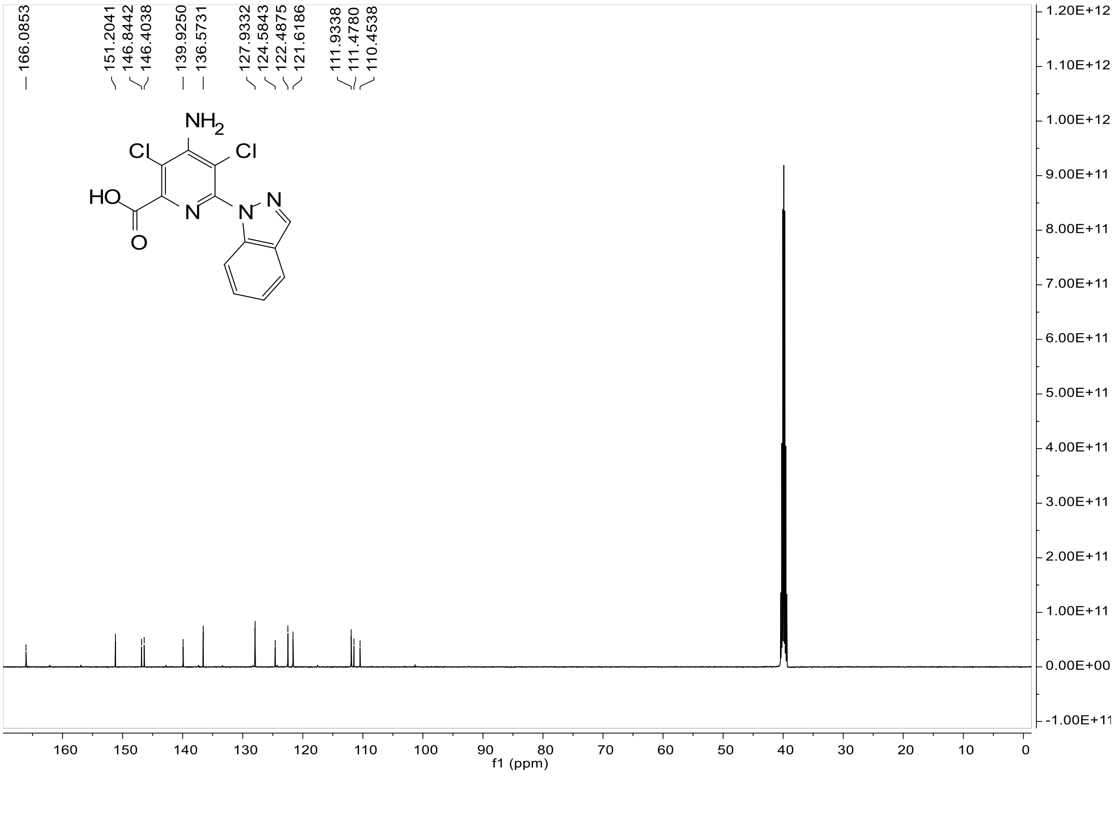

Compound 1a

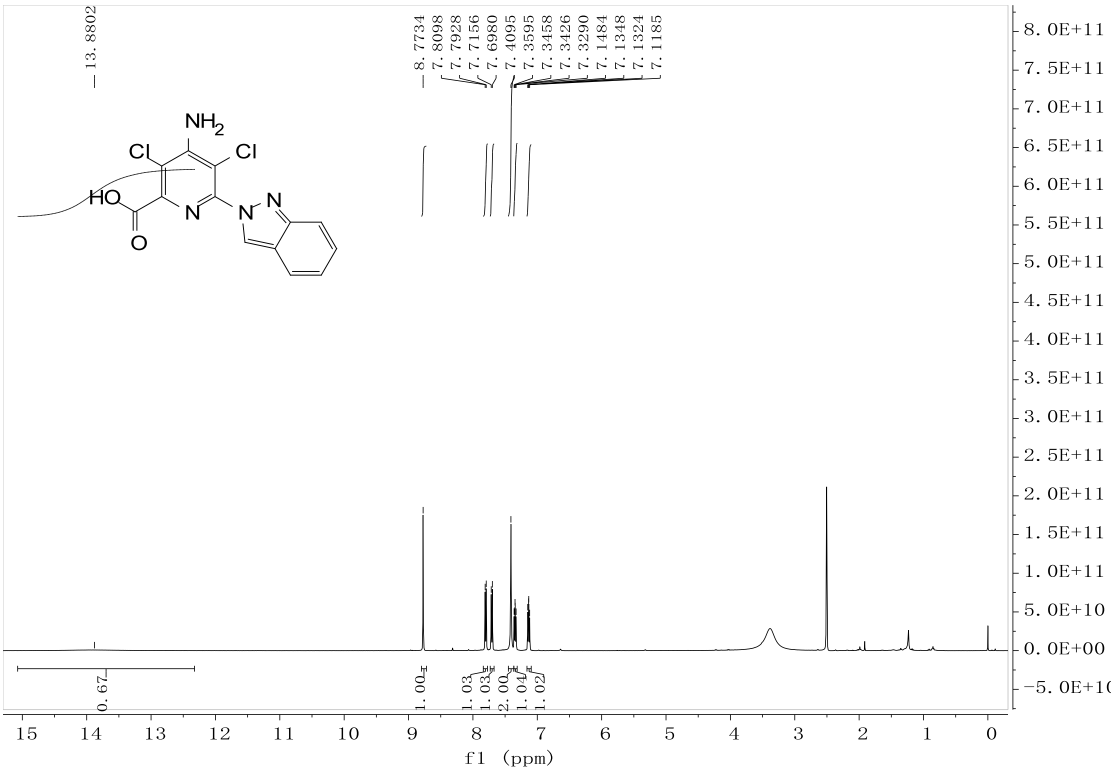
13.8802
8.7734
7.8098
7.7928
7.7156
7.6980
7.4095
7.3595
7.3458
7.3426
7.3290
7.1484
7.1348
7.1324
7.1185
NH2
Cl
Cl
HO
N
N
N
O
0.67
1.00
1.03
1.03
2.00
1.04
1.02
f1 (ppm)

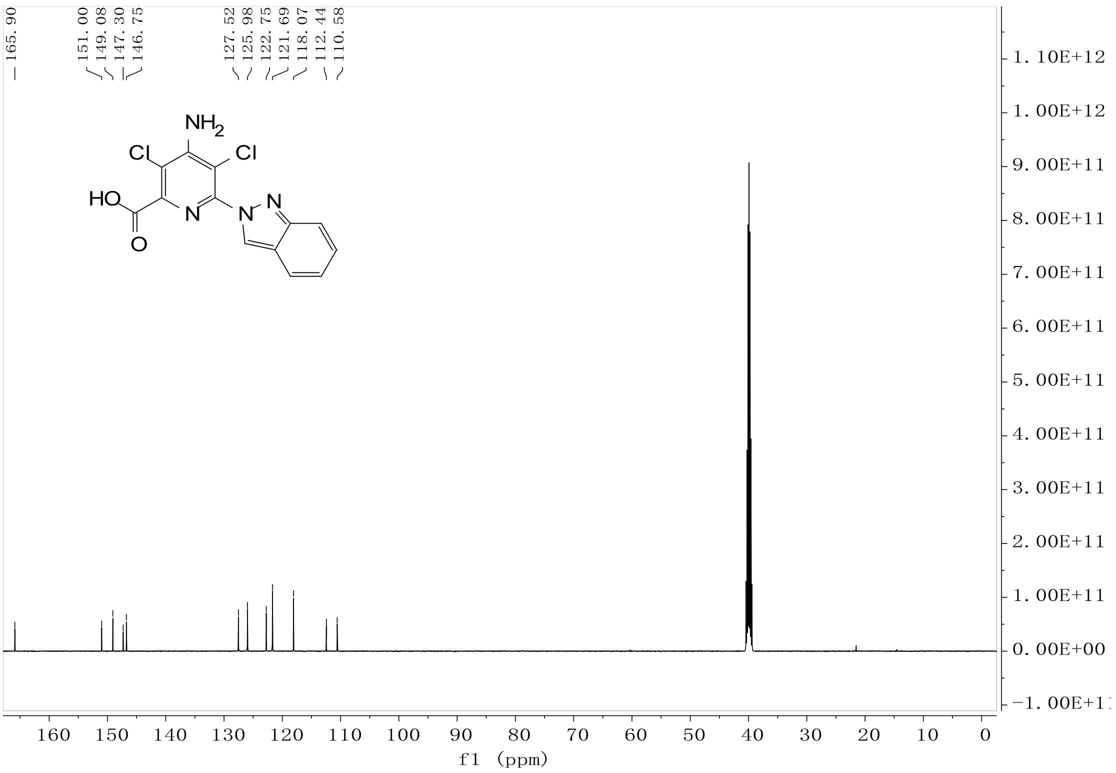
165.90
151.00
149.08
147.30
146.75
127.52
125.98
122.75
121.69
118.07
112.44
110.58
NH2
Cl
Cl
HO
N
N
N
O
f1 (ppm)

Compound 2A

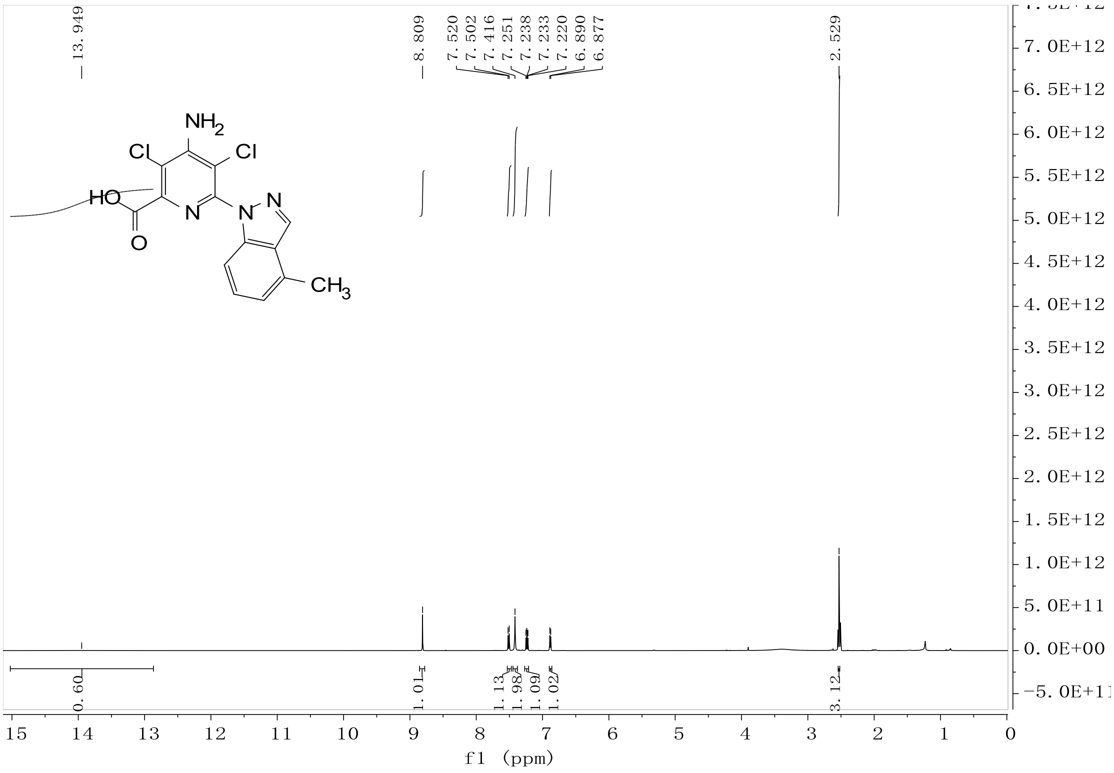
13.949
8.809
7.520
7.502
7.416
7.251
7.238
7.233
7.220
6.890
6.877
2.529
NH2
Cl
Cl
HO
N
N
N
O
CH3
0.60
1.01
1.13
1.98
1.09
1.02
3.12
f1 (ppm)

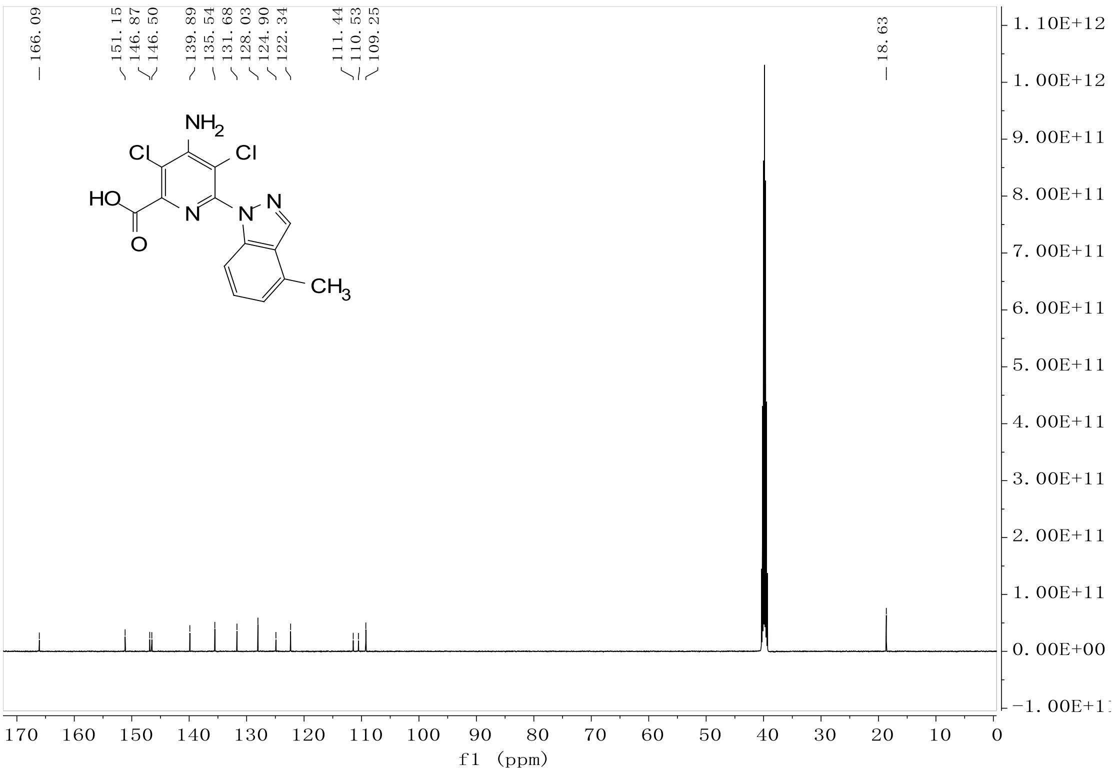
166.09
151.15
146.87
146.50
139.89
135.54
131.68
128.03
124.90
122.34
111.44
110.53
109.25
18.63
NH2
Cl
Cl
HO
N
N
N
O
CH3
f1 (ppm)

Compound 2a

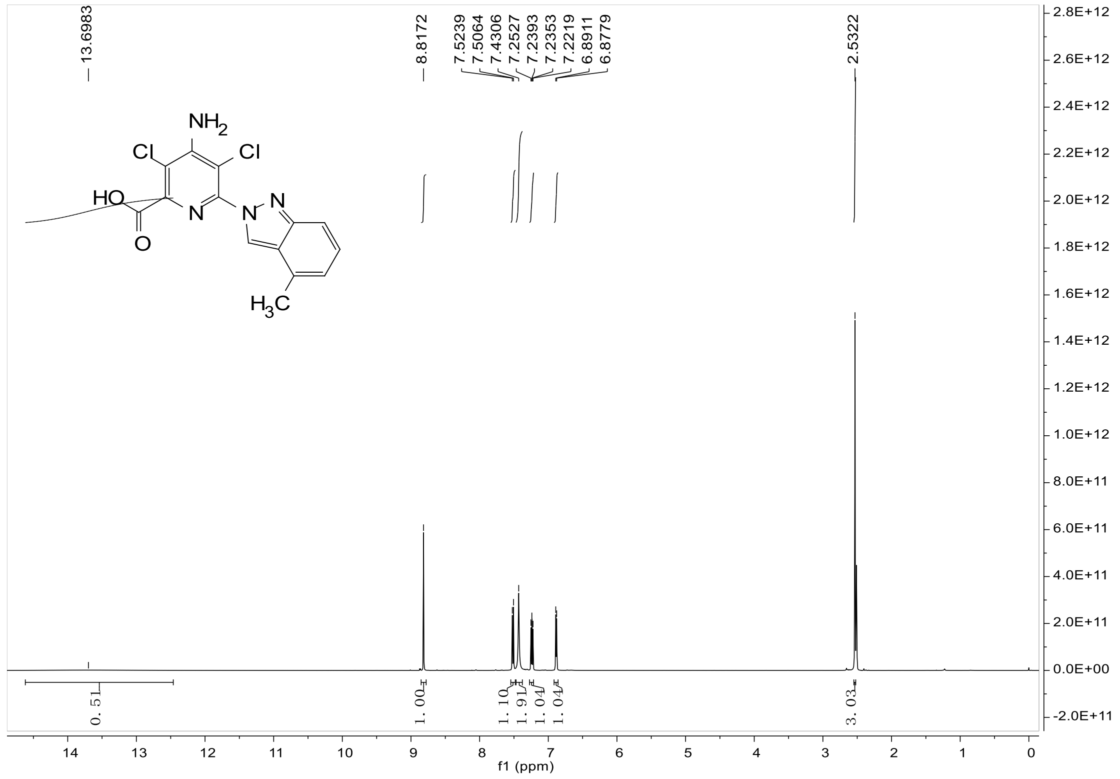
13.6983
8.8172
7.5239
7.5064
7.4306
7.2527
7.2393
7.2353
7.2219
6.8911
6.8779
2.5322
NH2
Cl
Cl
HO
N
N
N
O
H3C
0.51
1.00
1.10
1.91
1.04
1.04
3.03
f1 (ppm)

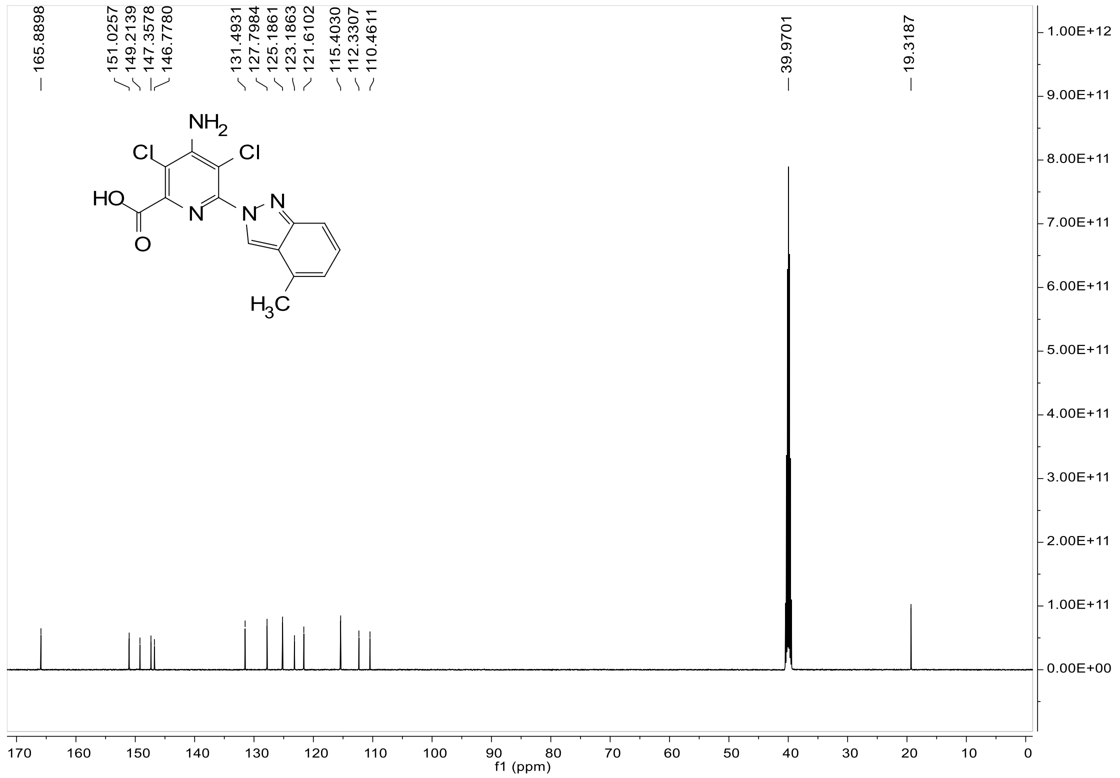
165.8898
151.0257
149.2139
147.3578
146.7780
131.4931
127.7984
125.1861
123.1863
121.6102
115.4030
112.3307
110.4611
39.9701
19.3187
NH2
Cl
Cl
HO
N
N
N
O
H3C
f1 (ppm)

Compound 2B

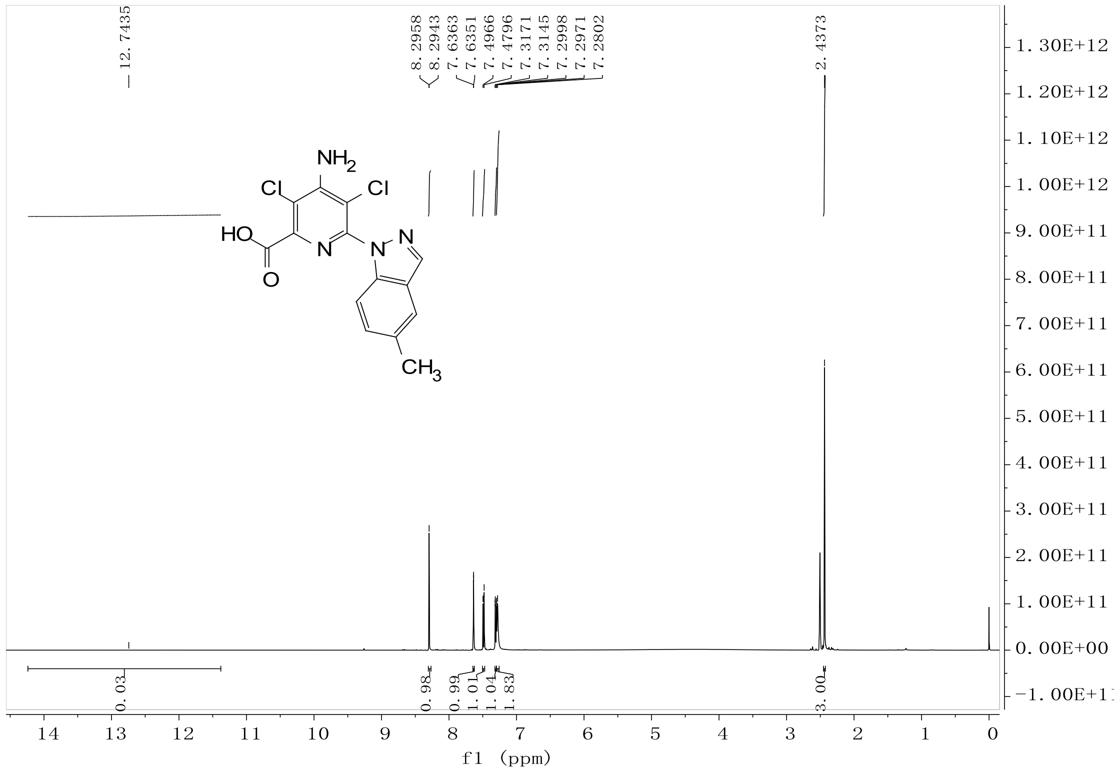

12.7435
8.2958
8.2943
7.6363
7.6351
7.4966
7.4796
7.3171
7.3145
7.2998
7.2971
7.2802
2.4373
NH2
Cl
Cl
HO
N
N
N
O
CH3
0.03
0.98
0.99
1.01
1.04
1.83
3.00
f1 (ppm)

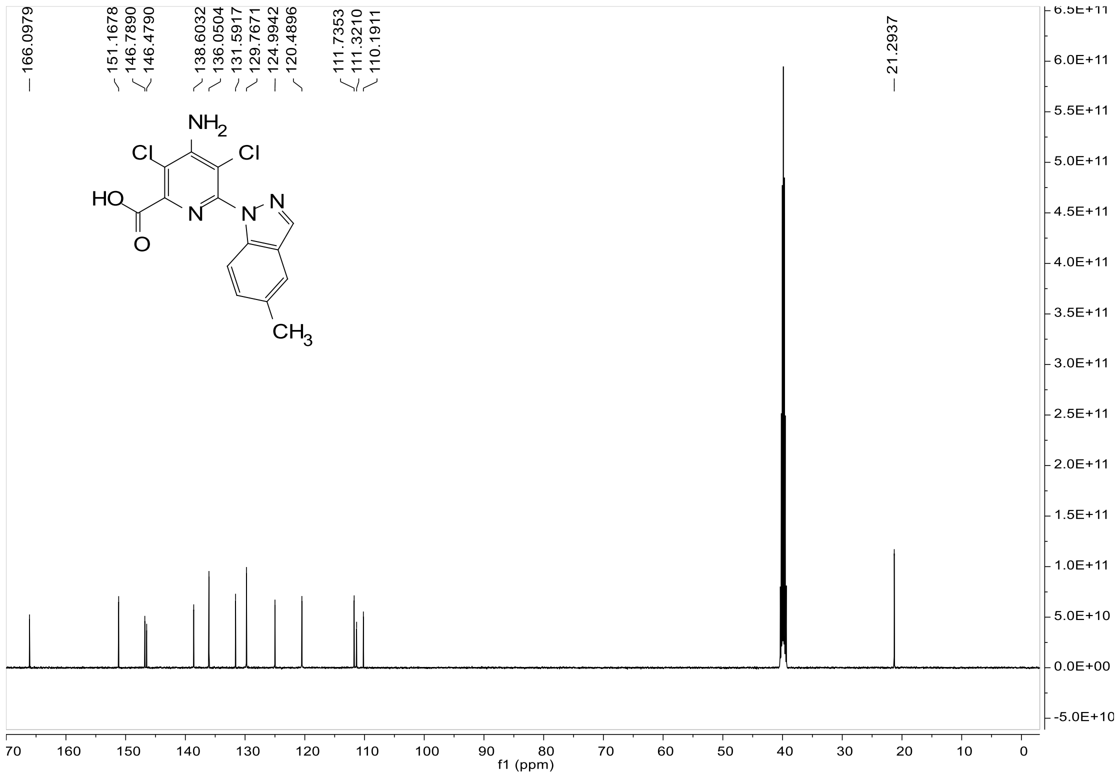

166.0979
151.1678
146.7890
146.4790
138.6032
136.0504
131.5917
129.7671
124.9942
120.4896
111.7353
111.3210
110.1911
21.2937
NH2
Cl
Cl
HO
N
N
N
O
CH3
f1 (ppm)

Compound 2b

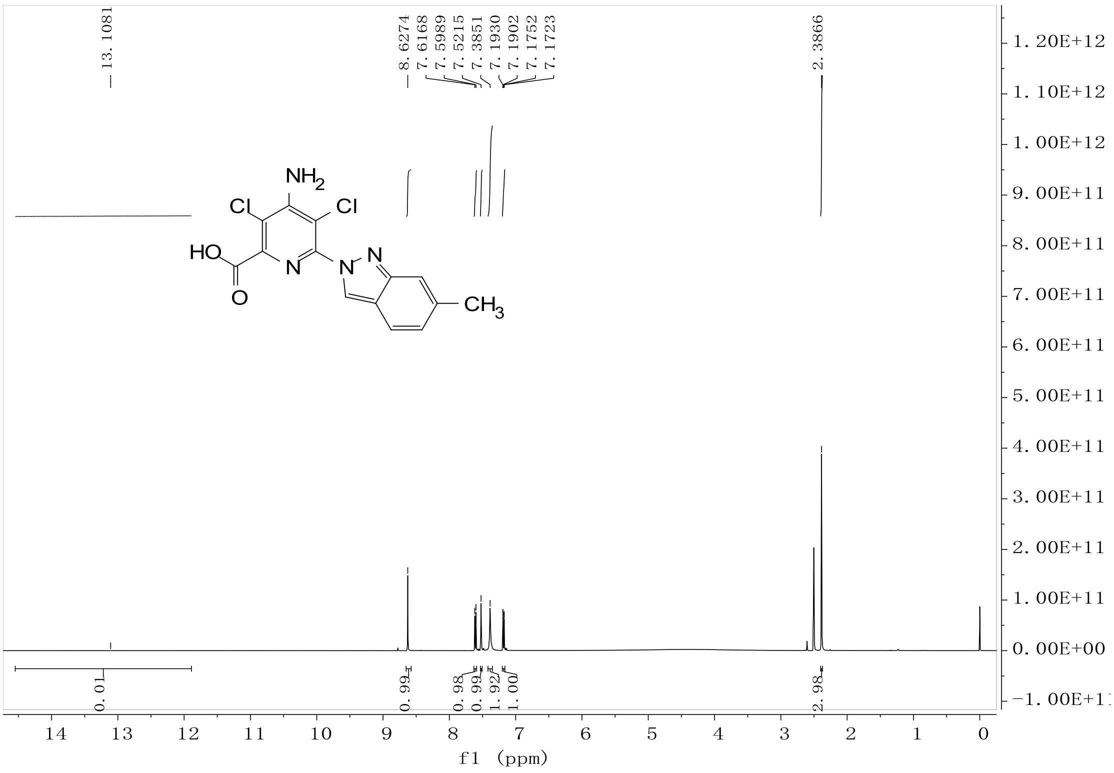
13.1081
8.6274
7.6168
7.5989
7.5215
7.3851
7.1930
7.1902
7.1752
7.1723
2.3866
NH2
Cl
Cl
HO
N
N
N
O
CH3
0.01
0.99
0.98
0.99
1.92
1.00
2.98
f1 (ppm)

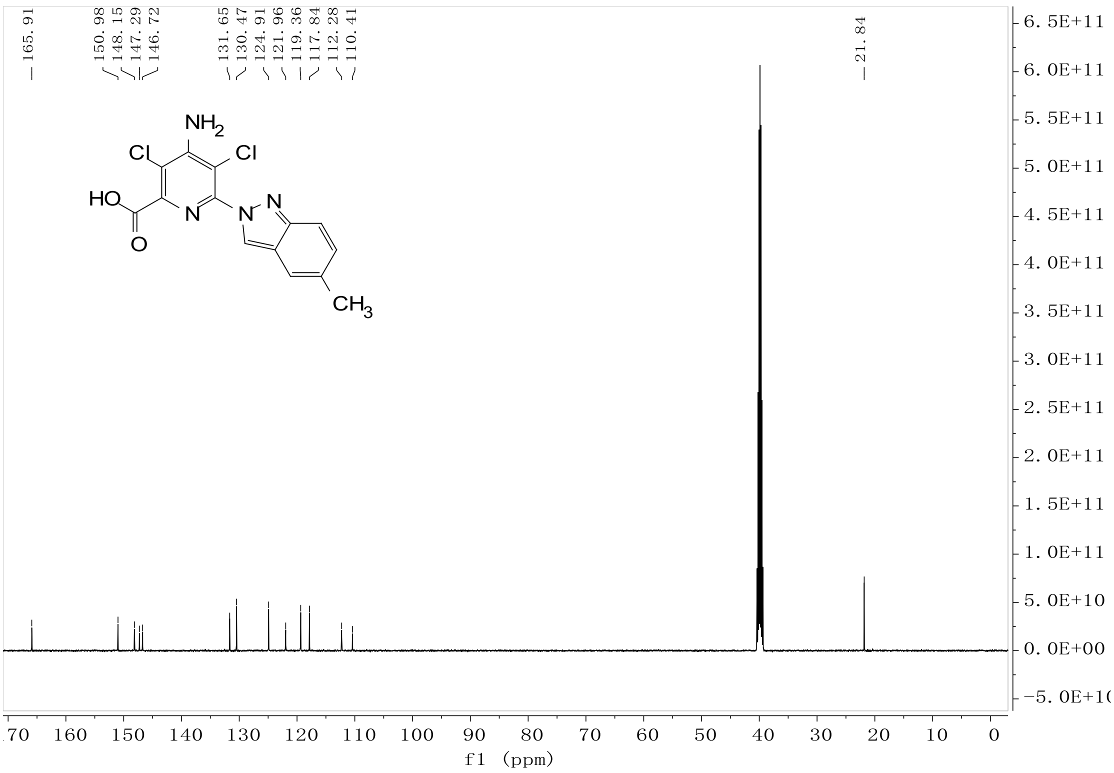
165.91
150.98
148.15
147.29
146.72
131.65
130.47
124.91
121.96
119.36
117.84
112.28
110.41
21.84
NH2
Cl
Cl
HO
N
N
N
O
CH3
f1 (ppm)

Compound 2Cc

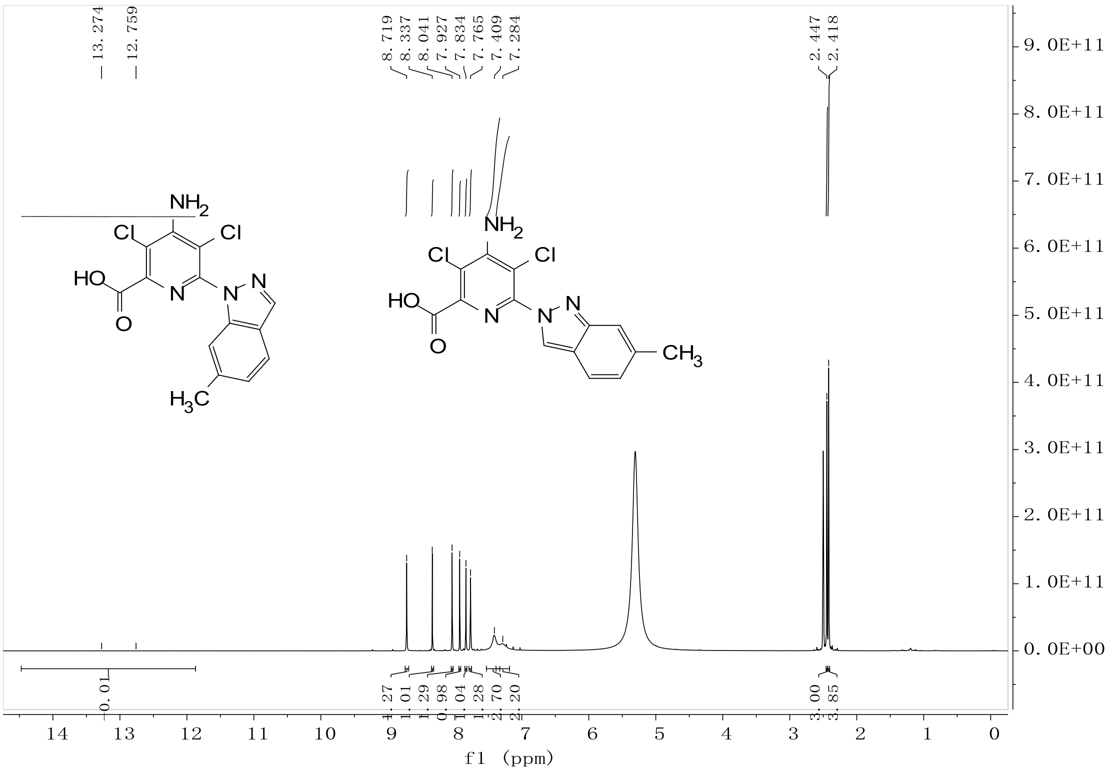

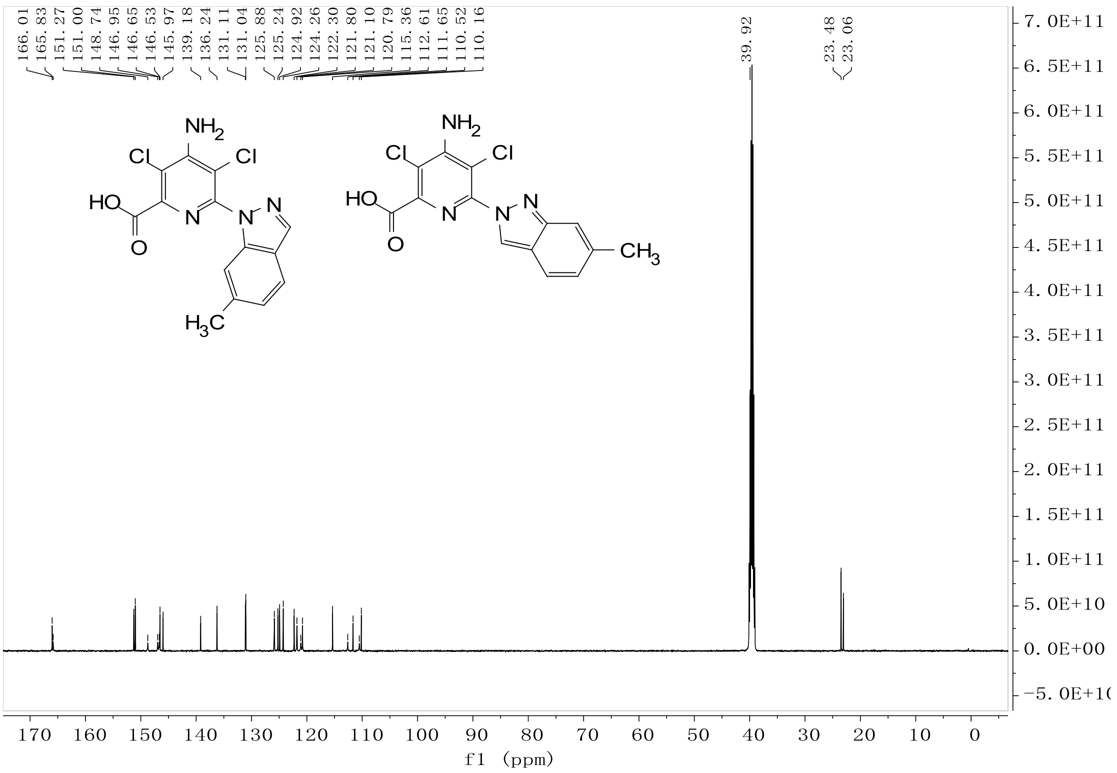

Compound 2d

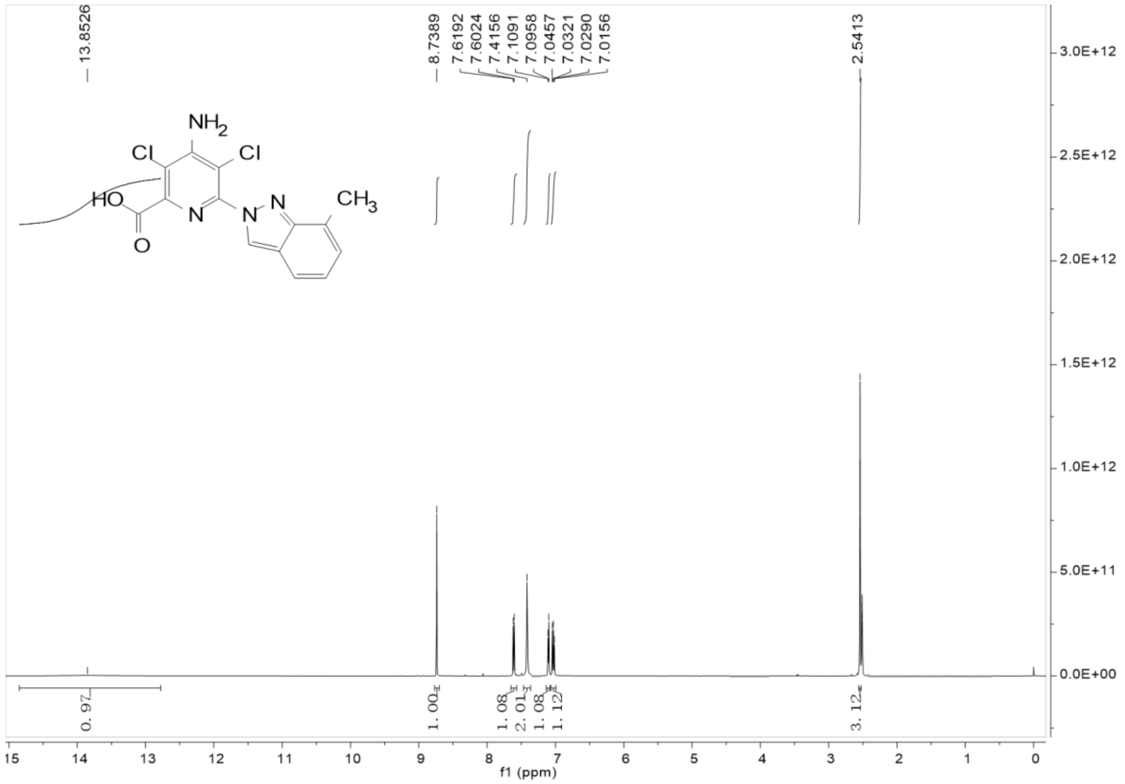
13.8526
8.7389
7.6192
7.6024
7.4156
7.1091
7.0958
7.0457
7.0321
7.0290
7.0156
2.5413
NH2
Cl
Cl
HO
N
N
N
CH3
O
0.97
1.00
1.08
2.01
1.08
1.12
3.12
f1 (ppm)

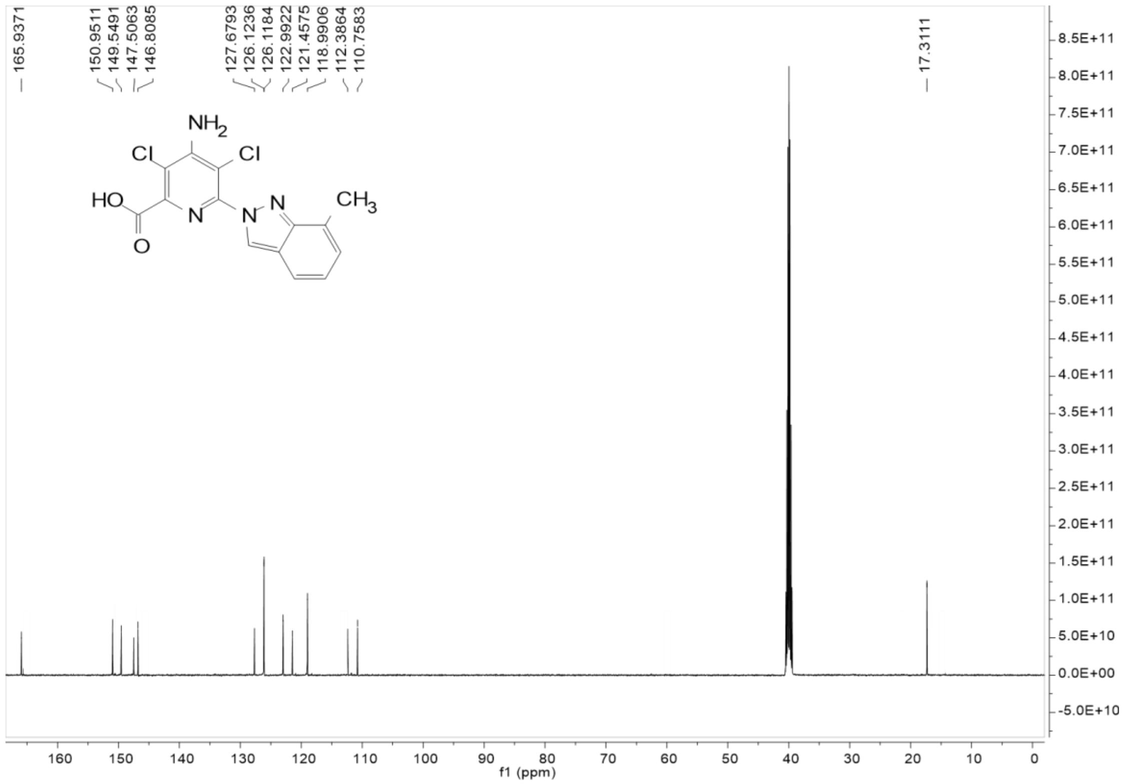
165.9371
150.9511
149.5491
147.5063
146.8085
127.6793
126.1236
126.1184
122.9922
121.4575
118.9906
112.3864
110.7583
17.3111
NH2
Cl
Cl
HO
N
N
N
CH3
O
f1 (ppm)

Compound 3A

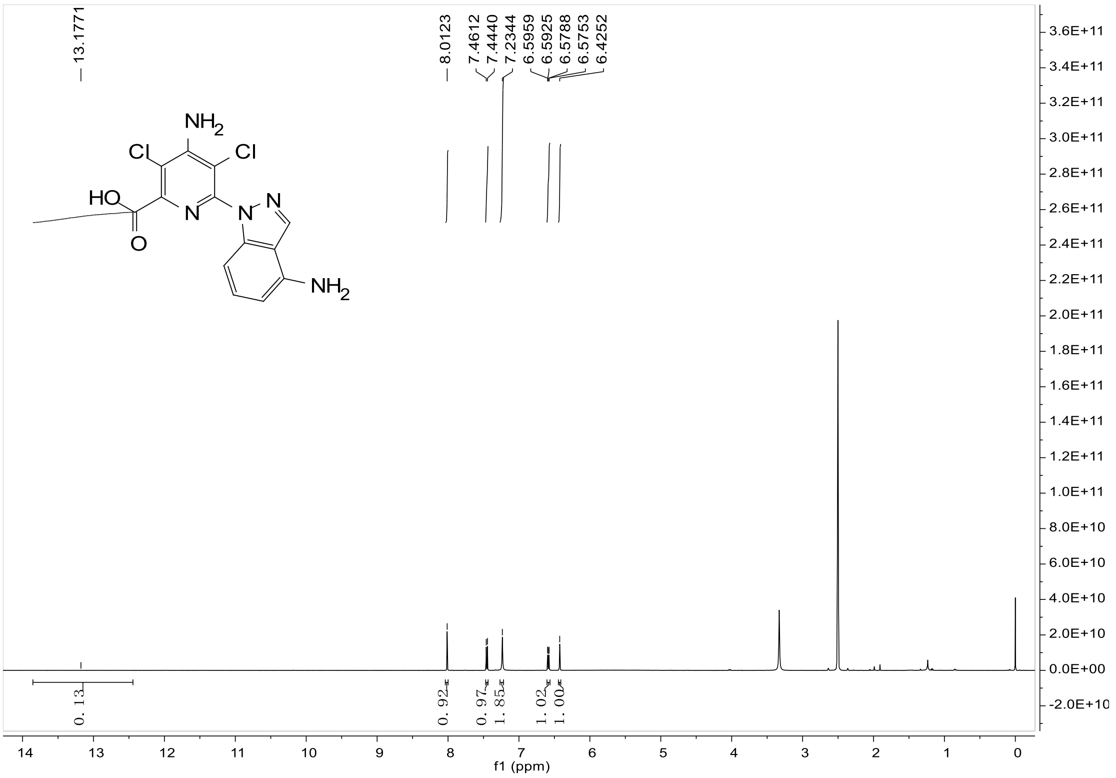
13.1771
8.0123
7.4612
7.4440
7.2344
6.5959
6.5925
6.5788
6.5753
6.4252
NH2
Cl
Cl
HO
N
N
N
O
NH2
0.13
0.92
0.97
1.85
1.02
1.00
f1 (ppm)

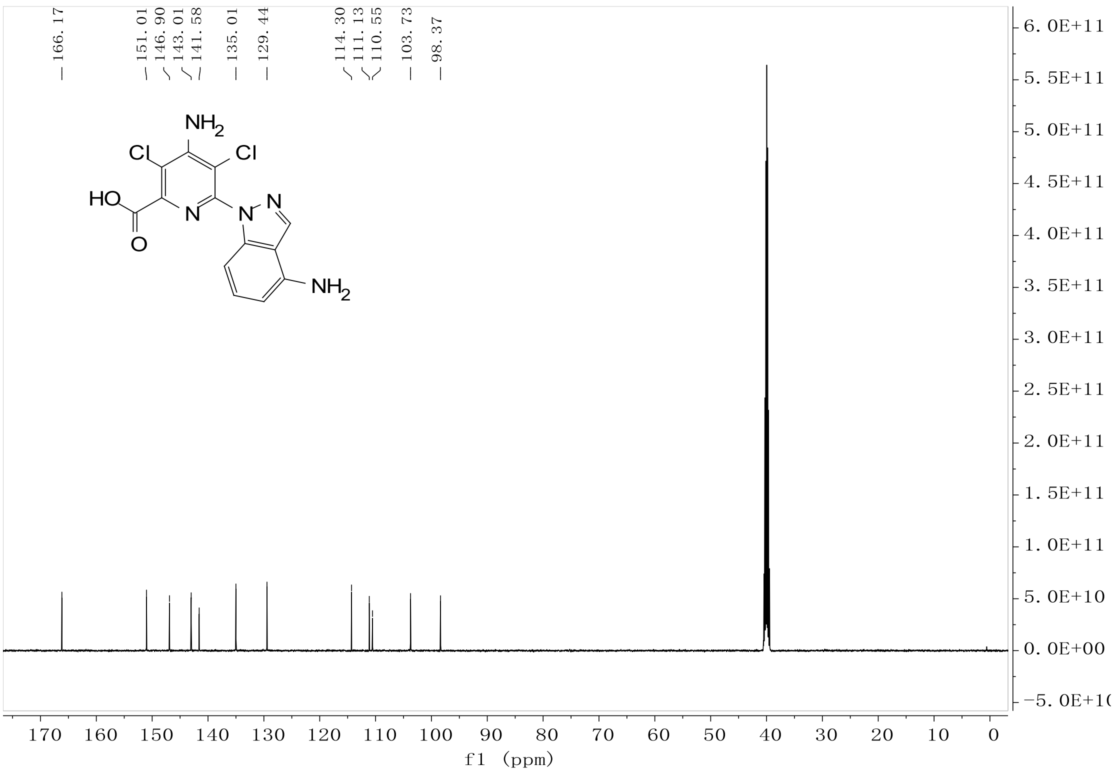
166.17
151.01
146.90
143.01
141.58
135.01
129.44
114.30
111.13
110.55
103.73
98.37
NH2
Cl
Cl
HO
N
N
N
O
NH2
f1 (ppm)

Compound 3a

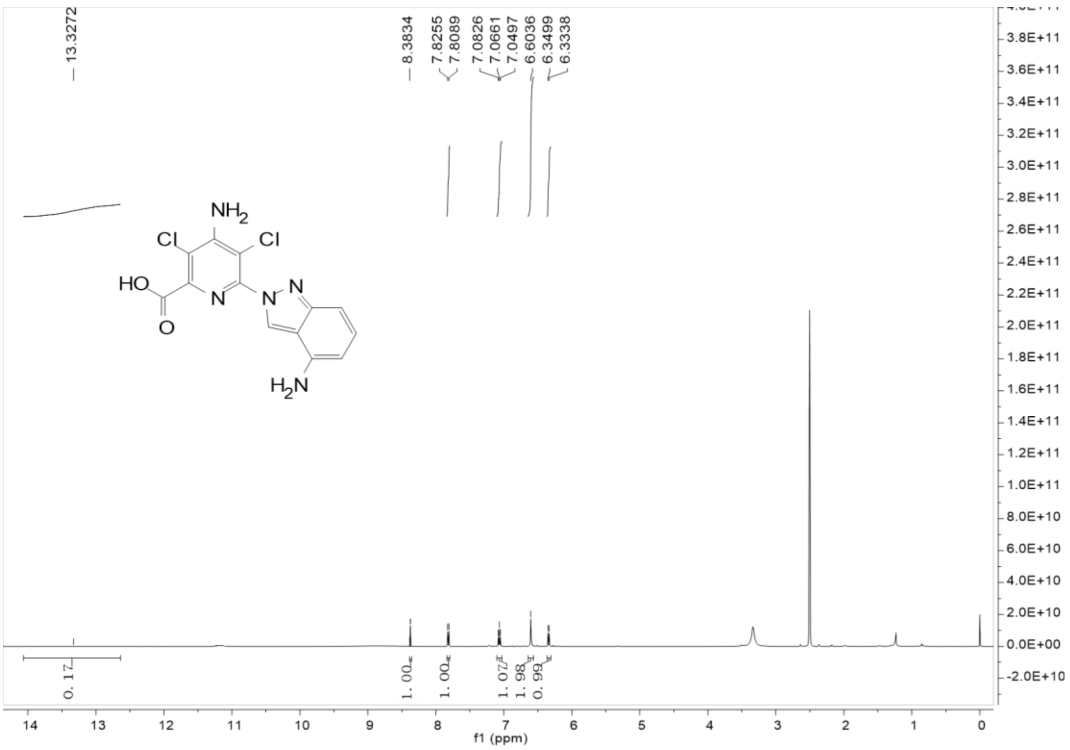
13.3272
8.3834
7.8255
7.8089
7.0826
7.0661
7.0497
6.6036
6.3499
6.3338
0.17
1.00
1.00
1.07
1.98
0.99
f1 (ppm)

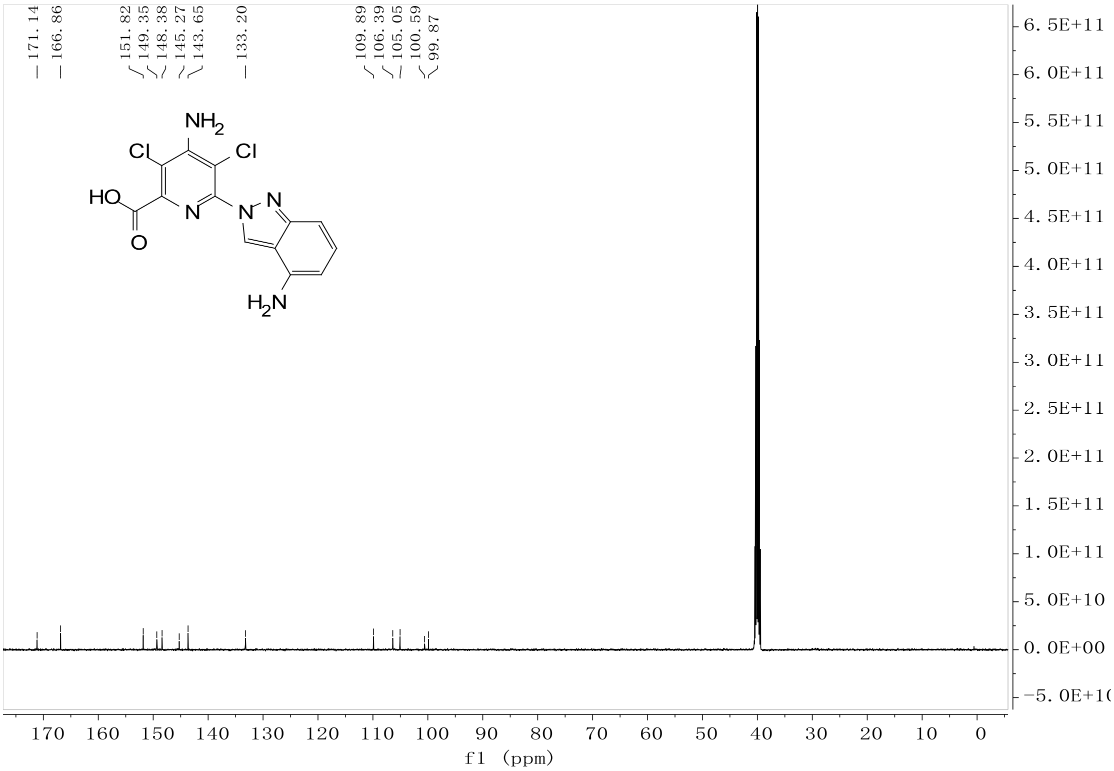
171.14
166.86
151.82
149.35
148.38
145.27
143.65
133.20
109.89
106.39
105.05
100.59
99.87
f1 (ppm)

Compound 3C

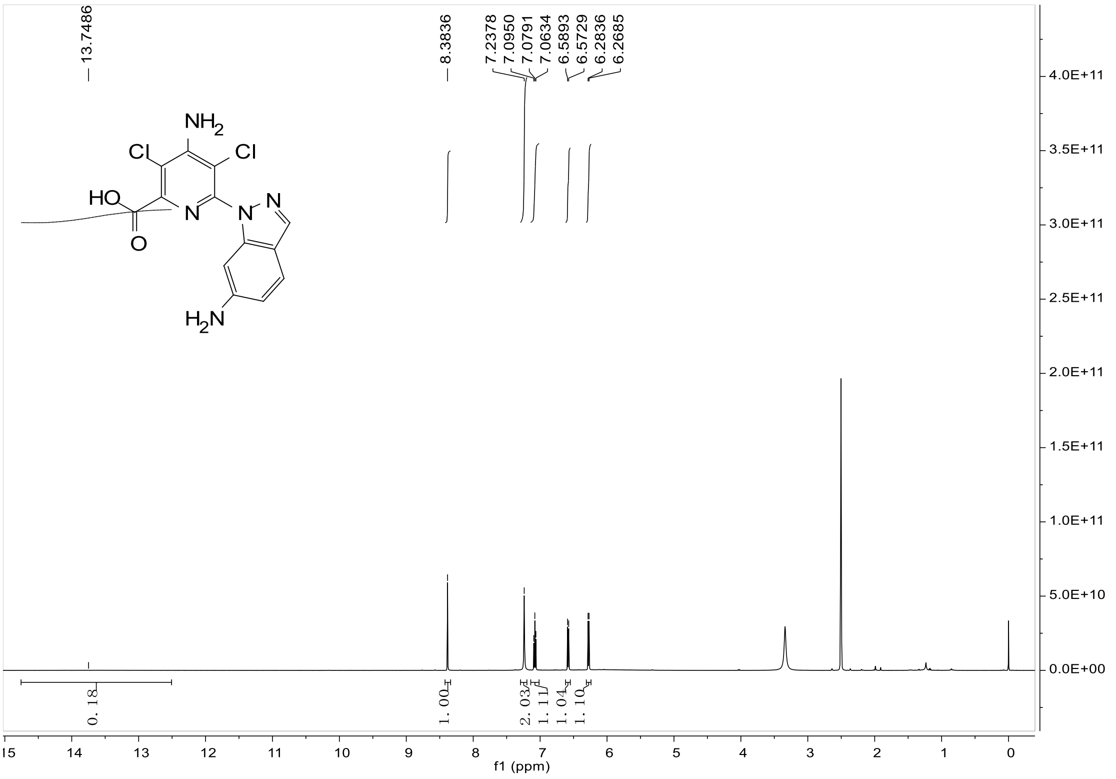

13.7486
8.3836
7.2378
7.0950
7.0791
7.0634
6.5893
6.5729
6.2836
6.2685
NH2
Cl
Cl
HO
N
N
N
O
H2N
0.18
1.00
2.03
1.11
1.04
1.10
f1 (ppm)

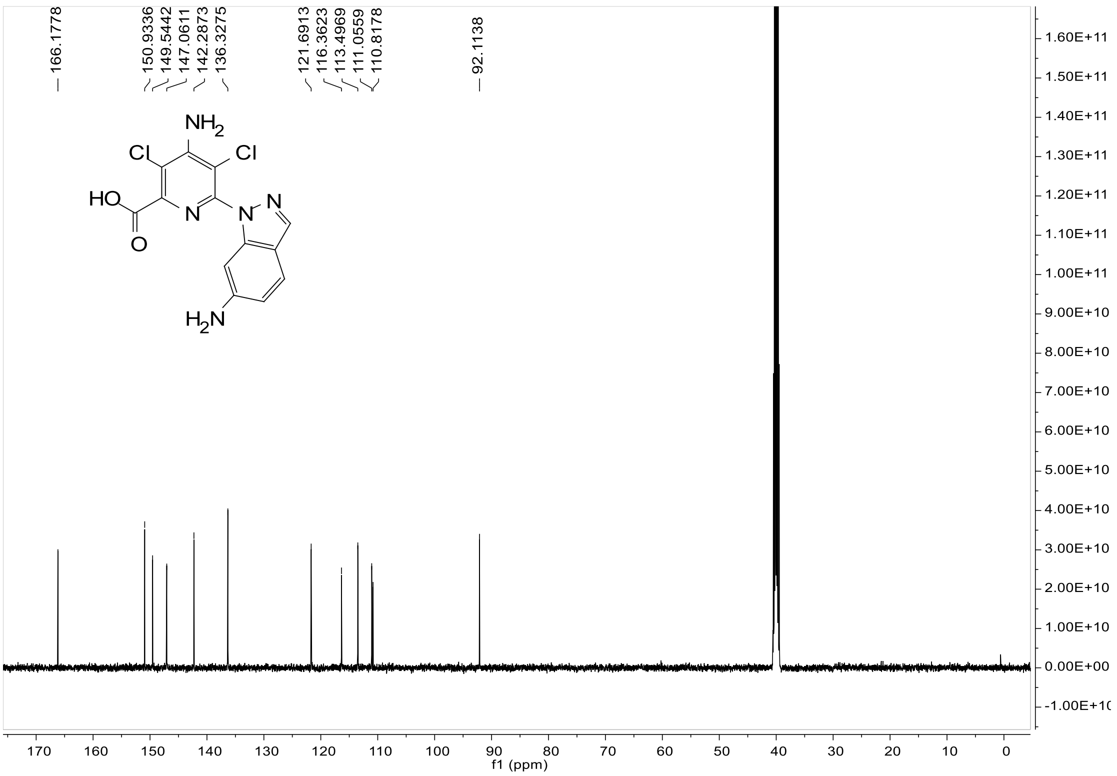

166.1778
150.9336
149.5442
147.0611
142.2873
136.3275
121.6913
116.3623
113.4969
111.0559
110.8178
92.1138
NH2
Cl
Cl
HO
N
N
N
O
H2N
f1 (ppm)

Compound 3c

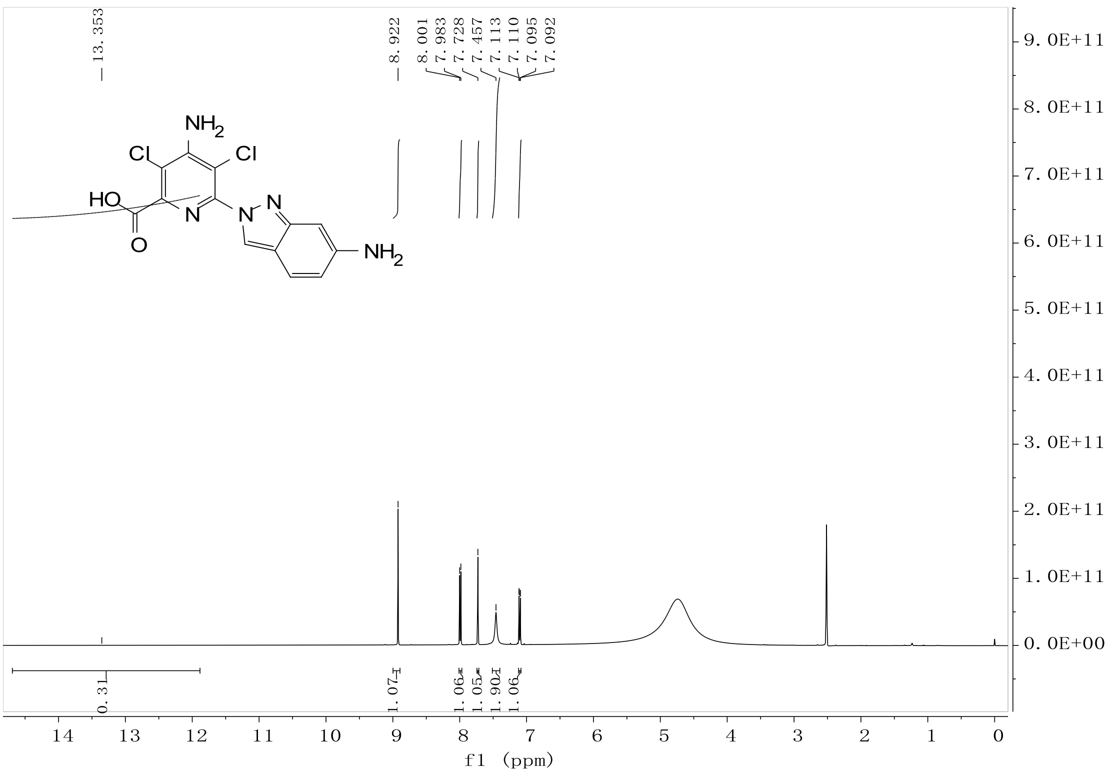
13.353
8.922
8.001
7.983
7.728
7.457
7.113
7.110
7.095
7.092
NH2
Cl
Cl
HO
N
N
N
O
NH2
0.31
1.07
1.06
1.05
1.90
1.06
f1 (ppm)

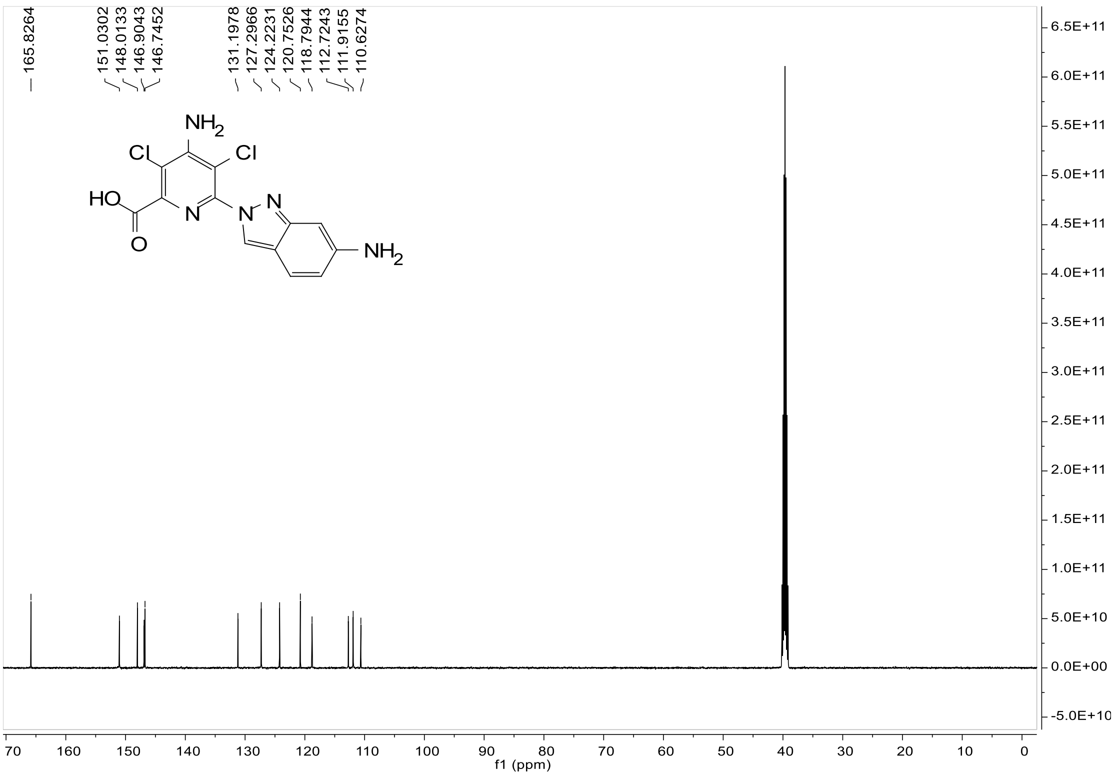
165.8264
151.0302
148.0133
146.9043
146.7452
131.1978
127.2966
124.2231
120.7526
118.7944
112.7243
111.9155
110.6274
NH2
Cl
Cl
HO
N
N
N
O
NH2
f1 (ppm)

Compound 3d

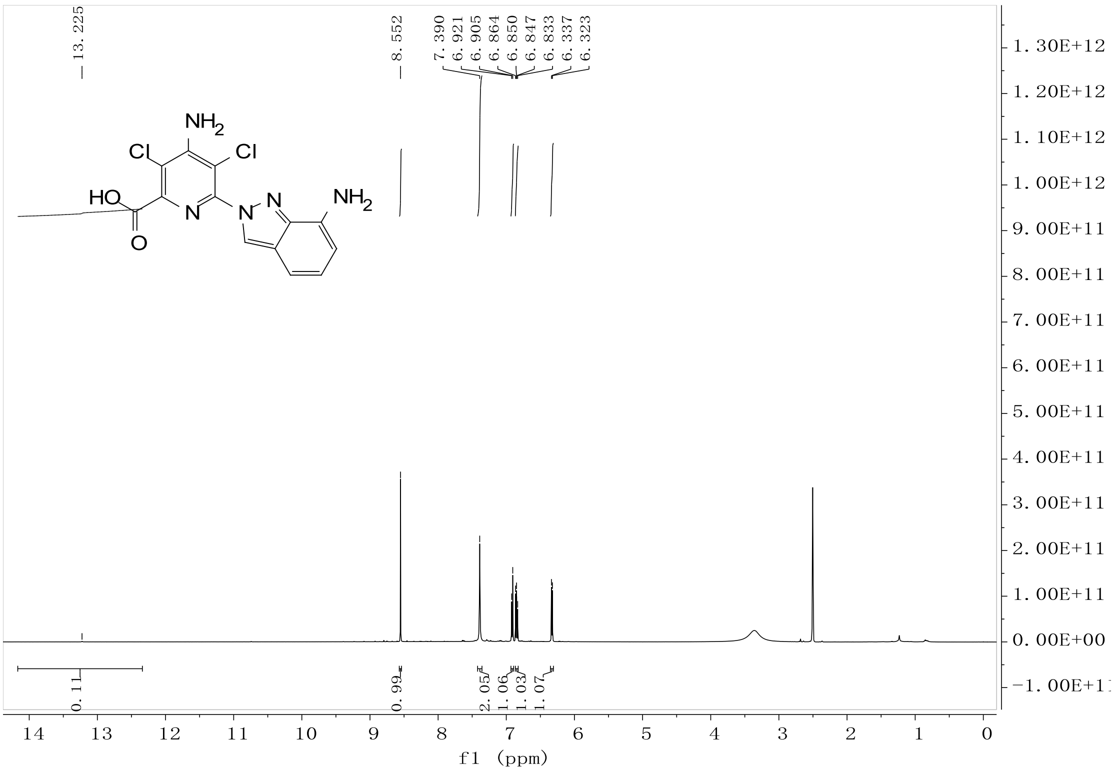

13.225
8.552
7.390
6.921
6.905
6.864
6.850
6.847
6.833
6.337
6.323
NH2
Cl
Cl
HO
N
N
N
NH2
O
0.11
0.99
2.05
1.06
1.03
1.07
f1 (ppm)

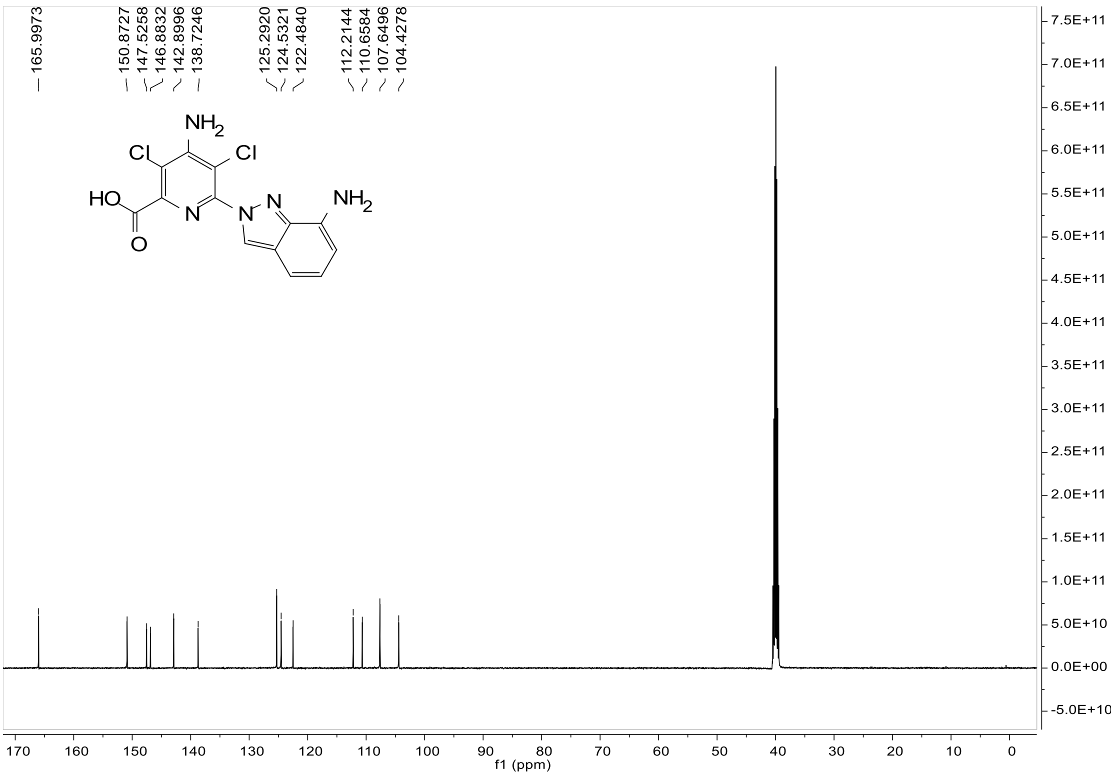

165.9973
150.8727
147.5258
146.8832
142.8996
138.7246
125.2920
124.5321
122.4840
112.2144
110.6584
107.6496
104.4278
NH2
Cl
Cl
HO
N
N
N
NH2
O
f1 (ppm)

Compound 4A

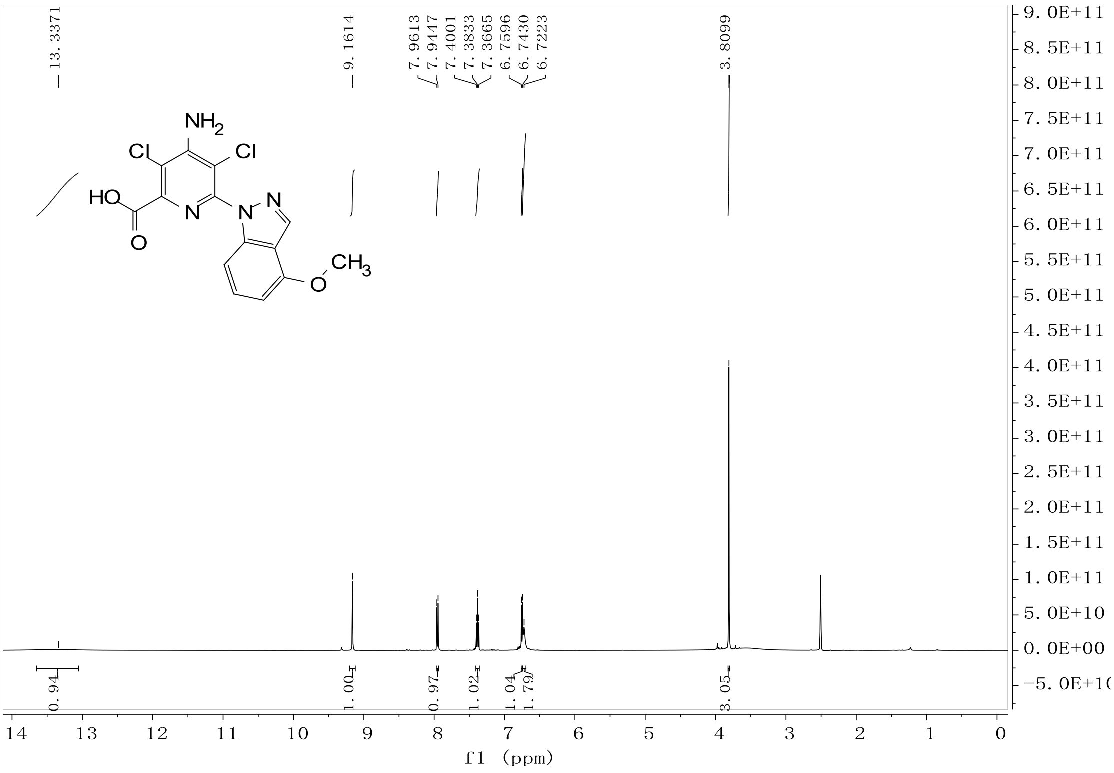
13.3371
9.1614
7.9613
7.9447
7.4001
7.3833
7.3665
6.7596
6.7430
6.7223
3.8099
0.94
1.00
0.97
1.02
1.04
1.79
3.05
f1 (ppm)

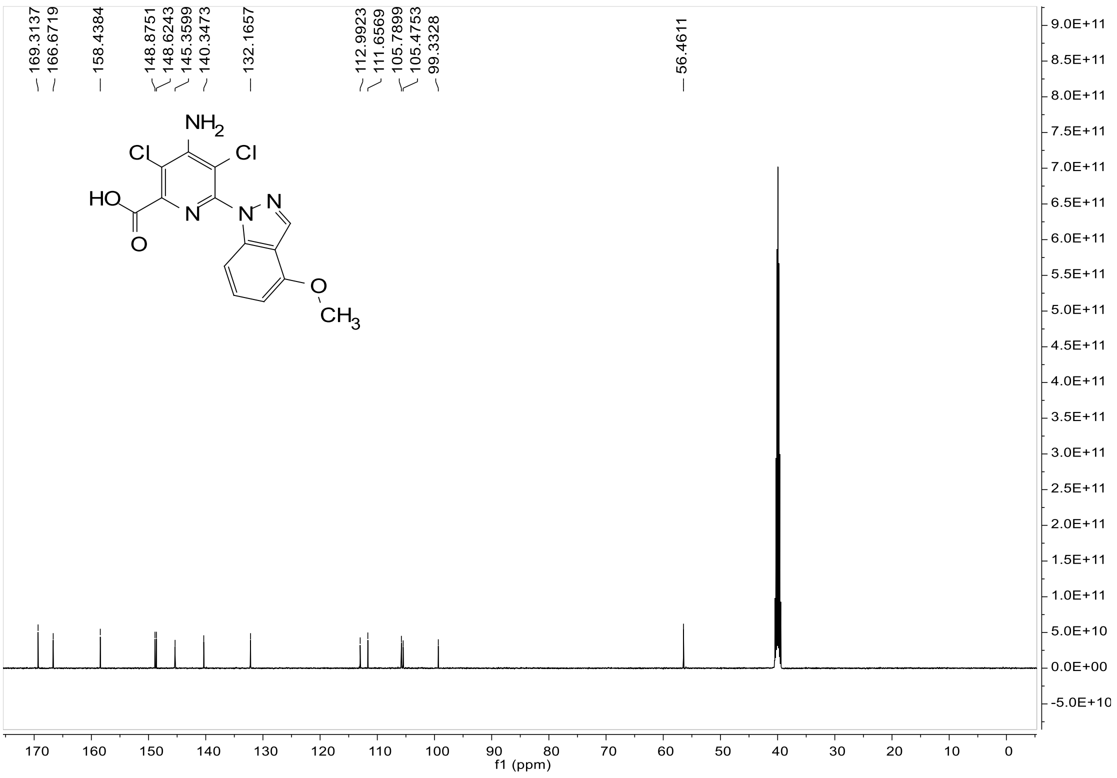
169.3137
166.6719
158.4384
148.8751
148.6243
145.3599
140.3473
132.1657
112.9923
111.6569
105.7899
105.4753
99.3328
56.4611
f1 (ppm)

Compound 4a

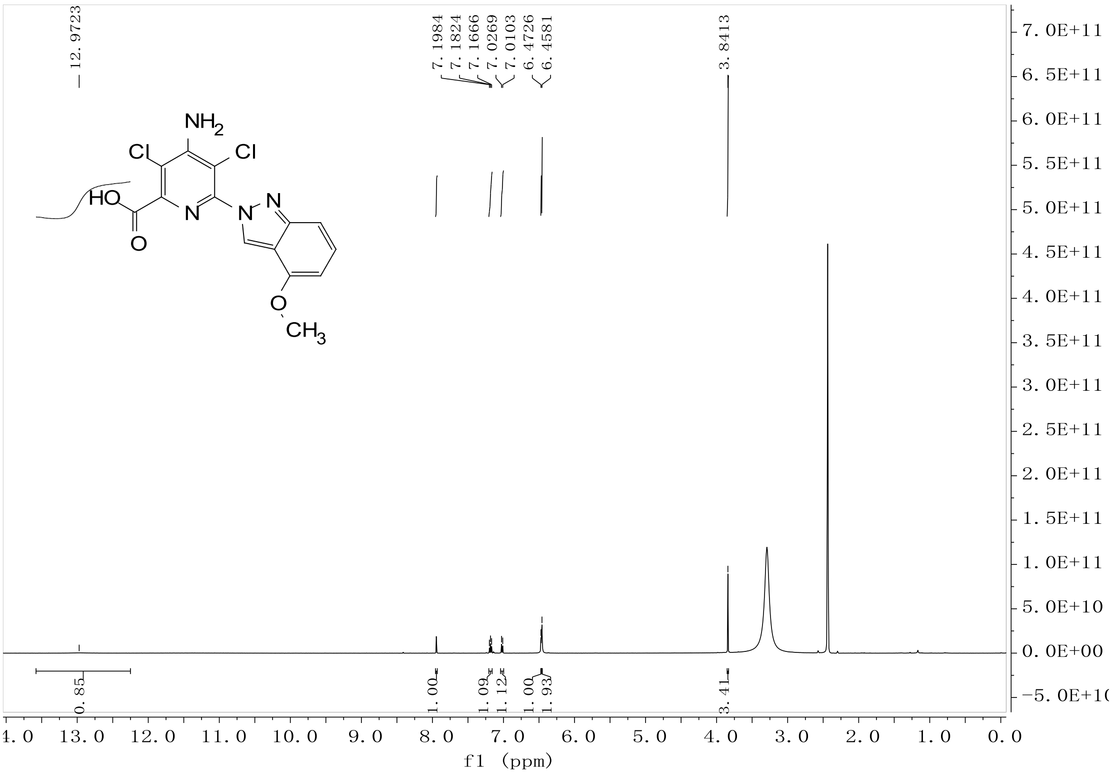
12.9723
7.1984
7.1824
7.1666
7.0269
7.0103
6.4726
6.4581
3.8413
NH2
Cl
Cl
HO
N
N
N
O
O
CH3
0.85
1.00
1.09
1.12
1.00
1.93
3.41
f1 (ppm)

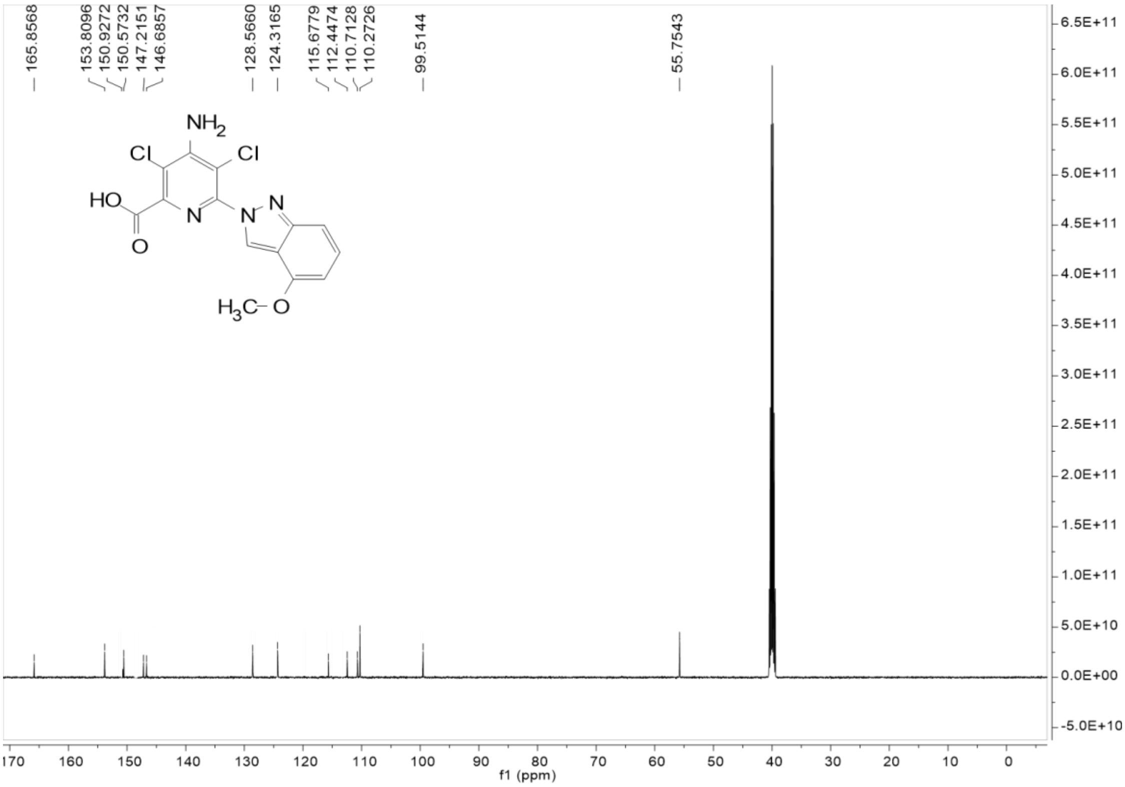
165.8568
153.8096
150.9272
150.5732
147.2151
146.6857
128.5660
124.3165
115.6779
112.4474
110.7128
110.2726
99.5144
55.7543
NH2
Cl
Cl
HO
N
N
N
O
H3C–O
f1 (ppm)

Compound 4B

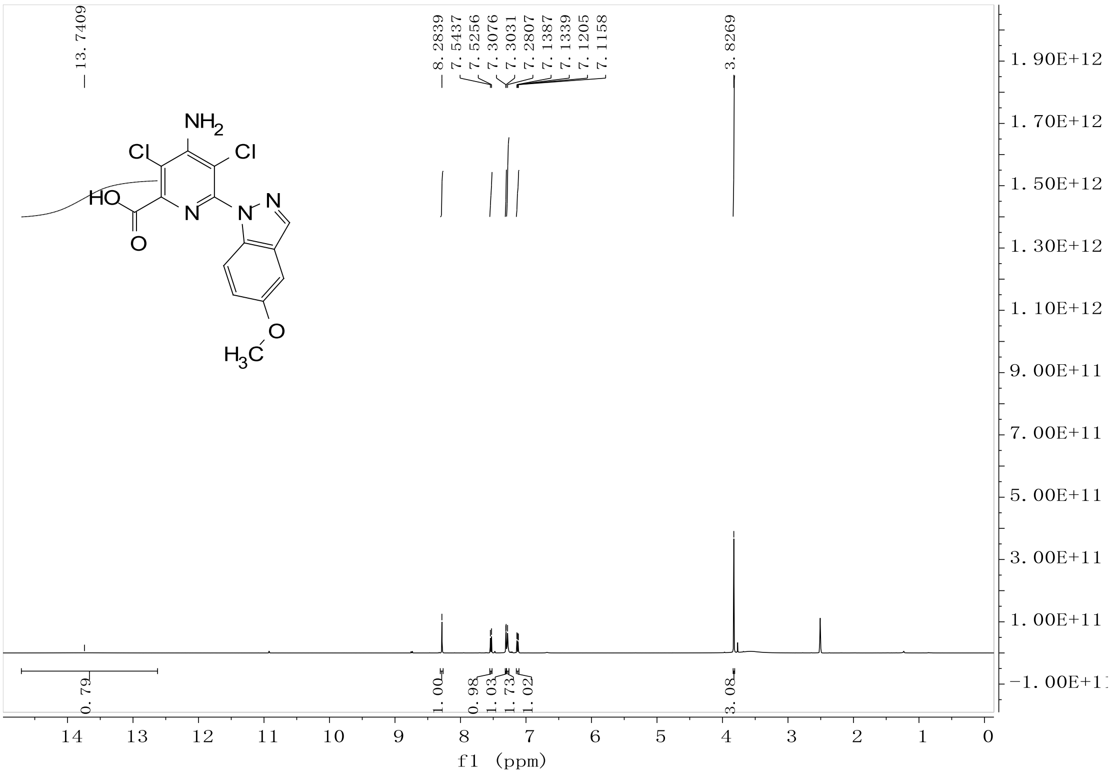
13.7409
8.2839
7.5437
7.5256
7.3076
7.3031
7.2807
7.1387
7.1339
7.1205
7.1158
3.8269
NH2
Cl
Cl
HO
N
N
N
O
O
H3C
0.79
1.00
0.98
1.03
1.73
1.02
3.08
1.90E+12
1.70E+12
1.50E+12
1.30E+12
1.10E+12
9.00E+11
7.00E+11
5.00E+11
3.00E+11
1.00E+11
-1.00E+11
f1 (ppm)

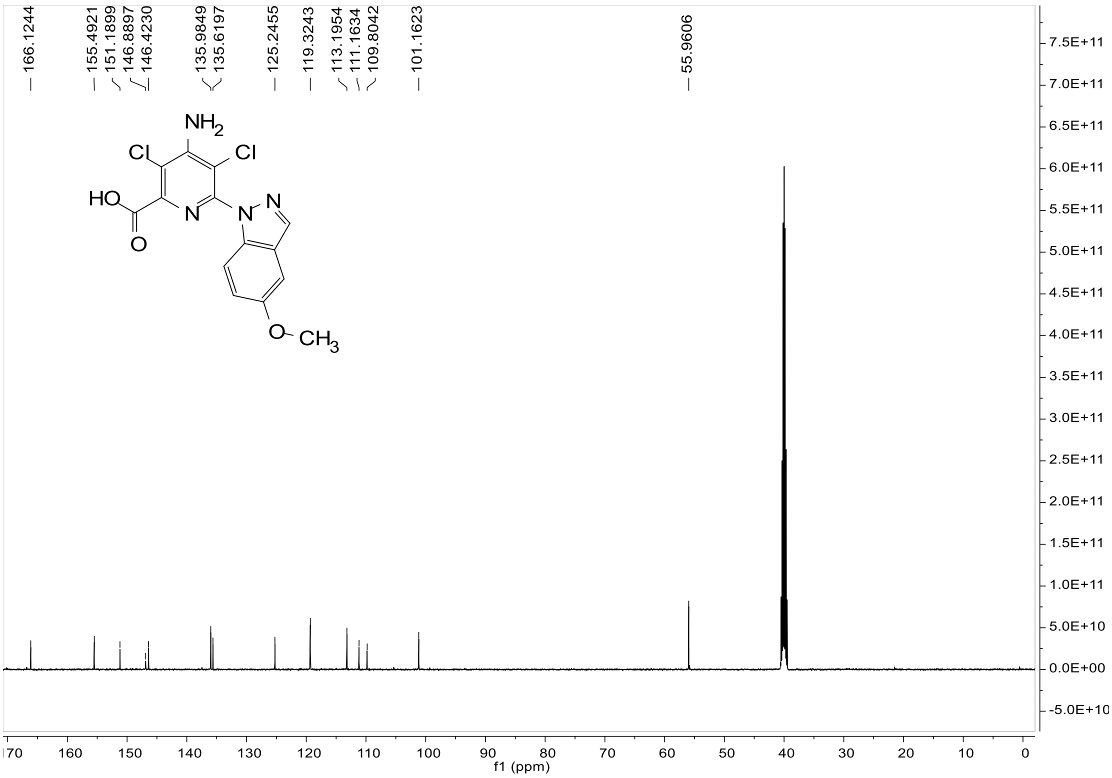
166.1244
155.4921
151.1899
146.8897
146.4230
135.9849
135.6197
125.2455
119.3243
113.1954
111.1634
109.8042
101.1623
55.9606
NH2
Cl
Cl
HO
N
N
N
O
O
CH3
f1 (ppm)

Compound 4b

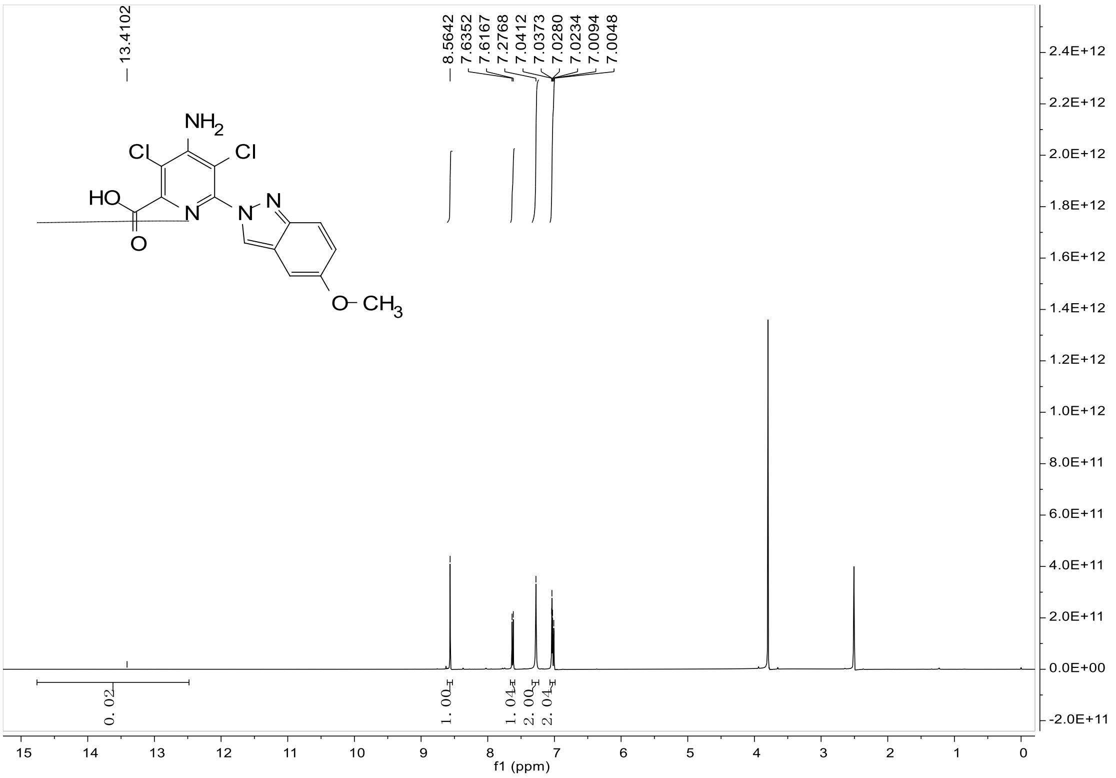

13.4102
8.5642
7.6352
7.6167
7.2768
7.0412
7.0373
7.0280
7.0234
7.0094
7.0048
NH2
Cl
Cl
HO
N
N
N
O
O- CH3
0.02
1.00
1.04
2.00
2.04
f1 (ppm)

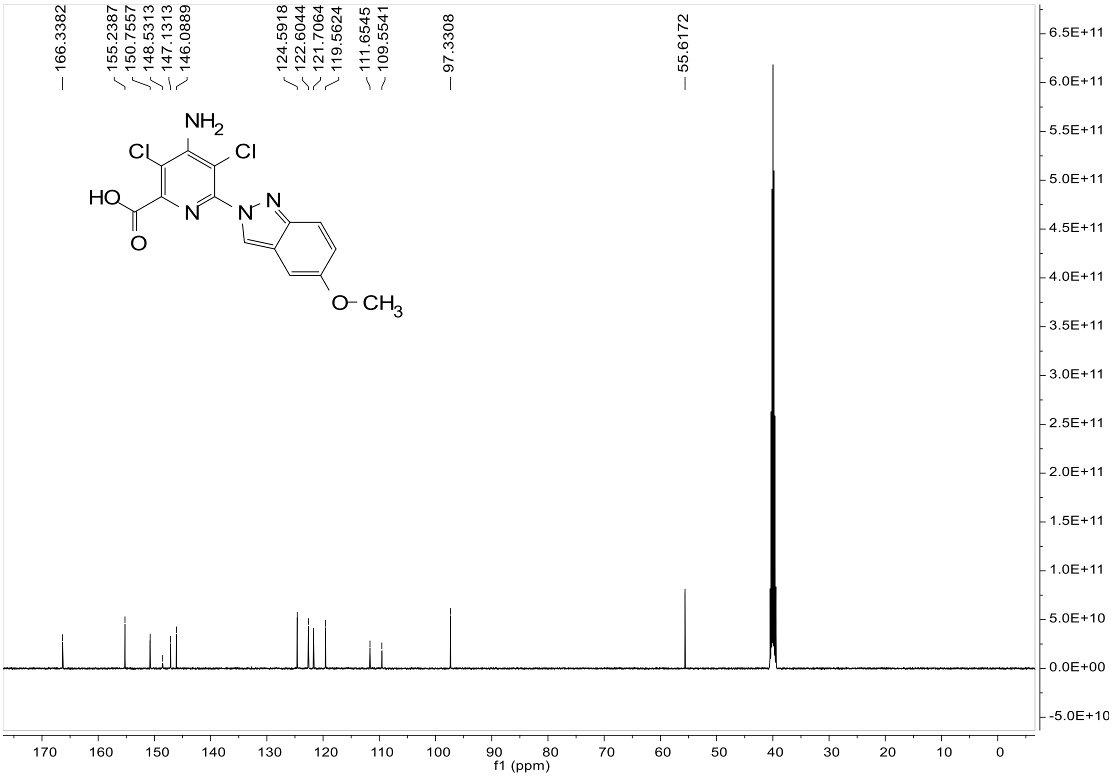

166.3382
155.2387
150.7557
148.5313
147.1313
146.0889
124.5918
122.6044
121.7064
119.5624
111.6545
109.5541
97.3308
55.6172
NH2
Cl
Cl
HO
N
N
N
O
O- CH3
f1 (ppm)

Compound 4C

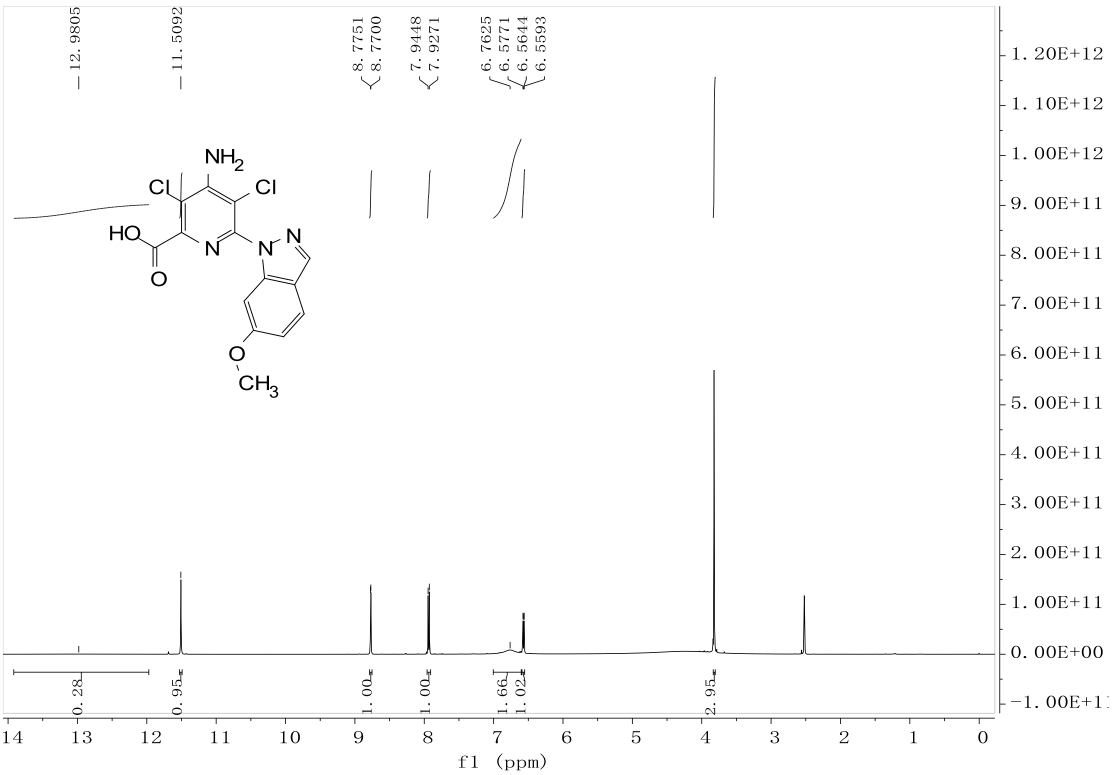
12.9805
11.5092
8.7751
8.7700
7.9448
7.9271
6.7625
6.5771
6.5644
6.5593
NH2
Cl
Cl
HO
N
N
N
O
O
CH3
0.28
0.95
1.00
1.00
1.66
1.02
2.95
f1 (ppm)

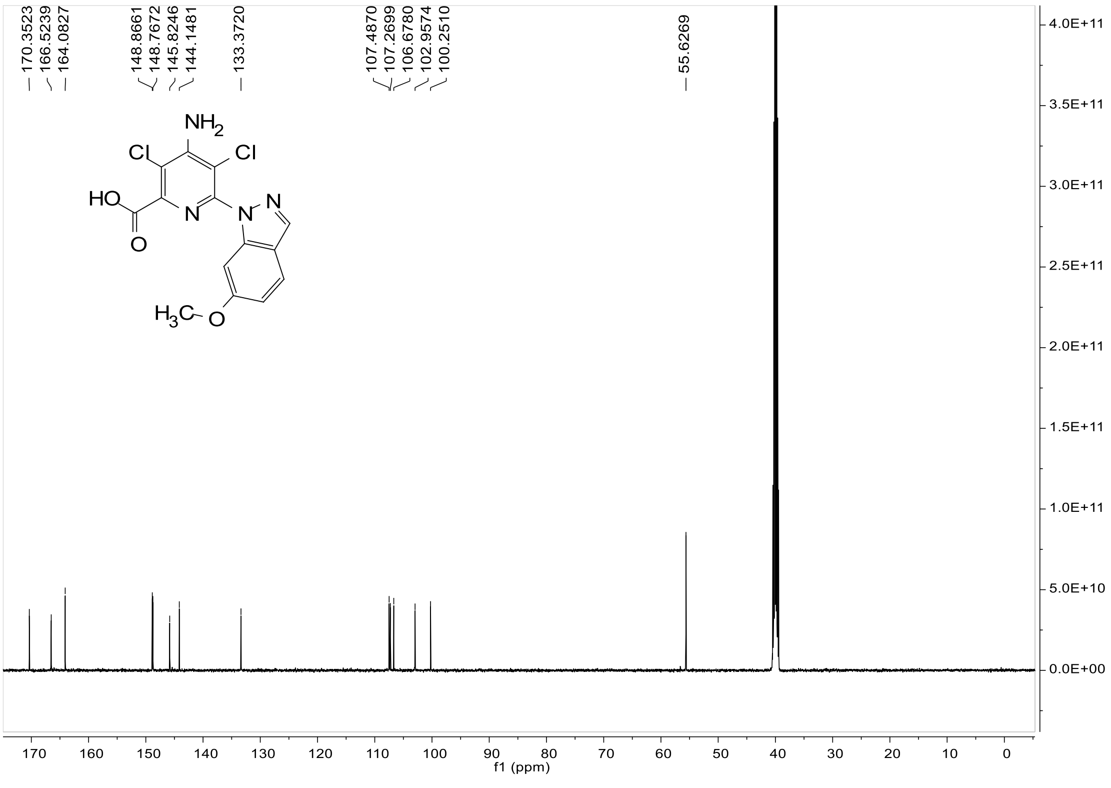
170.3523
166.5239
164.0827
148.8661
148.7672
145.8246
144.1481
133.3720
107.4870
107.2699
106.6780
102.9574
100.2510
55.6269
NH2
Cl
Cl
HO
N
N
N
O
H3C
O
f1 (ppm)

Compound 4c

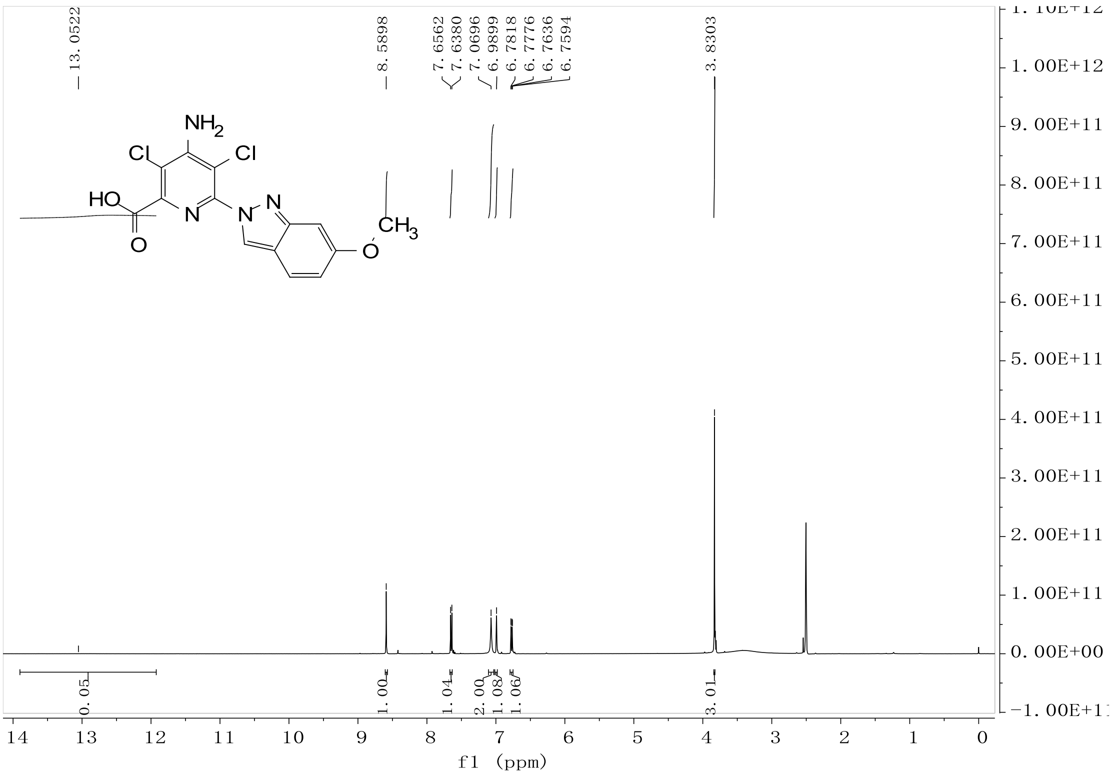
13.0522
8.5898
7.6562
7.6380
7.0696
6.9899
6.7818
6.7776
6.7636
6.7594
3.8303
NH2
Cl
Cl
HO
N
N
N
O
O
CH3
0.05
1.00
1.04
2.00
1.08
1.06
3.01
f1 (ppm)

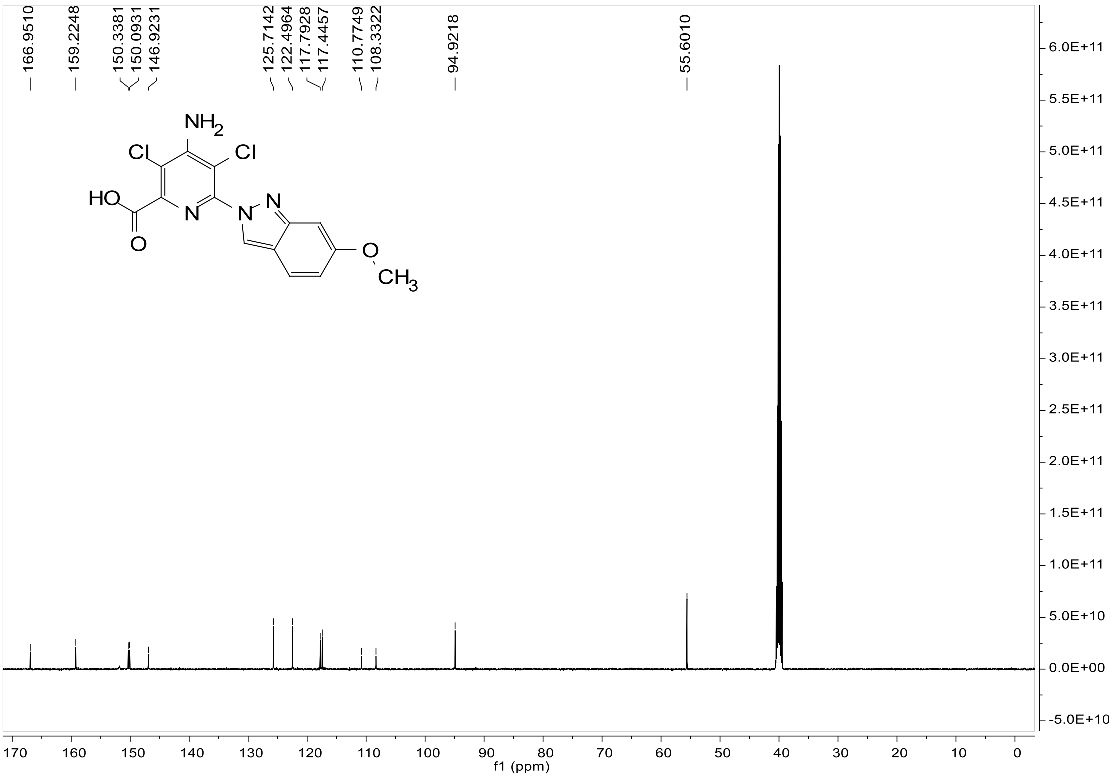
166.9510
159.2248
150.3381
150.0931
146.9231
125.7142
122.4964
117.7928
117.4457
110.7749
108.3322
94.9218
55.6010
NH2
Cl
Cl
HO
N
N
N
O
O
CH3
f1 (ppm)

Compound 4d

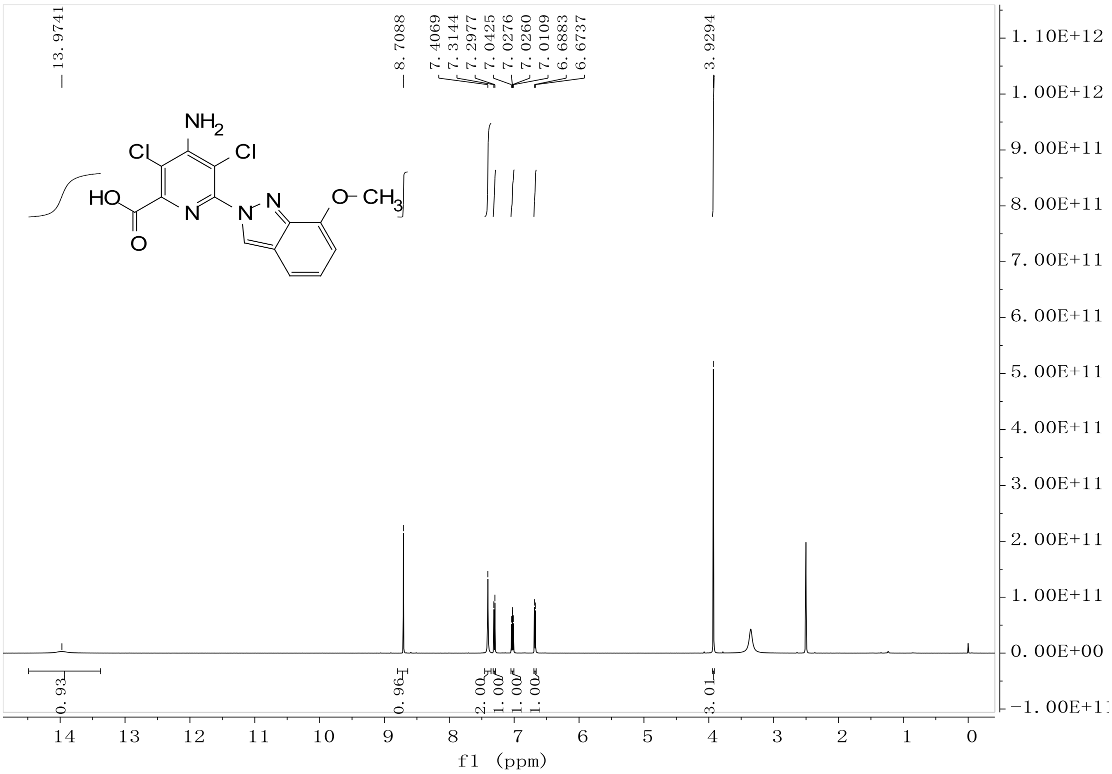
13.9741
8.7088
7.4069
7.3144
7.2977
7.0425
7.0276
7.0260
7.0109
6.6883
6.6737
3.9294
NH2
Cl
Cl
HO
N
N
N
O
CH3
O
0.93
0.96
2.00
1.00
1.00
1.00
3.01
f1 (ppm)

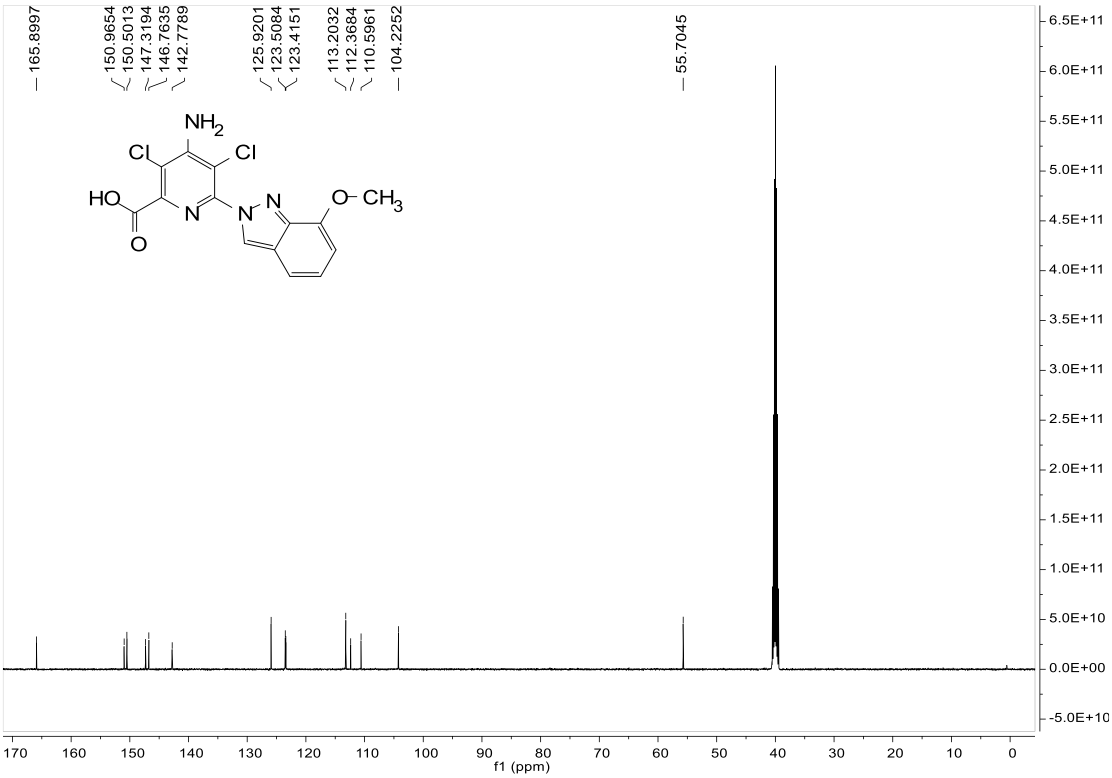
165.8997
150.9654
150.5013
147.3194
146.7635
142.7789
125.9201
123.5084
123.4151
113.2032
112.3684
110.5961
104.2252
55.7045
NH2
Cl
Cl
HO
N
N
N
O
CH3
O
f1 (ppm)

Compound 5A

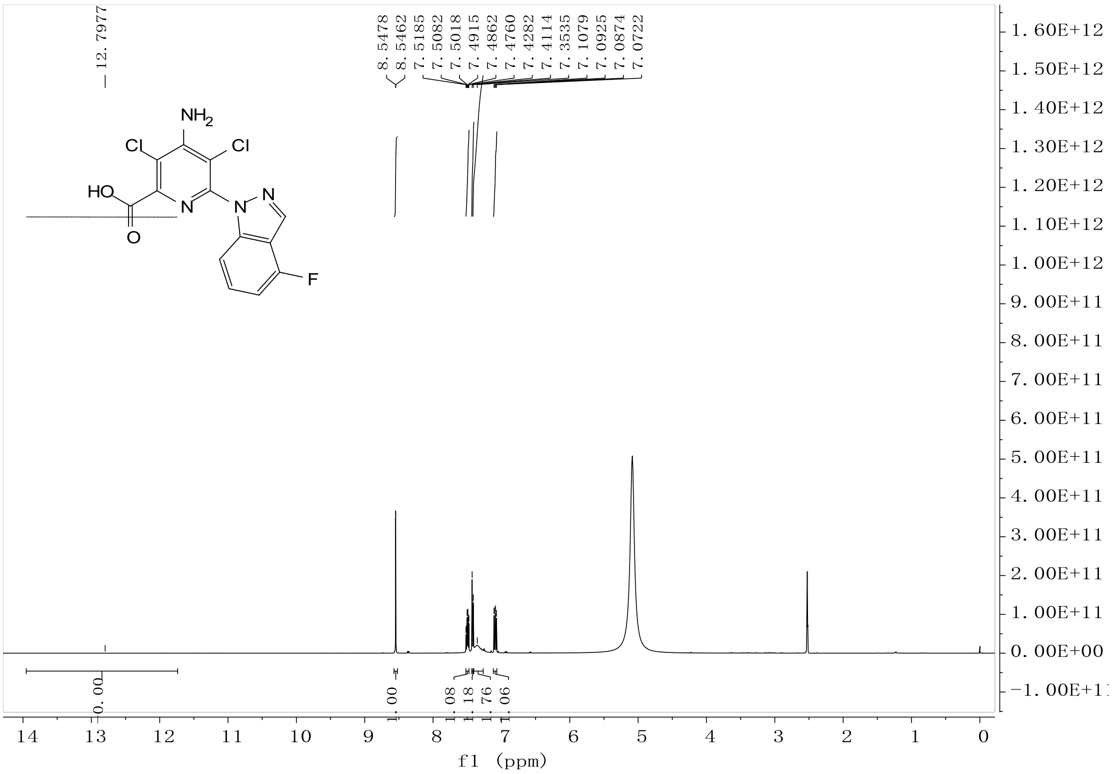

12.7977
8.5478
8.5462
7.5185
7.5082
7.5018
7.4915
7.4862
7.4760
7.4282
7.4114
7.3535
7.1079
7.0925
7.0874
7.0722
NH2
Cl
Cl
HO
N
N
N
O
F
0.00
1.00
1.08
1.18
1.76
1.06
f1 (ppm)

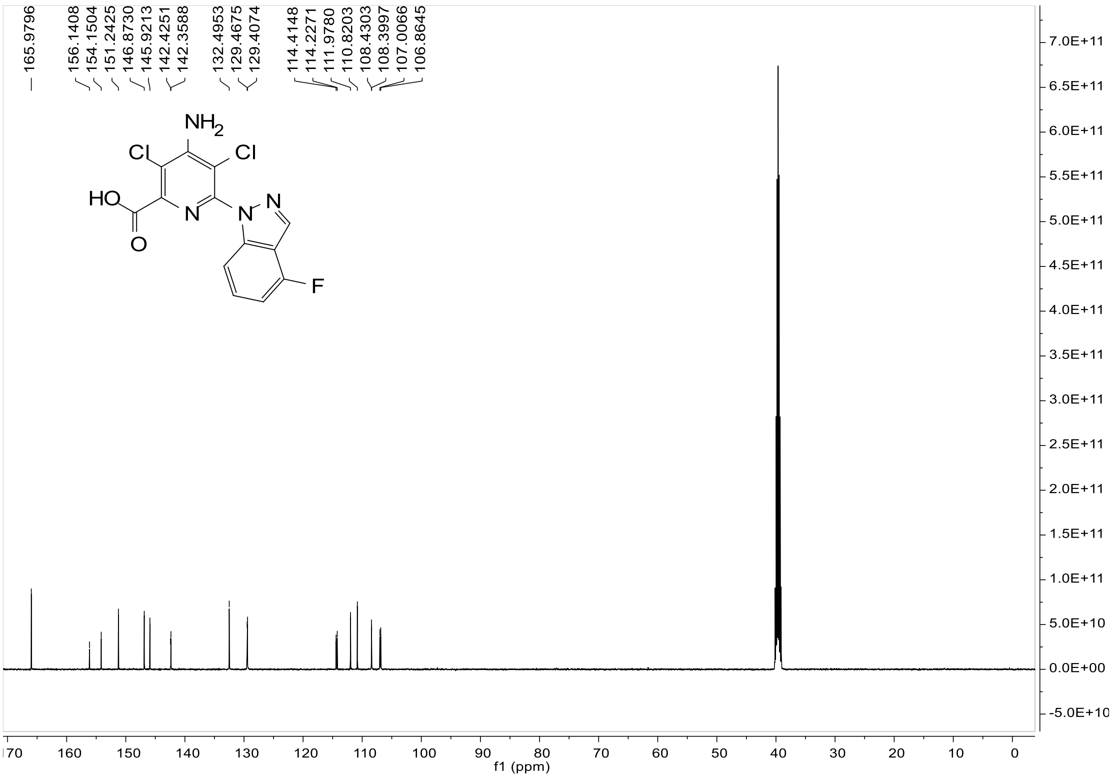

165.9796
156.1408
154.1504
151.2425
146.8730
145.9213
142.4251
142.3588
132.4953
129.4675
129.4074
114.4148
114.2271
111.9780
110.8203
108.4303
108.3997
107.0066
106.8645
NH2
Cl
Cl
HO
N
N
N
O
F
f1 (ppm)

Compound 5a

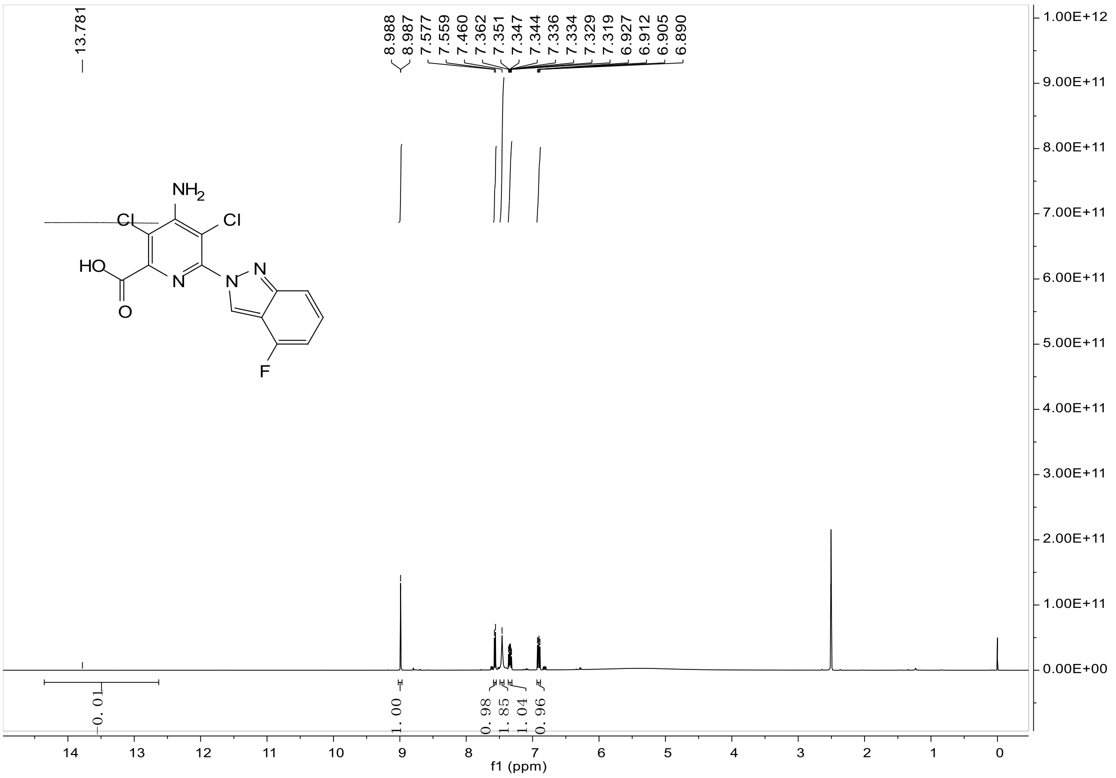
13.781
8.988
8.987
7.577
7.559
7.460
7.362
7.351
7.347
7.344
7.336
7.334
7.329
7.319
6.927
6.912
6.905
6.890
0.01
1.00
0.98
1.85
1.04
0.96
f1 (ppm)

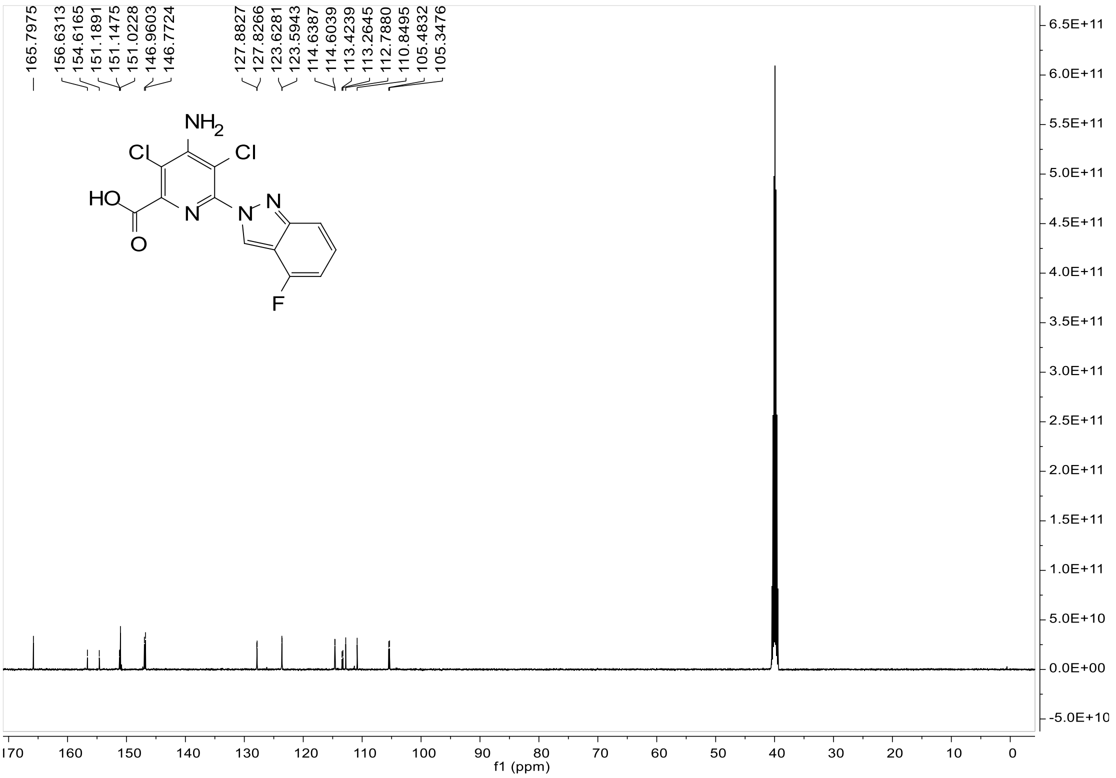
165.7975
156.6313
154.6165
151.1891
151.1475
151.0228
146.9603
146.7724
127.8827
127.8266
123.6281
123.5943
114.6387
114.6039
113.4239
113.2645
112.7880
110.8495
105.4832
105.3476
f1 (ppm)

Compound 5B

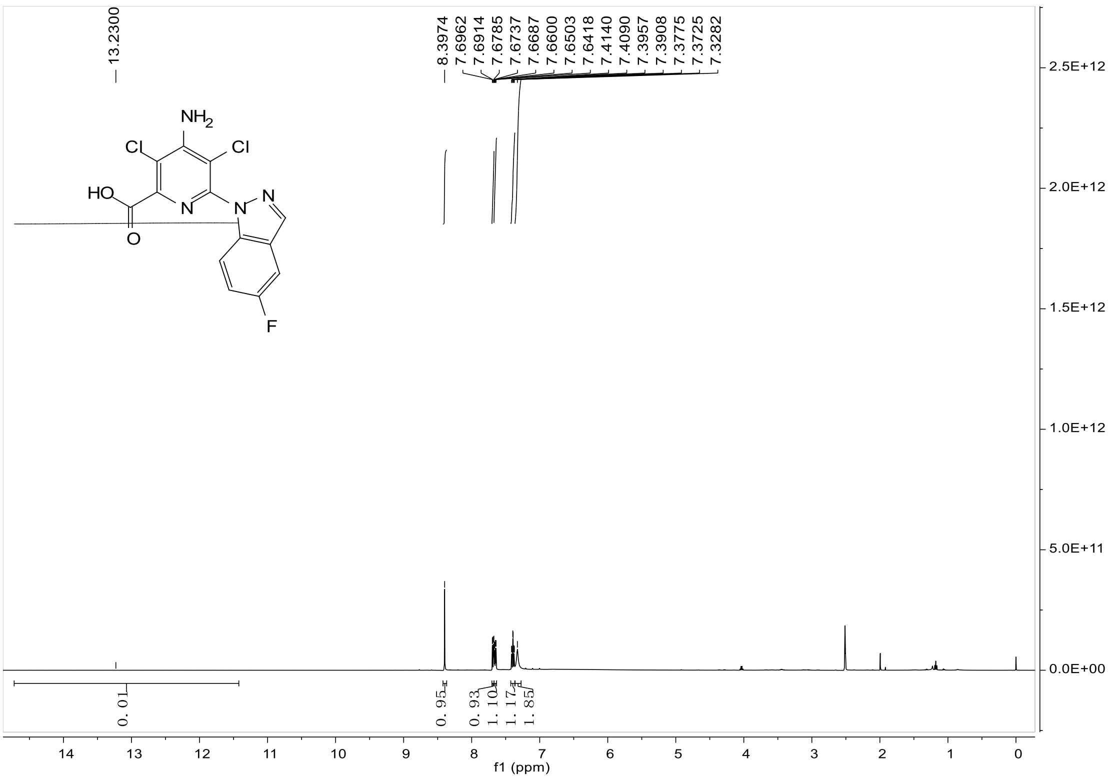
13.2300
8.3974
7.6962
7.6914
7.6785
7.6737
7.6687
7.6600
7.6503
7.6418
7.4140
7.4090
7.3957
7.3908
7.3775
7.3725
7.3282
NH2
Cl
Cl
HO
N
N
N
O
F
0.01
0.95
0.93
1.10
1.17
1.85
2.5E+12
2.0E+12
1.5E+12
1.0E+12
5.0E+11
0.0E+00
14
13
12
11
10
9
8
7
6
5
4
3
2
1
0
f1 (ppm)

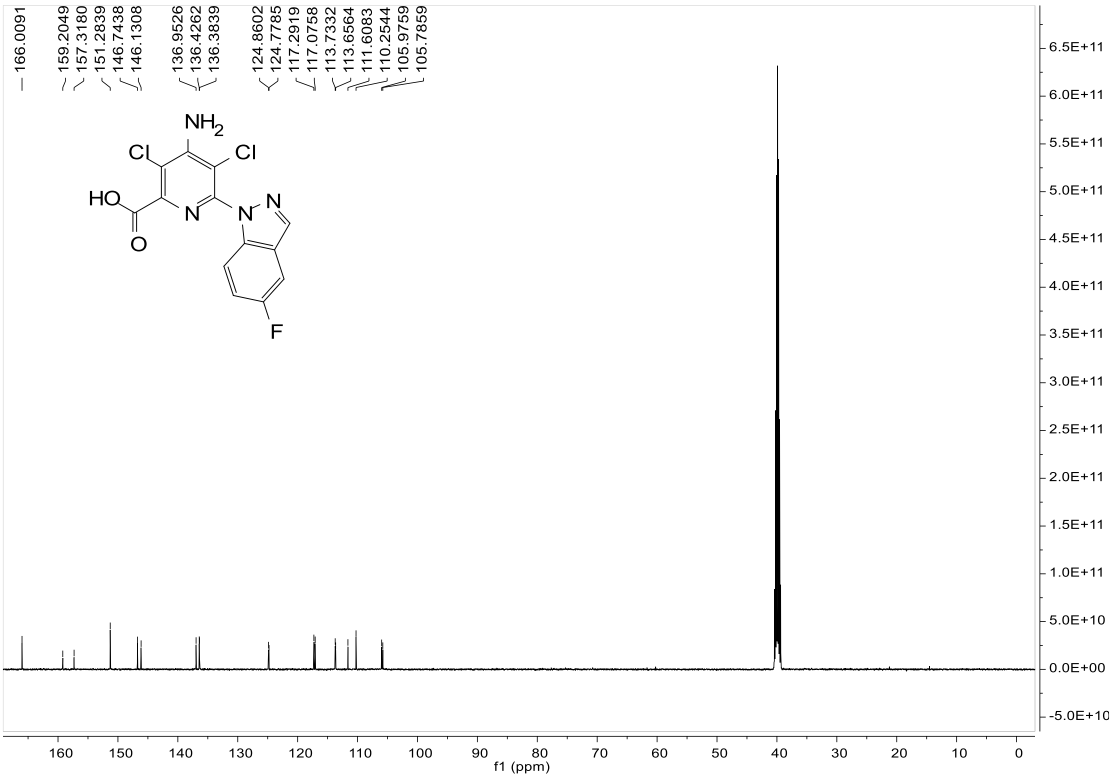
166.0091
159.2049
157.3180
151.2839
146.7438
146.1308
136.9526
136.4262
136.3839
124.8602
124.7785
117.2919
117.0758
113.7332
113.6564
111.6083
110.2544
105.9759
105.7859
NH2
Cl
Cl
HO
N
N
N
O
F
6.5E+11
6.0E+11
5.5E+11
5.0E+11
4.5E+11
4.0E+11
3.5E+11
3.0E+11
2.5E+11
2.0E+11
1.5E+11
1.0E+11
5.0E+10
0.0E+00
-5.0E+10
160
150
140
130
120
110
100
90
80
70
60
50
40
30
20
10
0
f1 (ppm)

Compound 5b

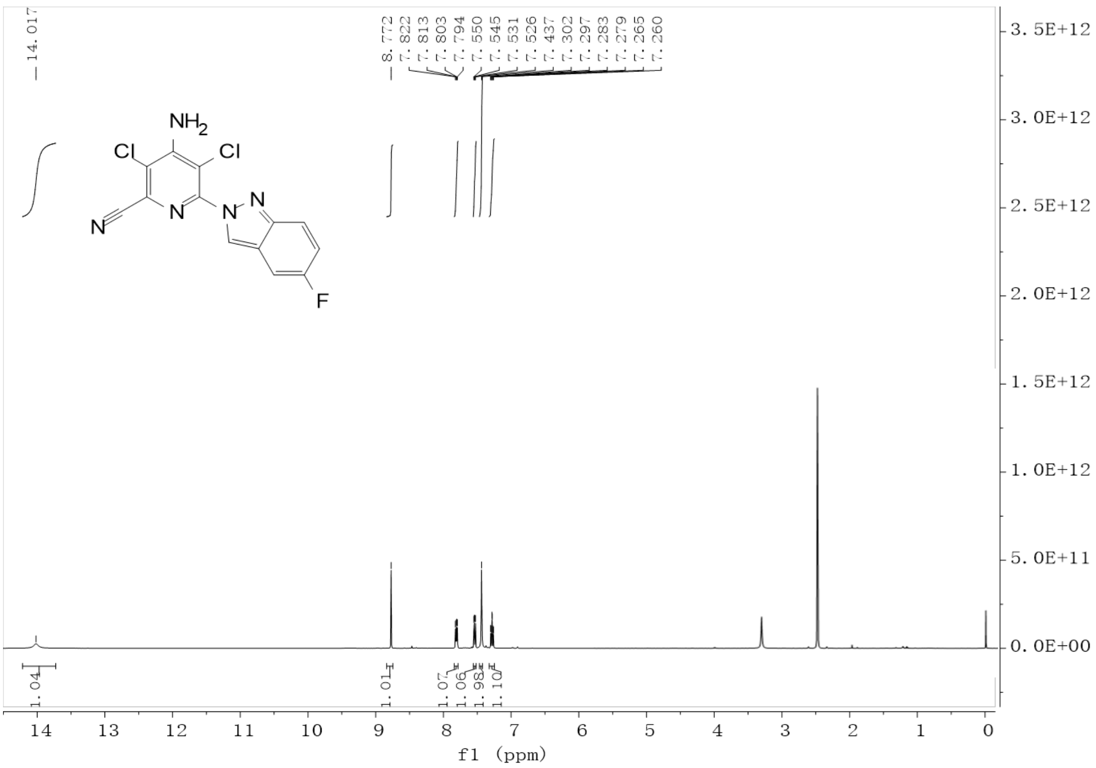
14.017
8.772
7.822
7.813
7.803
7.794
7.550
7.545
7.531
7.526
7.437
7.302
7.297
7.283
7.279
7.265
7.260
NH2
Cl
Cl
N
N
N
N
F
1.04
1.01
1.07
1.06
1.98
1.10
f1 (ppm)

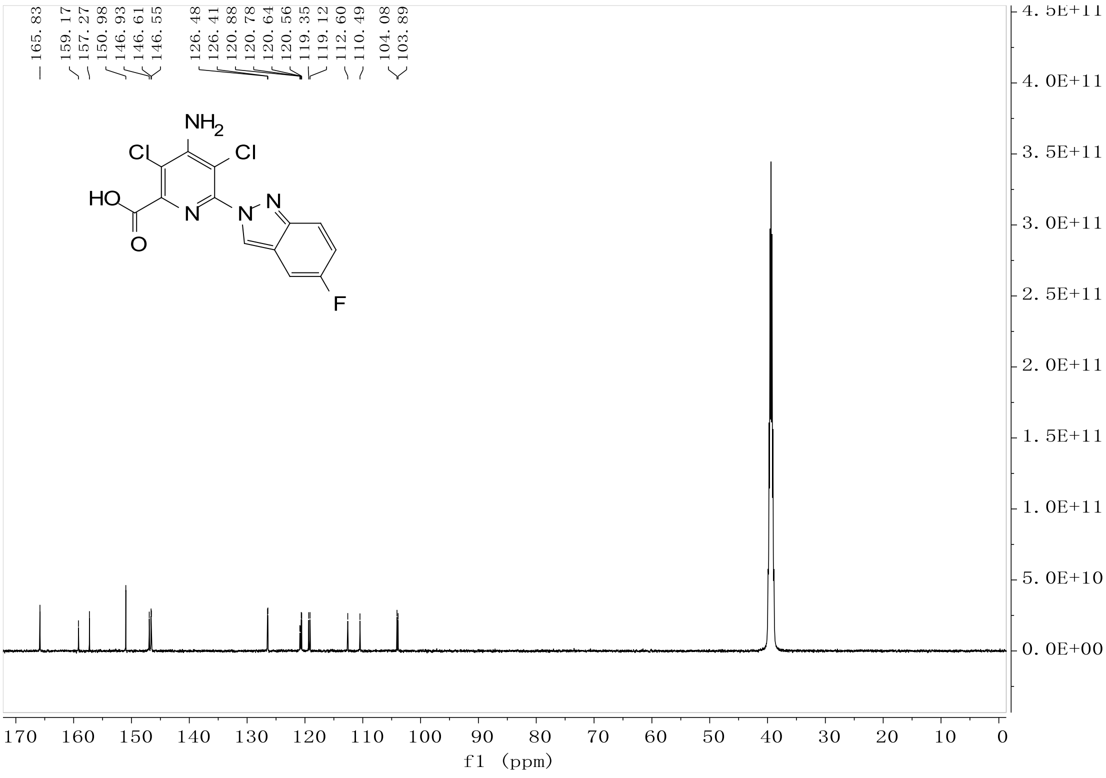
165.83
159.17
157.27
150.98
146.93
146.61
146.55
126.48
126.41
120.88
120.78
120.64
120.56
119.35
119.12
112.60
110.49
104.08
103.89
NH2
Cl
Cl
HO
N
N
N
O
F
f1 (ppm)

Compound 5Cc

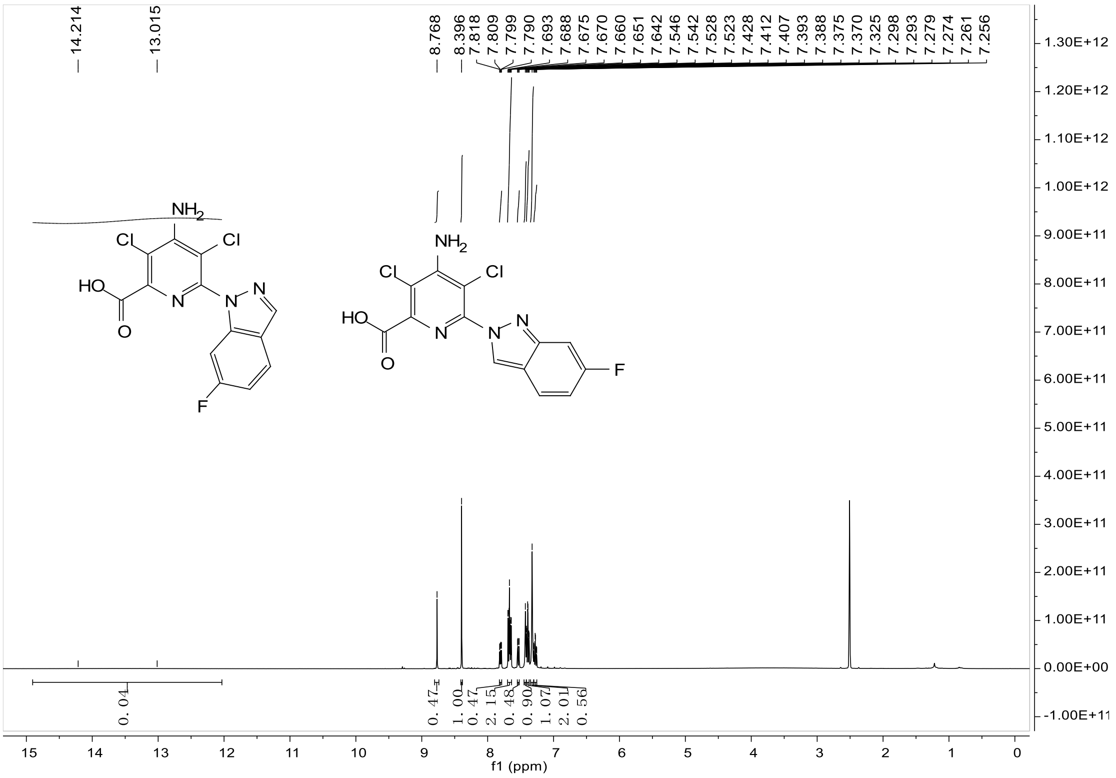

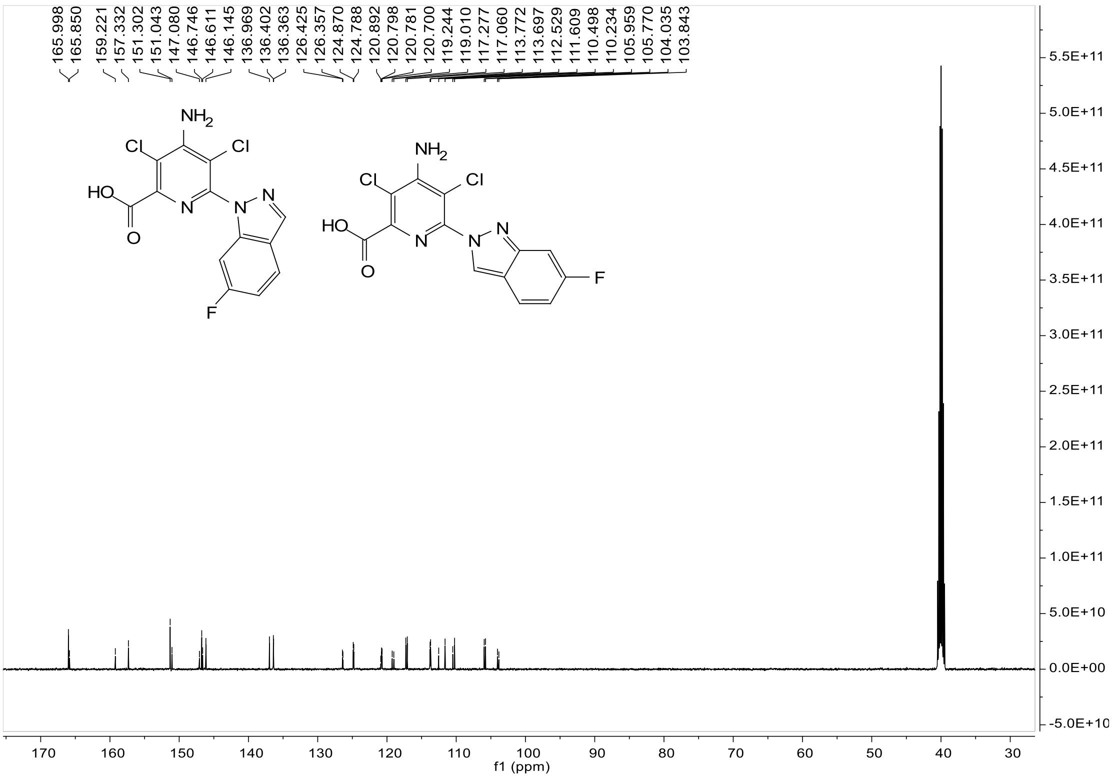

Compound 5d

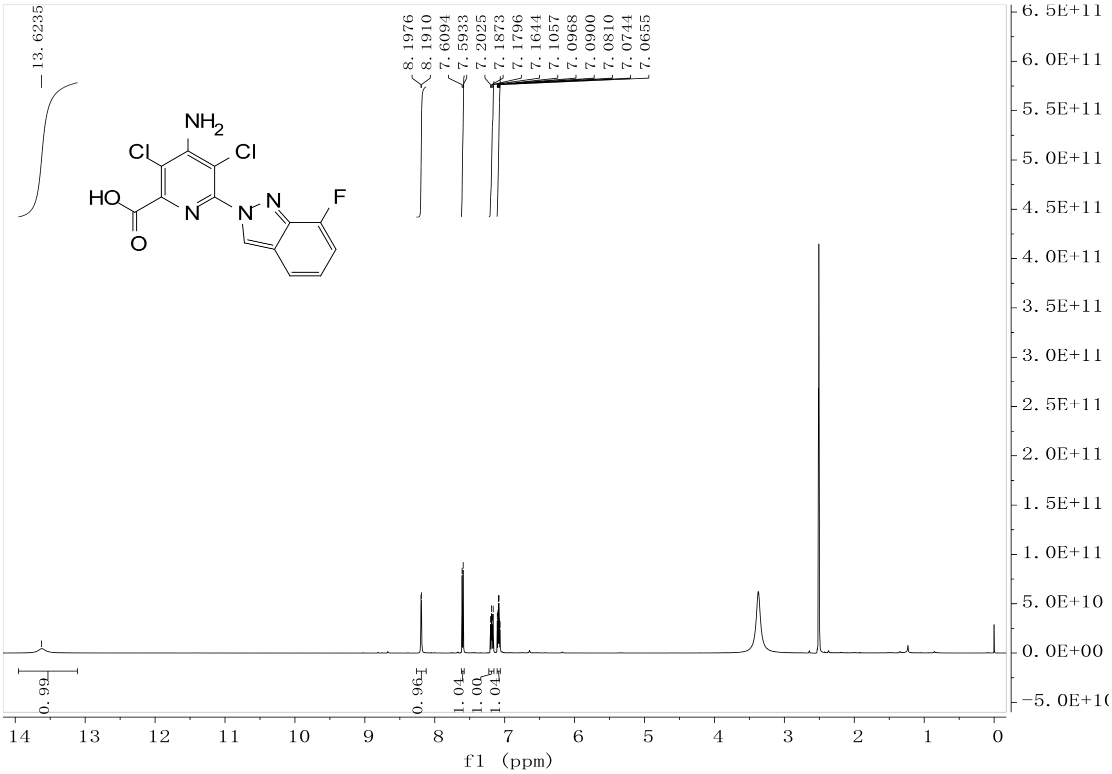
13.6235
8.1976
8.1910
7.6094
7.5933
7.2025
7.1873
7.1796
7.1644
7.1057
7.0968
7.0900
7.0810
7.0744
7.0655
NH2
Cl
Cl
HO
N
N
N
F
O
0.99
0.96
1.04
1.00
1.04
f1 (ppm)

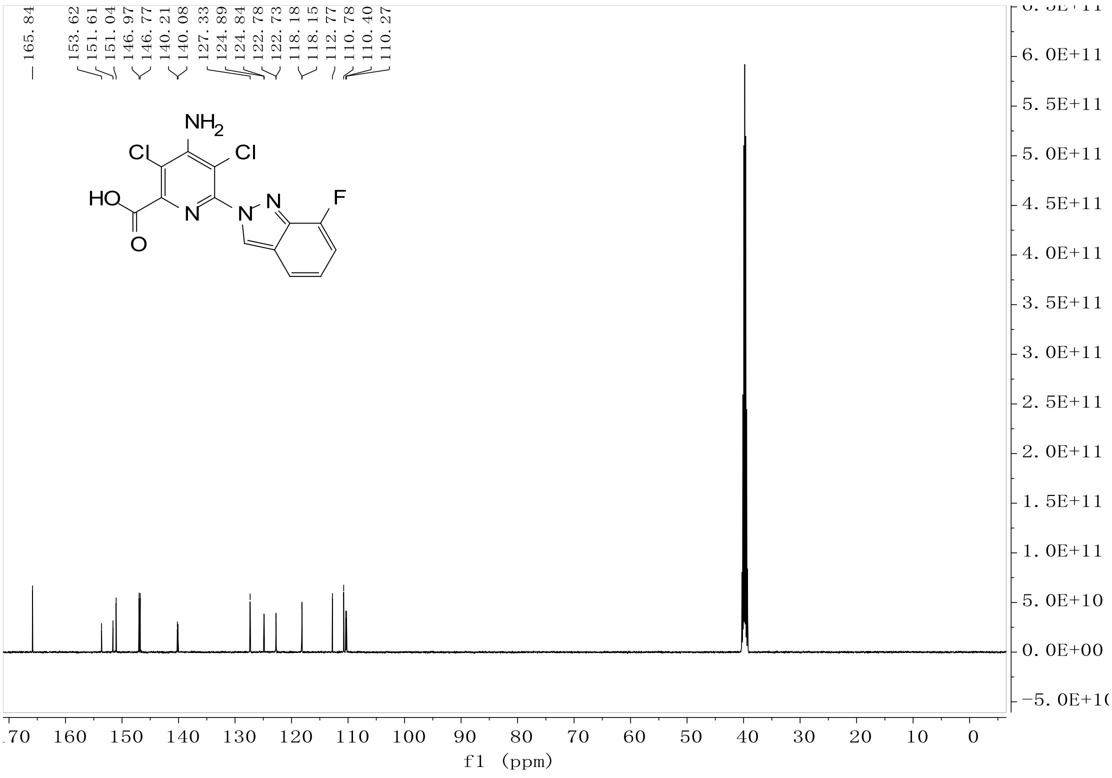
165.84
153.62
151.61
151.04
146.97
146.77
140.21
140.08
127.33
124.89
124.84
122.78
122.73
118.18
118.15
112.77
110.78
110.40
110.27
NH2
Cl
Cl
HO
N
N
N
F
O
f1 (ppm)

Compound 6A

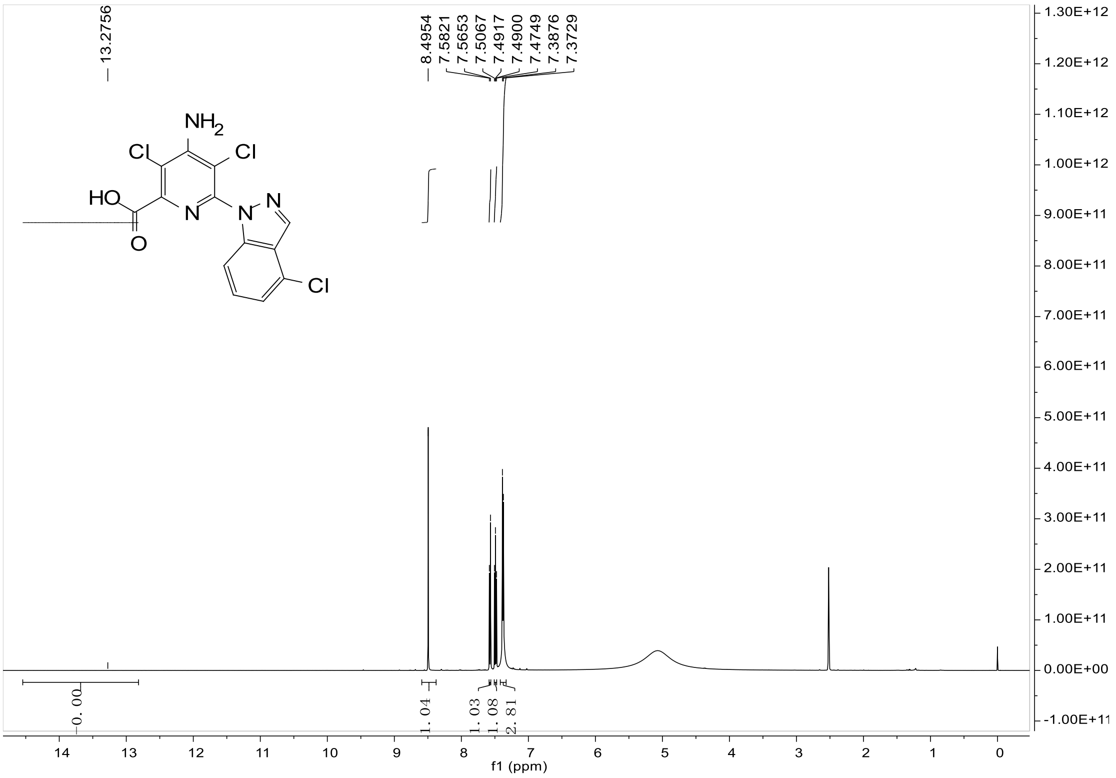

13.2756
8.4954
7.5821
7.5653
7.5067
7.4917
7.4900
7.4749
7.3876
7.3729
NH2
Cl
Cl
HO
N
N
N
O
Cl
0.00
1.04
1.03
1.08
2.81
f1 (ppm)

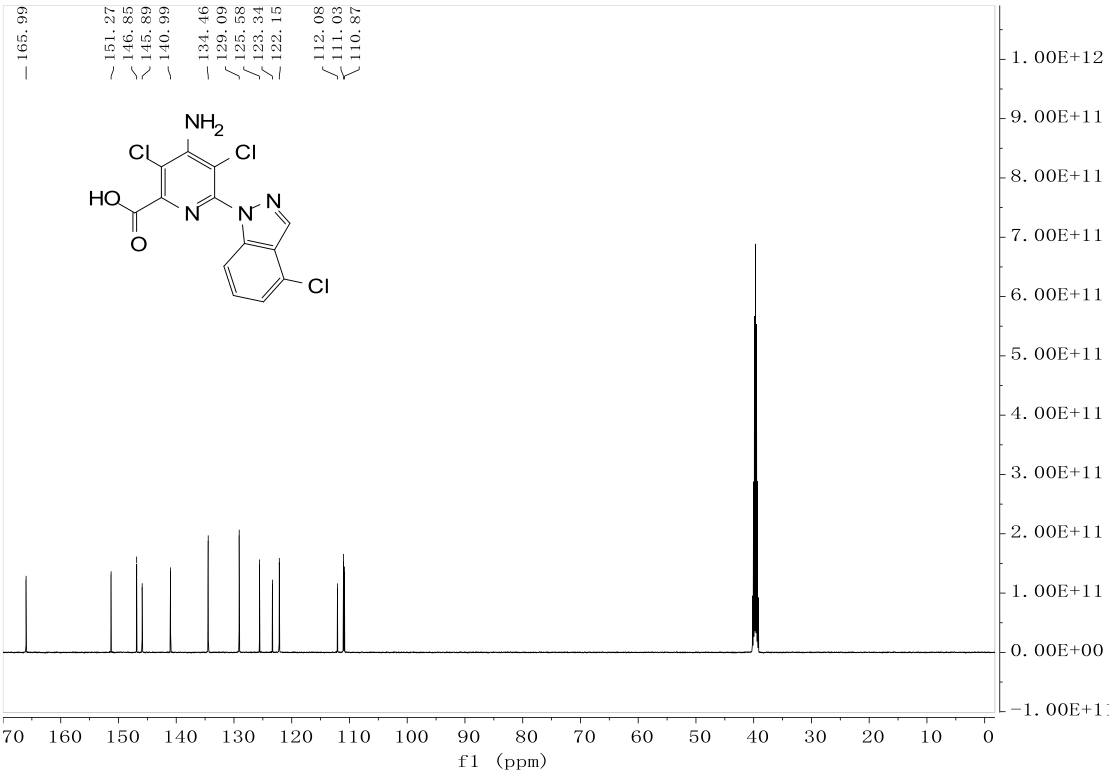

165.99
151.27
146.85
145.89
140.99
134.46
129.09
125.58
123.34
122.15
112.08
111.03
110.87
NH2
Cl
Cl
HO
N
N
N
O
Cl
f1 (ppm)

Compound 6a

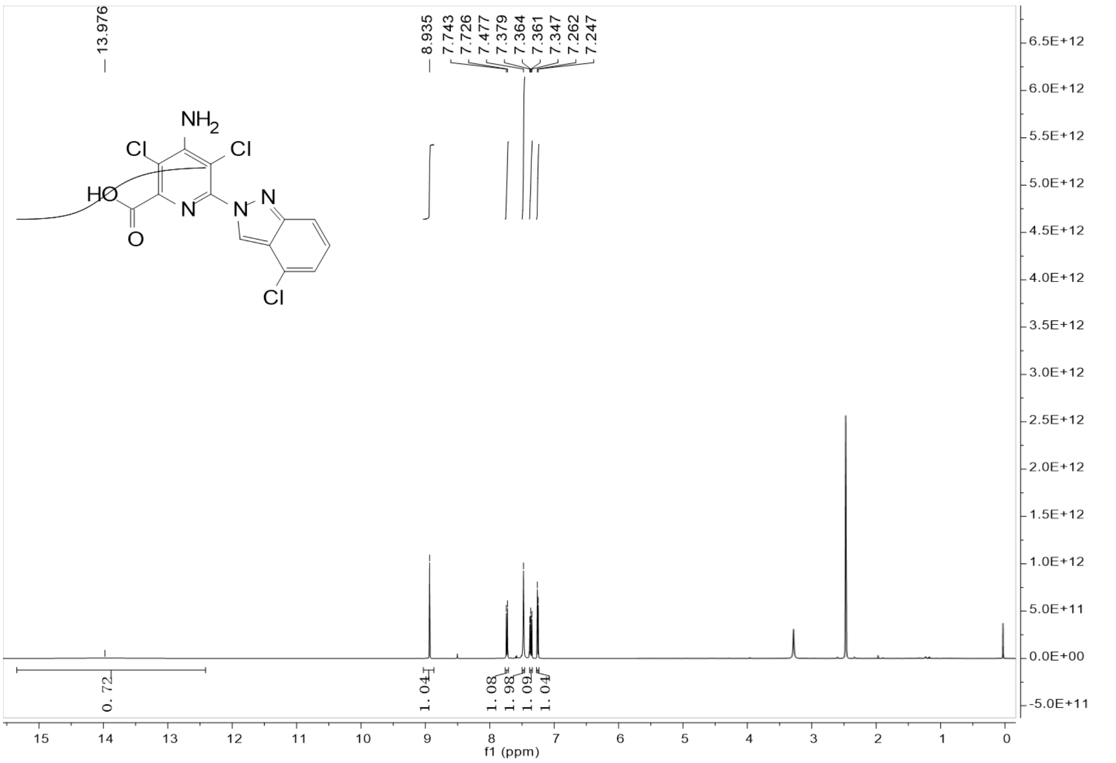
13.976
8.935
7.743
7.726
7.477
7.379
7.364
7.361
7.347
7.262
7.247
NH2
Cl
Cl
HO
N
N
N
O
Cl
0.72
1.04
1.08
1.98
1.09
1.04
f1 (ppm)

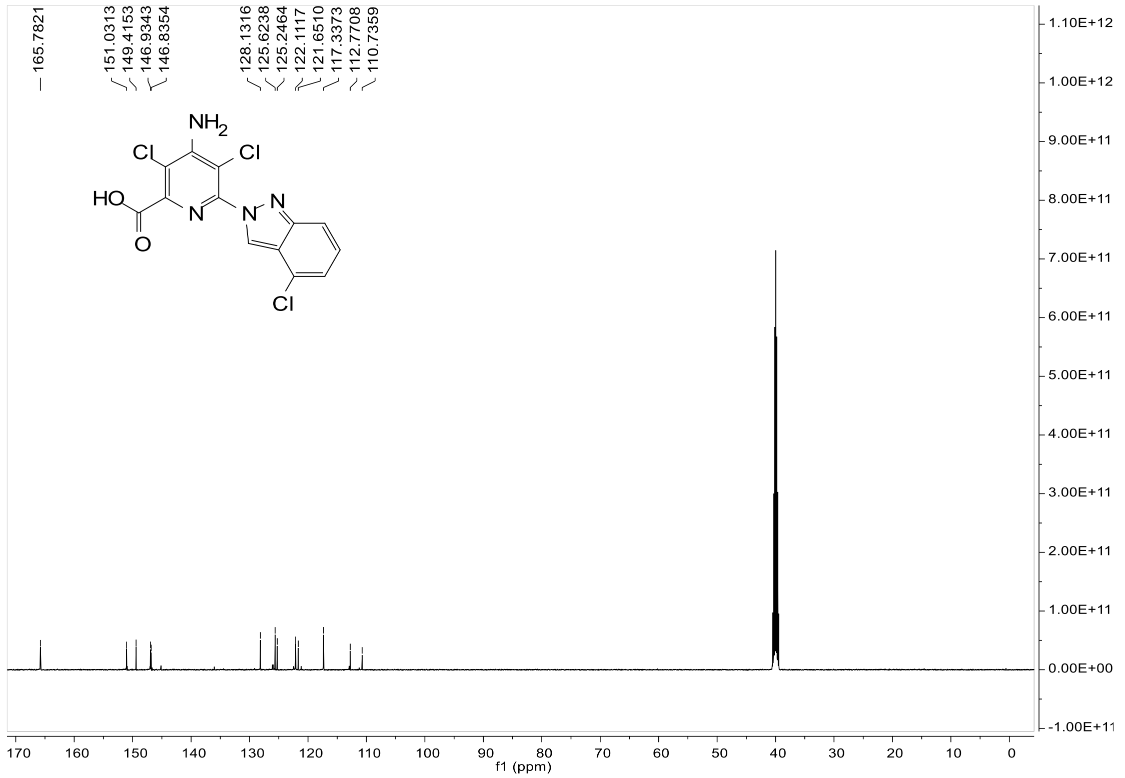
165.7821
151.0313
149.4153
146.9343
146.8354
128.1316
125.6238
125.2464
122.1117
121.6510
117.3373
112.7708
110.7359
NH2
Cl
Cl
HO
N
N
N
O
Cl
f1 (ppm)

Compound 6B

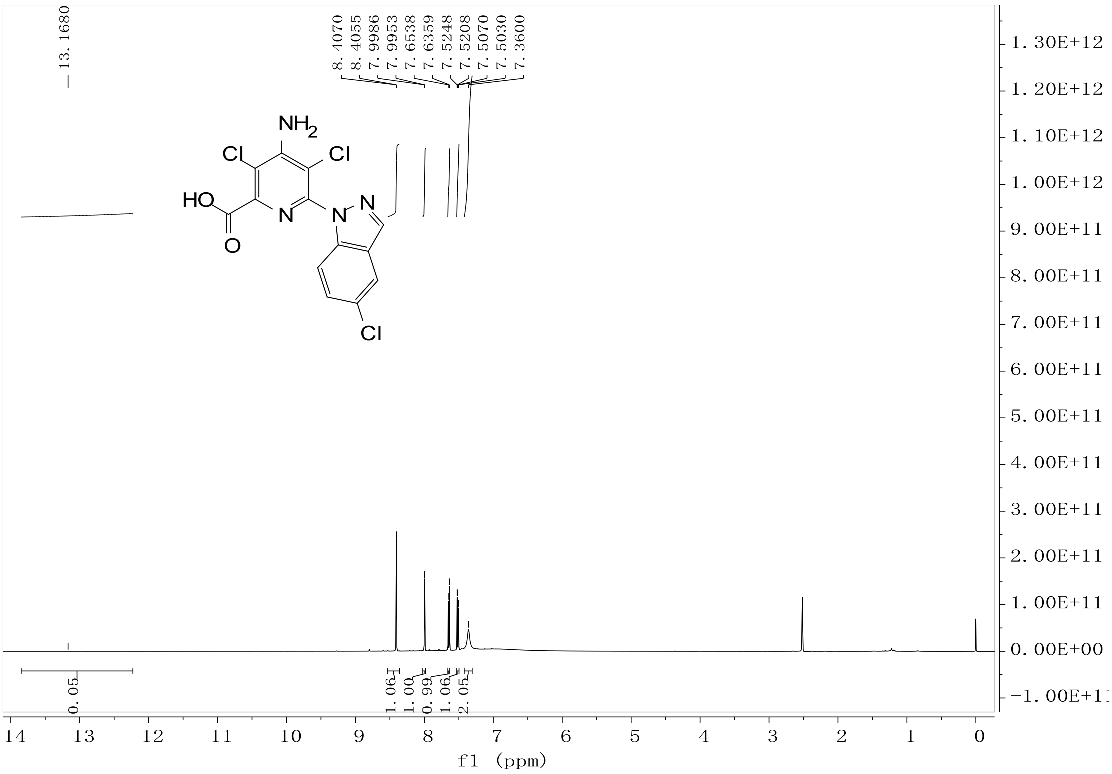

13.1680
8.4070
8.4055
7.9986
7.9953
7.6538
7.6359
7.5248
7.5208
7.5070
7.5030
7.3600
NH2
Cl
Cl
HO
N
N
N
O
Cl
0.05
1.06
1.00
0.99
1.06
2.05
f1 (ppm)

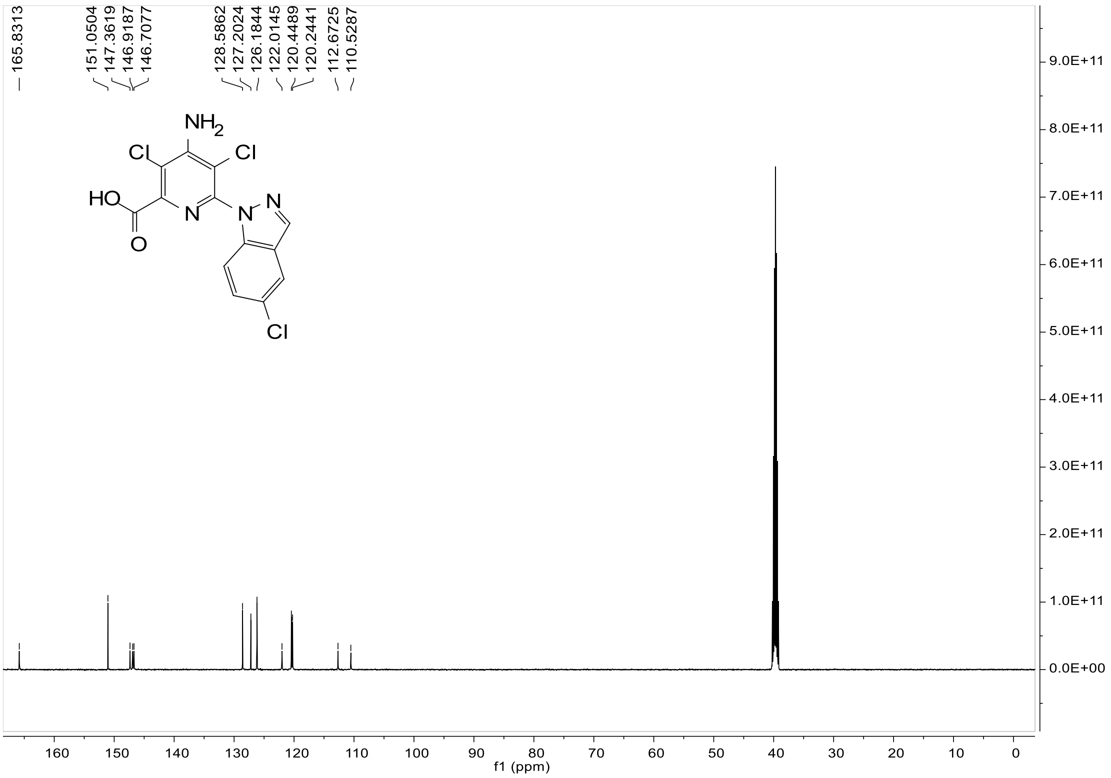

165.8313
151.0504
147.3619
146.9187
146.7077
128.5862
127.2024
126.1844
122.0145
120.4489
120.2441
112.6725
110.5287
NH2
Cl
Cl
HO
N
N
N
O
Cl
f1 (ppm)

Compound 6b

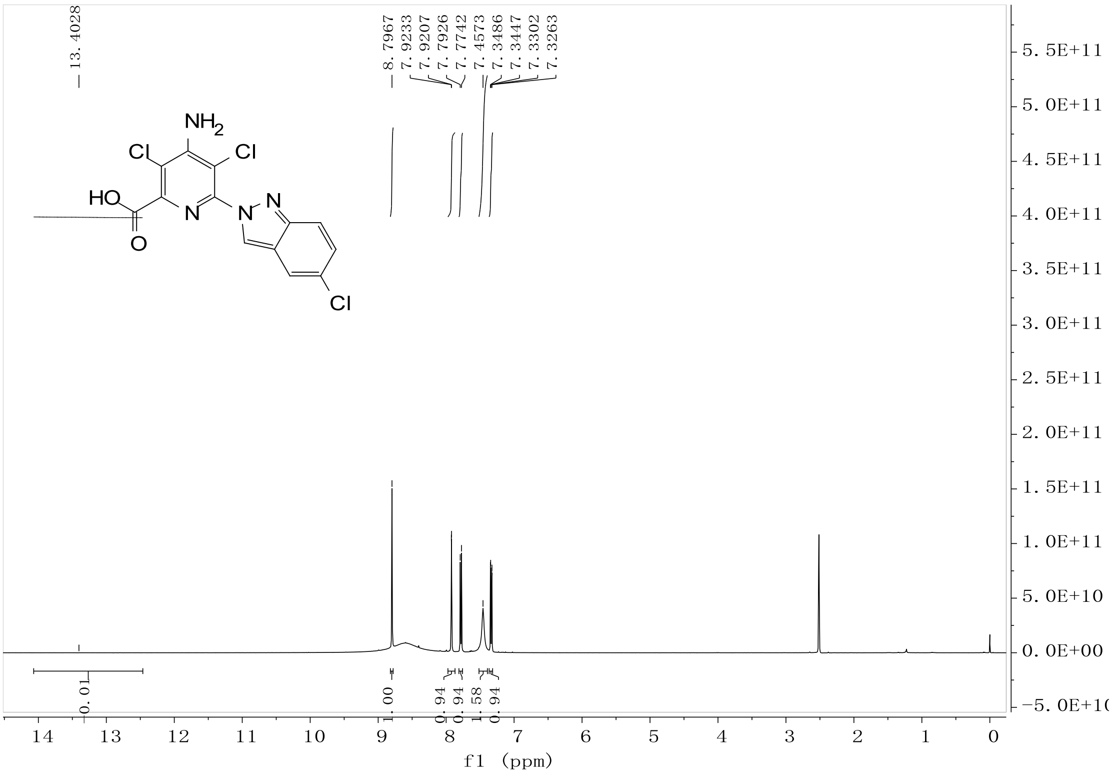
13.4028
8.7967
7.9233
7.9207
7.7926
7.7742
7.4573
7.3486
7.3447
7.3302
7.3263
NH2
Cl
Cl
HO
N
N
N
O
Cl
0.01
1.00
0.94
0.94
1.58
0.94
f1 (ppm)

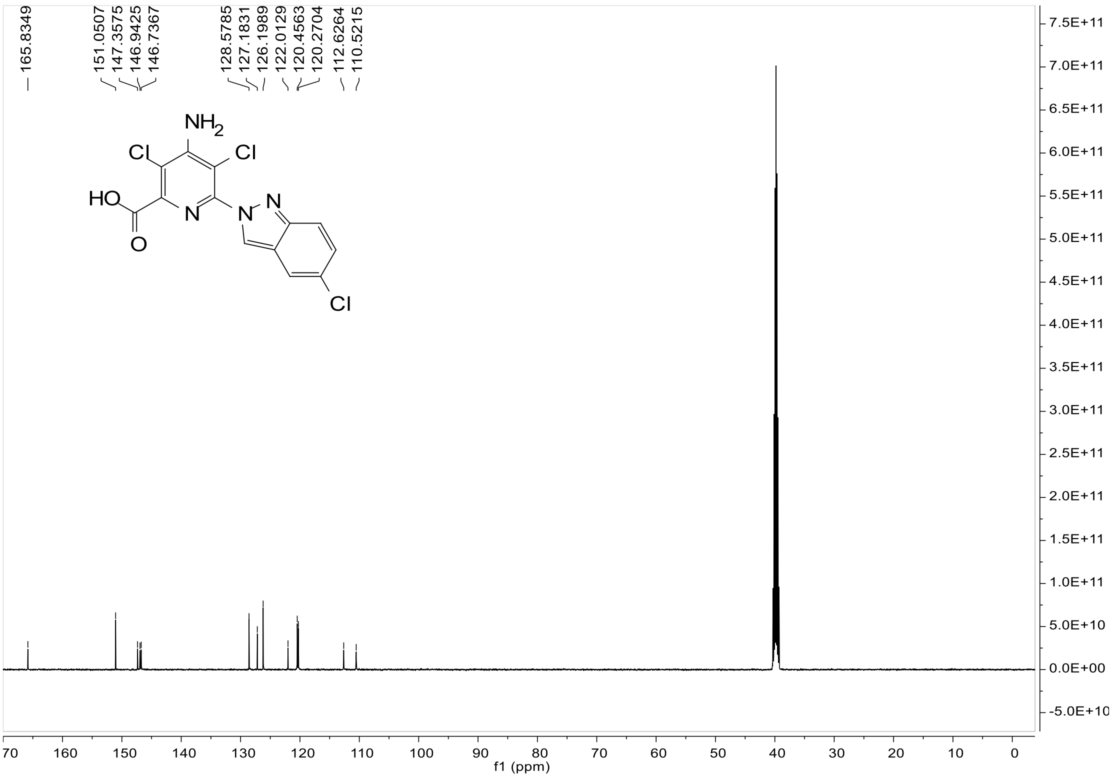
165.8349
151.0507
147.3575
146.9425
146.7367
128.5785
127.1831
126.1989
122.0129
120.4563
120.2704
112.6264
110.5215
NH2
Cl
Cl
HO
N
N
N
O
Cl
f1 (ppm)

Compound 6Cc

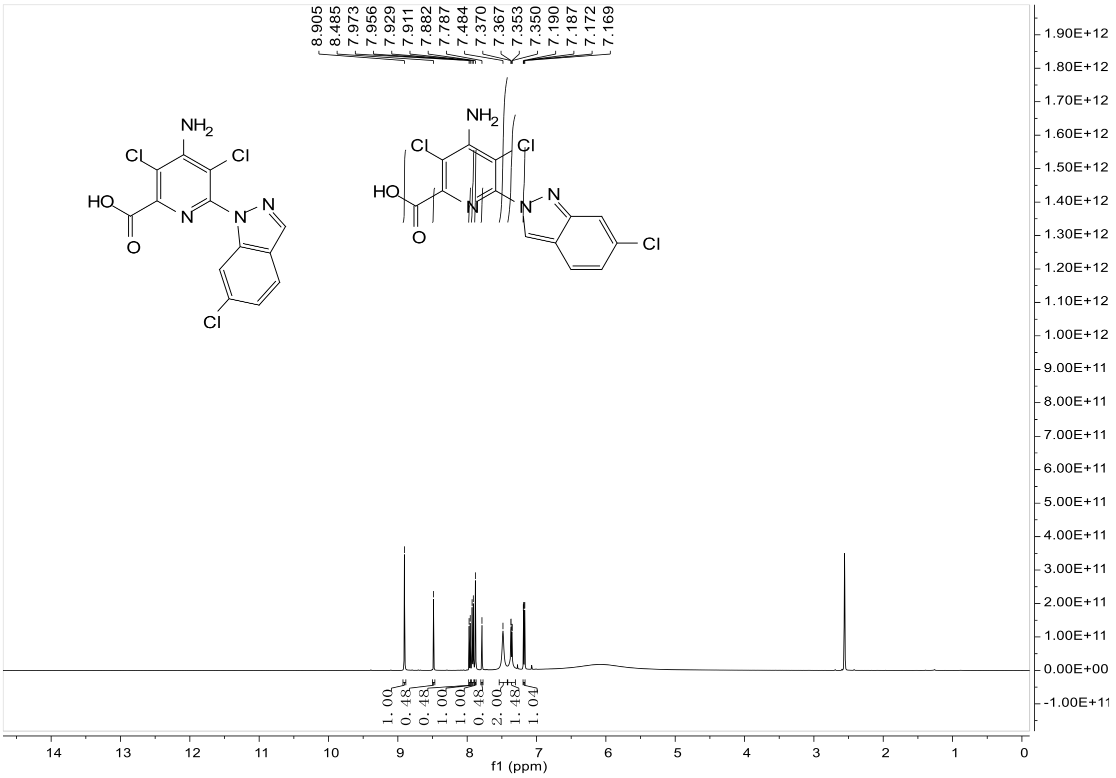

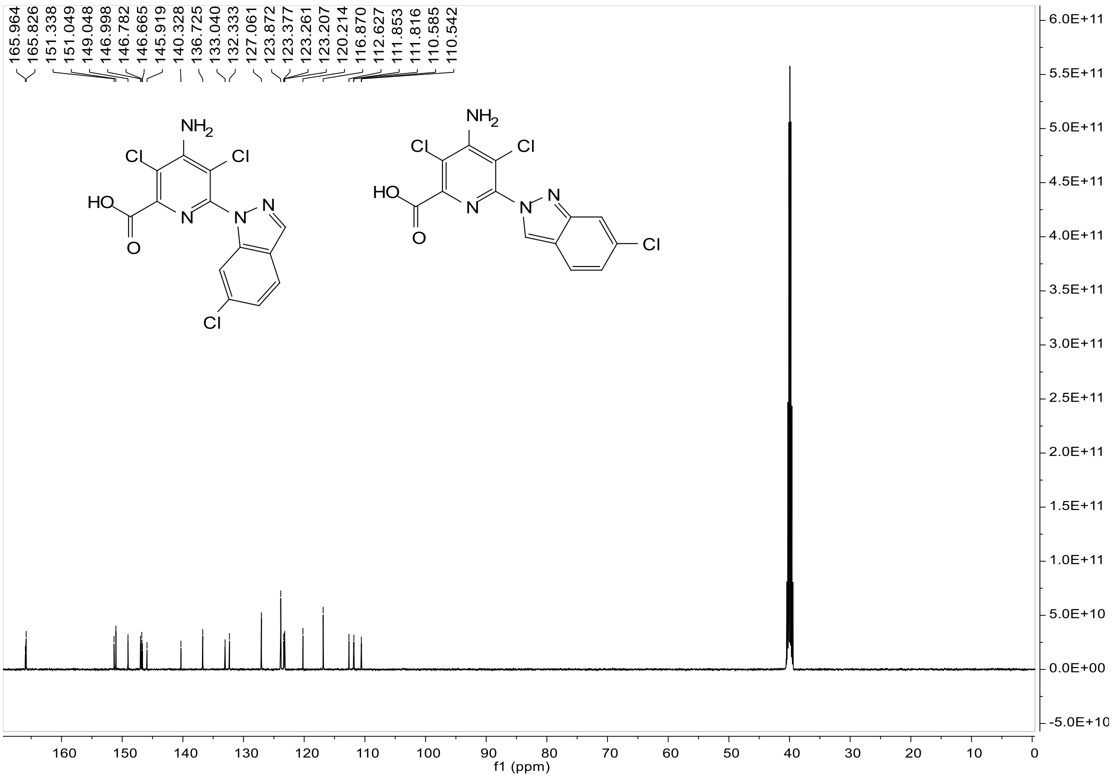

Compound 6d

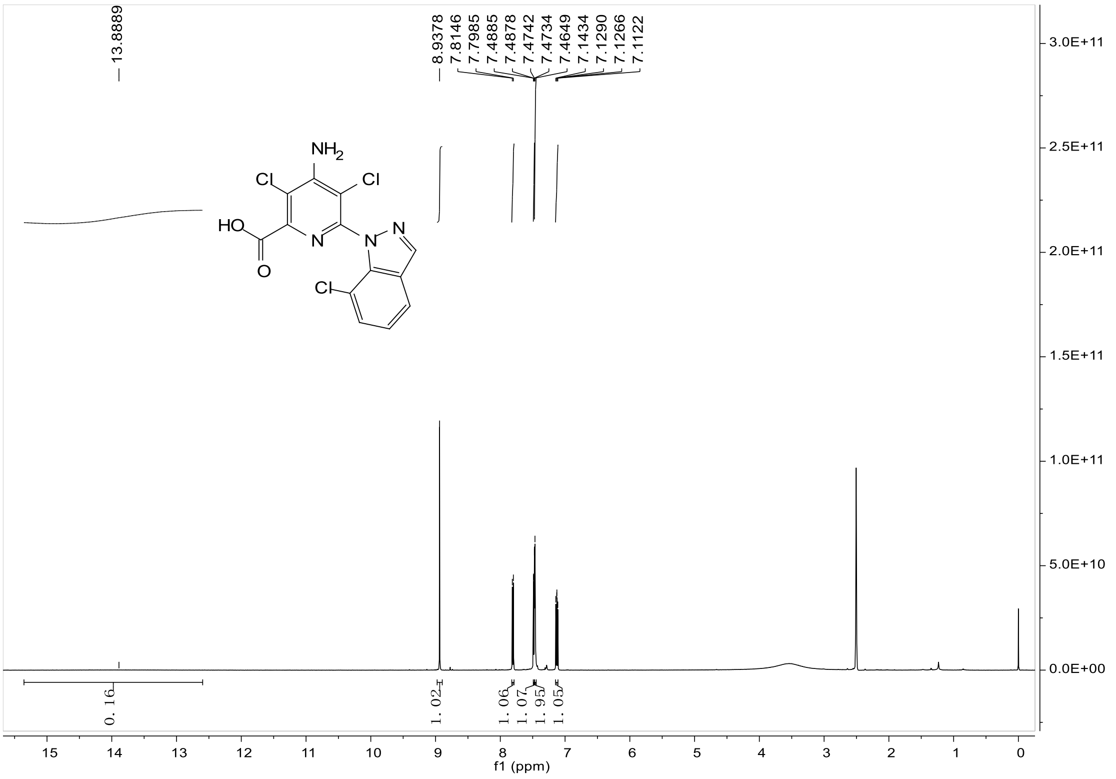

13.8889
8.9378
7.8146
7.7985
7.4885
7.4878
7.4742
7.4734
7.4649
7.1434
7.1290
7.1266
7.1122
NH2
Cl
Cl
HO
N
N
N
O
Cl
0.16
1.02
1.06
1.07
1.95
1.05
f1 (ppm)

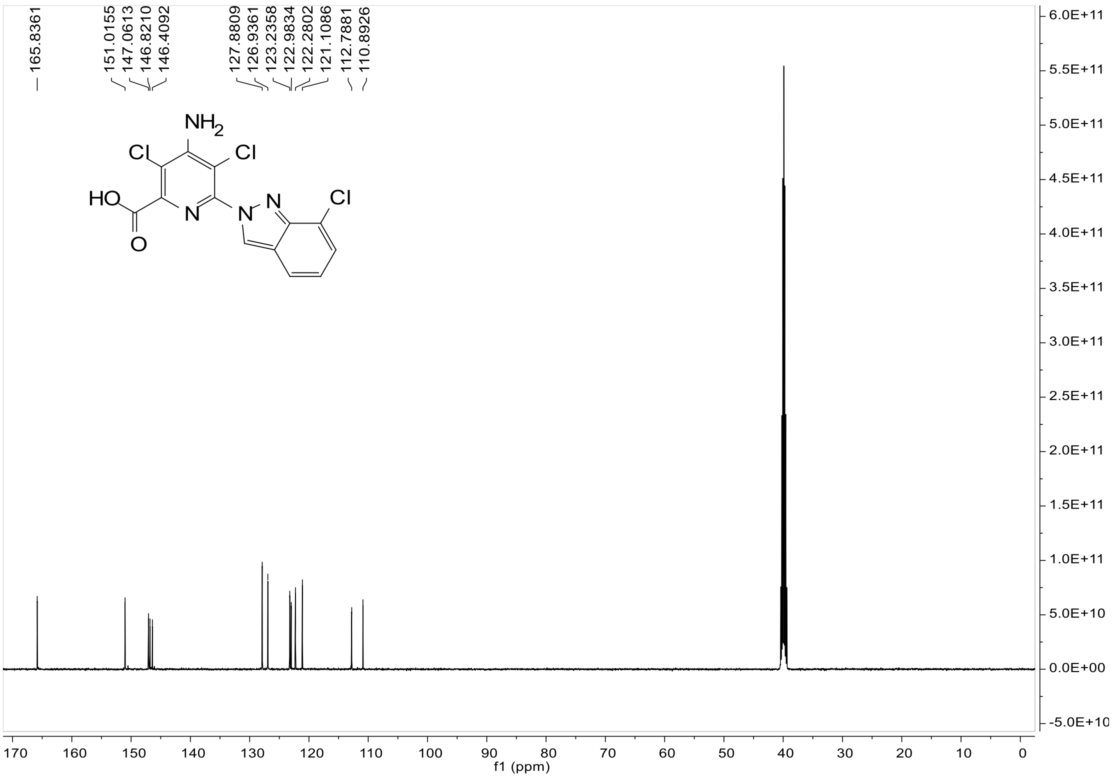

165.8361
151.0155
147.0613
146.8210
146.4092
127.8809
126.9361
123.2358
122.9834
122.2802
121.1086
112.7881
110.8926
NH2
Cl
Cl
HO
N
N
N
Cl
O
f1 (ppm)

Compound 7A

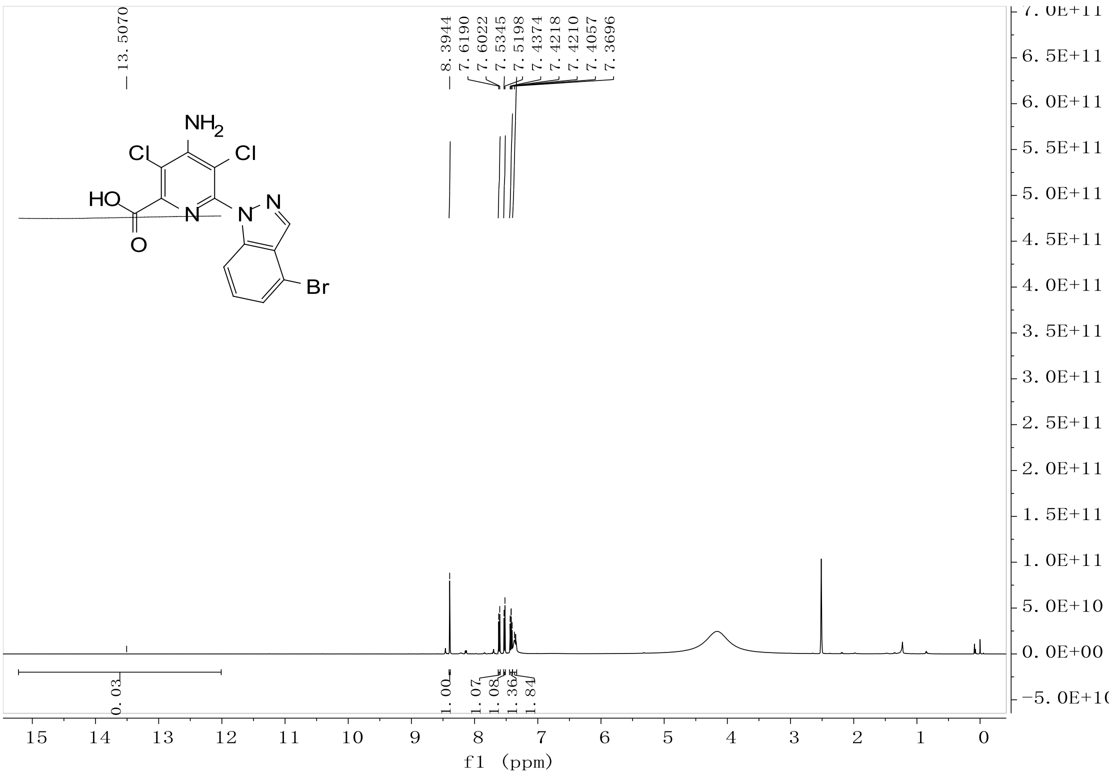

13.5070
8.3944
7.6190
7.6022
7.5345
7.5198
7.4374
7.4218
7.4210
7.4057
7.3696
NH2
Cl
Cl
HO
N
N
N
O
Br
0.03
1.00
1.07
1.08
1.36
1.84
f1 (ppm)

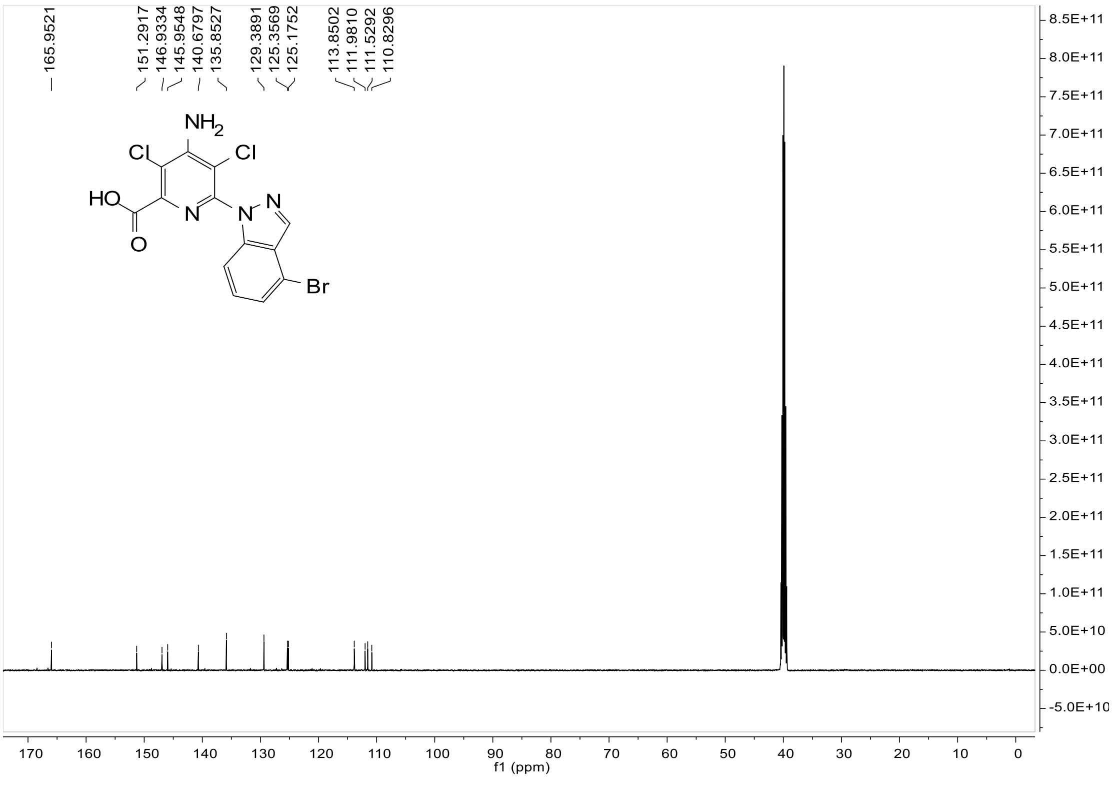

165.9521
151.2917
146.9334
145.9548
140.6797
135.8527
129.3891
125.3569
125.1752
113.8502
111.9810
111.5292
110.8296
NH2
Cl
Cl
HO
N
N
N
O
Br
f1 (ppm)

Compound 7a

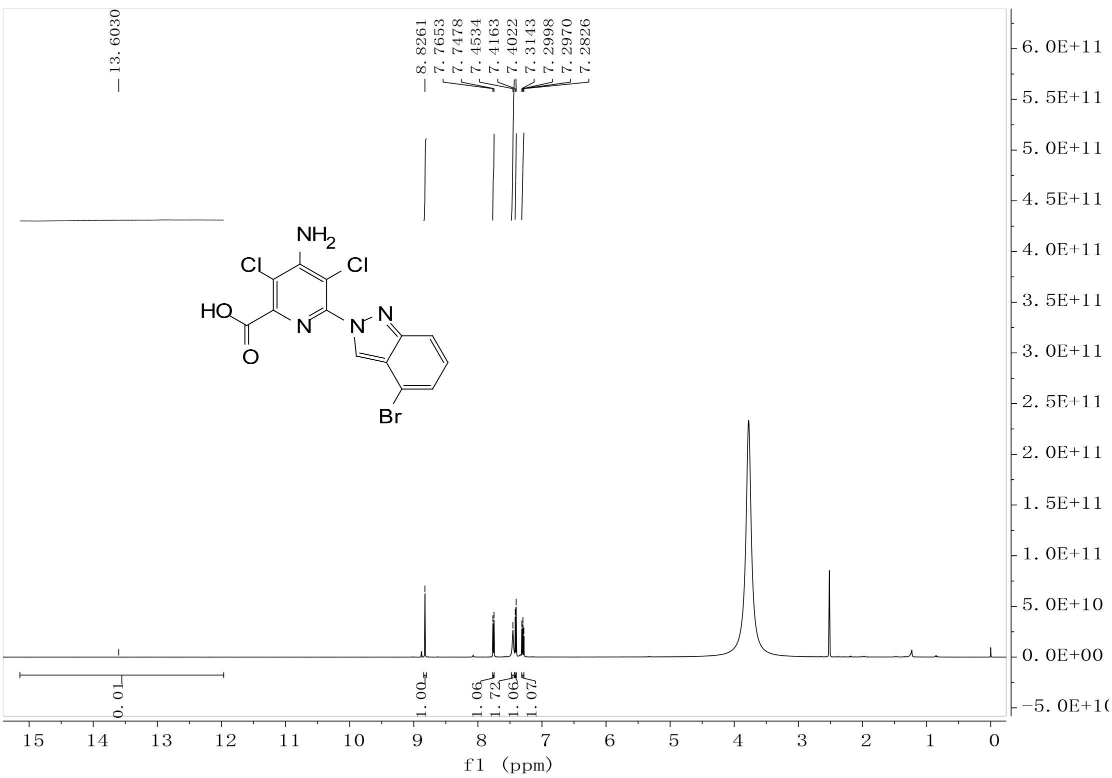
13.6030
8.8261
7.7653
7.7478
7.4534
7.4163
7.4022
7.3143
7.2998
7.2970
7.2826
NH2
Cl
Cl
HO
N
N
N
O
Br
0.01
1.00
1.06
1.72
1.06
1.07
6.0E+11
5.5E+11
5.0E+11
4.5E+11
4.0E+11
3.5E+11
3.0E+11
2.5E+11
2.0E+11
1.5E+11
1.0E+11
5.0E+10
0.0E+00
15 14 13 12 11 10 9 8 7 6 5 4 3 2 1 0
f1 (ppm)

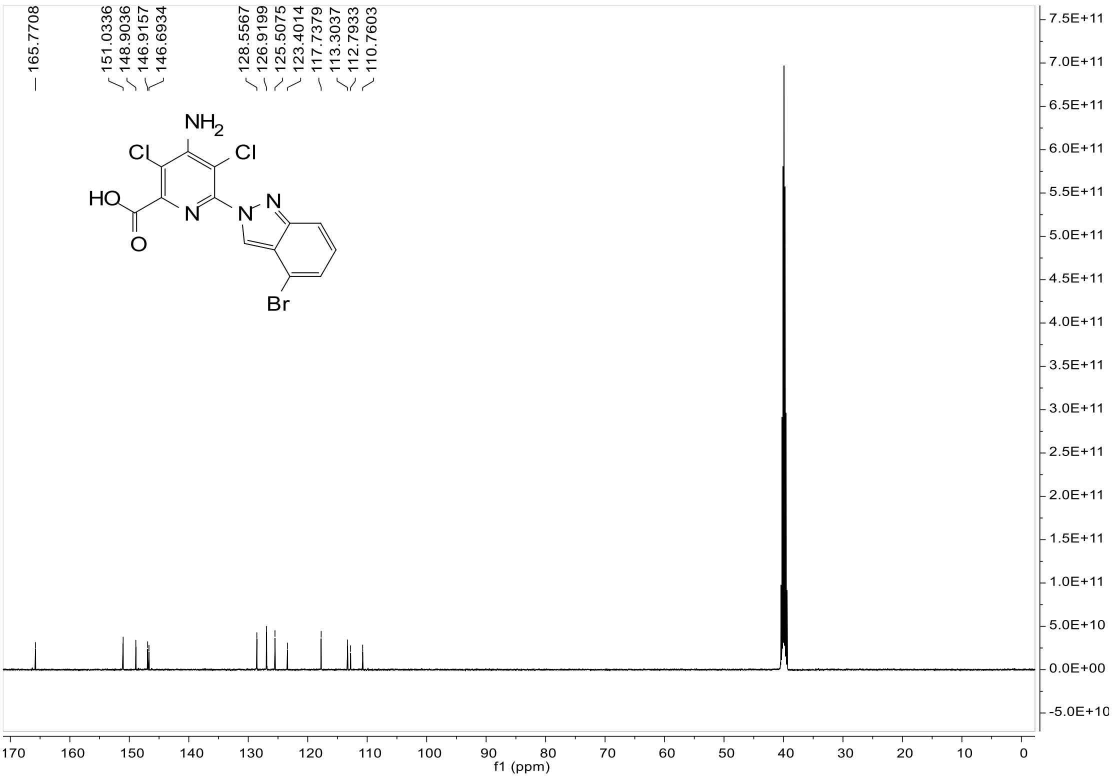
165.7708
151.0336
148.9036
146.9157
146.6934
128.5567
126.9199
125.5075
123.4014
117.7379
113.3037
112.7933
110.7603
NH2
Cl
Cl
HO
N
N
N
O
Br
7.5E+11
7.0E+11
6.5E+11
6.0E+11
5.5E+11
5.0E+11
4.5E+11
4.0E+11
3.5E+11
3.0E+11
2.5E+11
2.0E+11
1.5E+11
1.0E+11
5.0E+10
0.0E+00
-5.0E+10
170 160 150 140 130 120 110 100 90 80 70 60 50 40 30 20 10 0
f1 (ppm)

Compound 7B

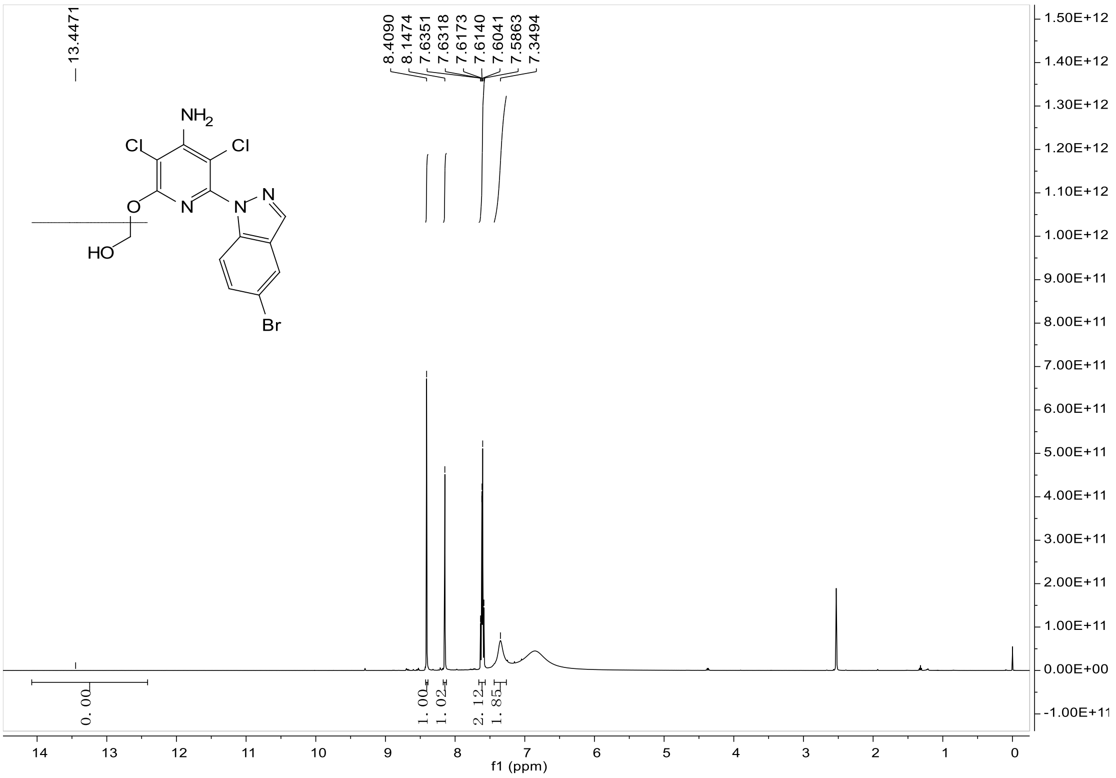

13.4471
8.4090
8.1474
7.6351
7.6318
7.6173
7.6140
7.6041
7.5863
7.3494
NH2
Cl
Cl
O
N
N
N
HO
Br
0.00
1.00
1.02
2.12
1.85
f1 (ppm)

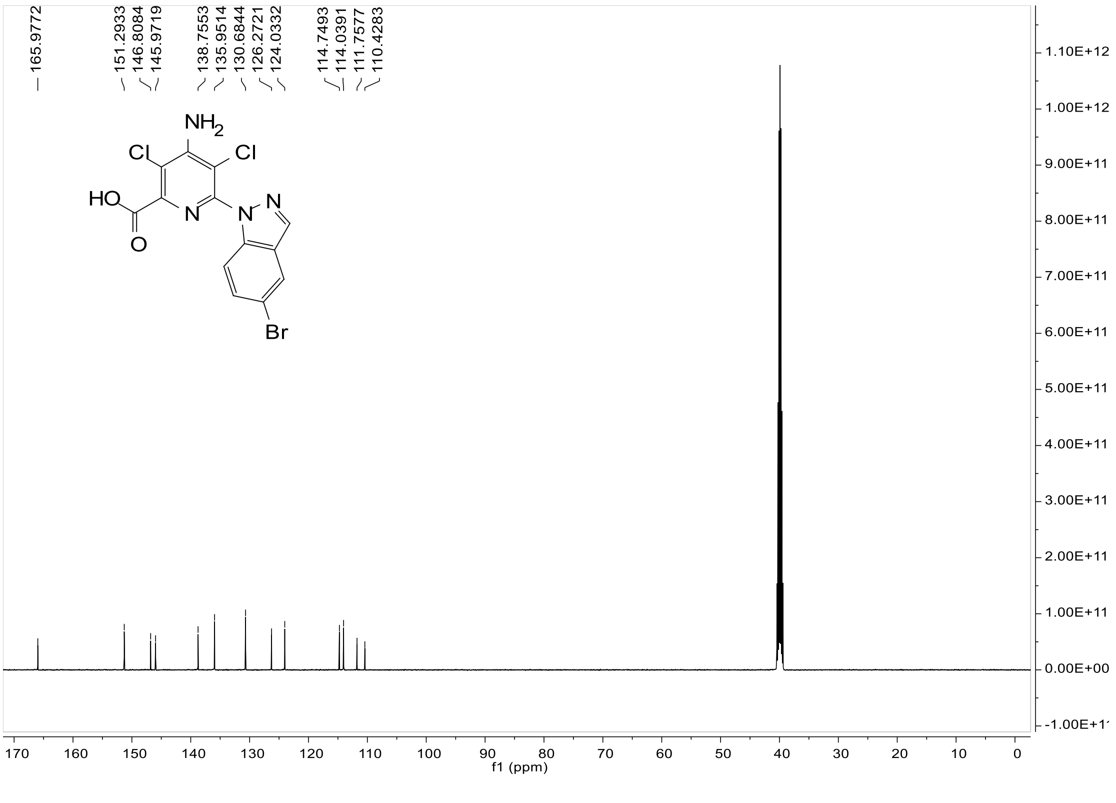

165.9772
151.2933
146.8084
145.9719
138.7553
135.9514
130.6844
126.2721
124.0332
114.7493
114.0391
111.7577
110.4283
NH2
Cl
Cl
HO
N
N
N
O
Br
f1 (ppm)

Compound 7b

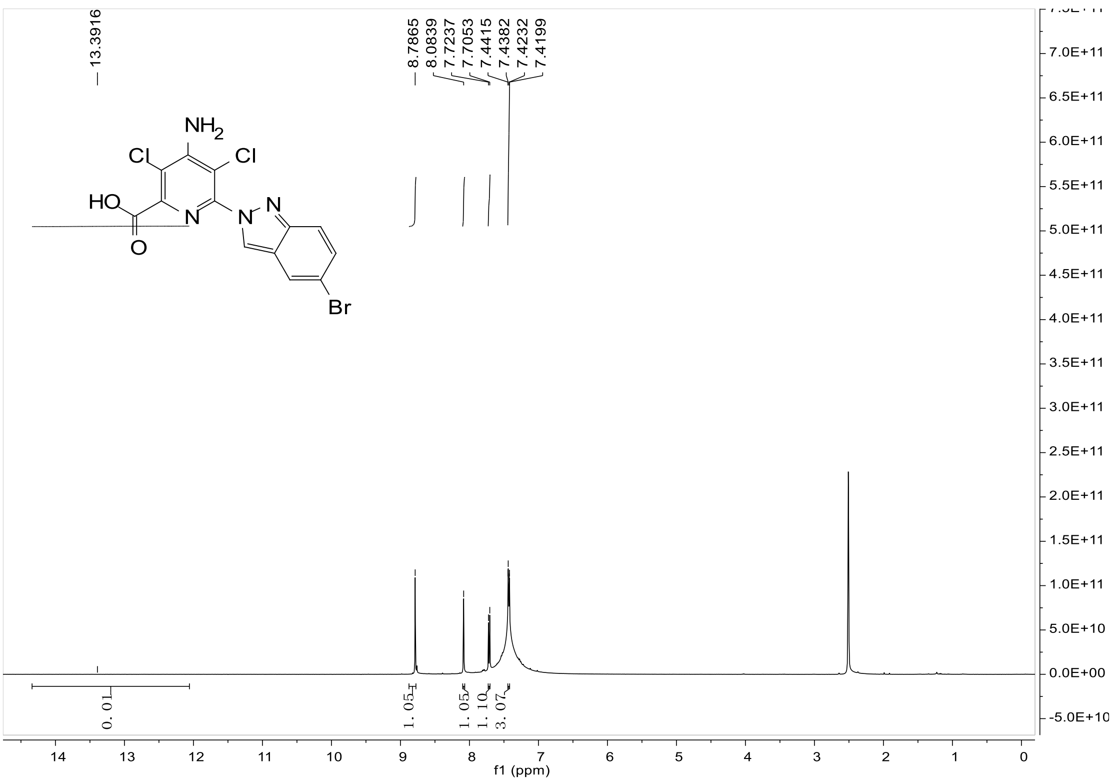
13.3916
8.7865
8.0839
7.7237
7.7053
7.4415
7.4382
7.4232
7.4199
NH2
Cl
Cl
HO
N
N
N
O
Br
0.01
1.05
1.05
1.10
3.07
f1 (ppm)

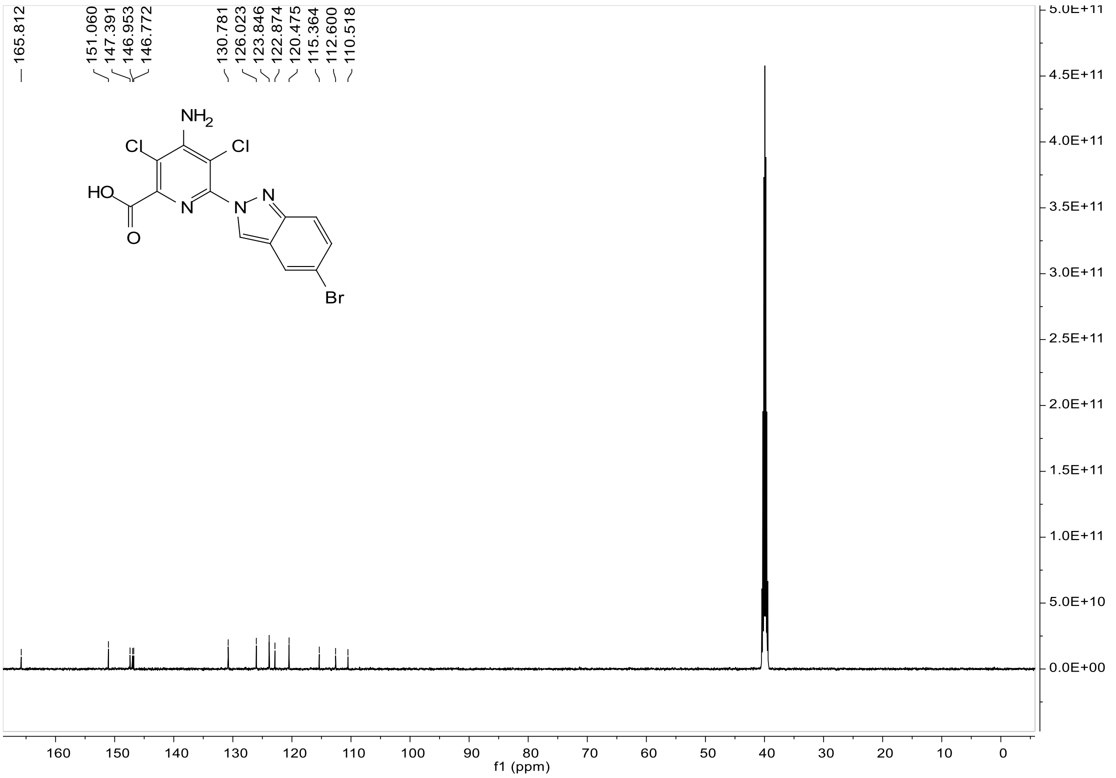
165.812
151.060
147.391
146.953
146.772
130.781
126.023
123.846
122.874
120.475
115.364
112.600
110.518
NH2
Cl
Cl
HO
N
N
N
O
Br
f1 (ppm)

Compound 7Cc

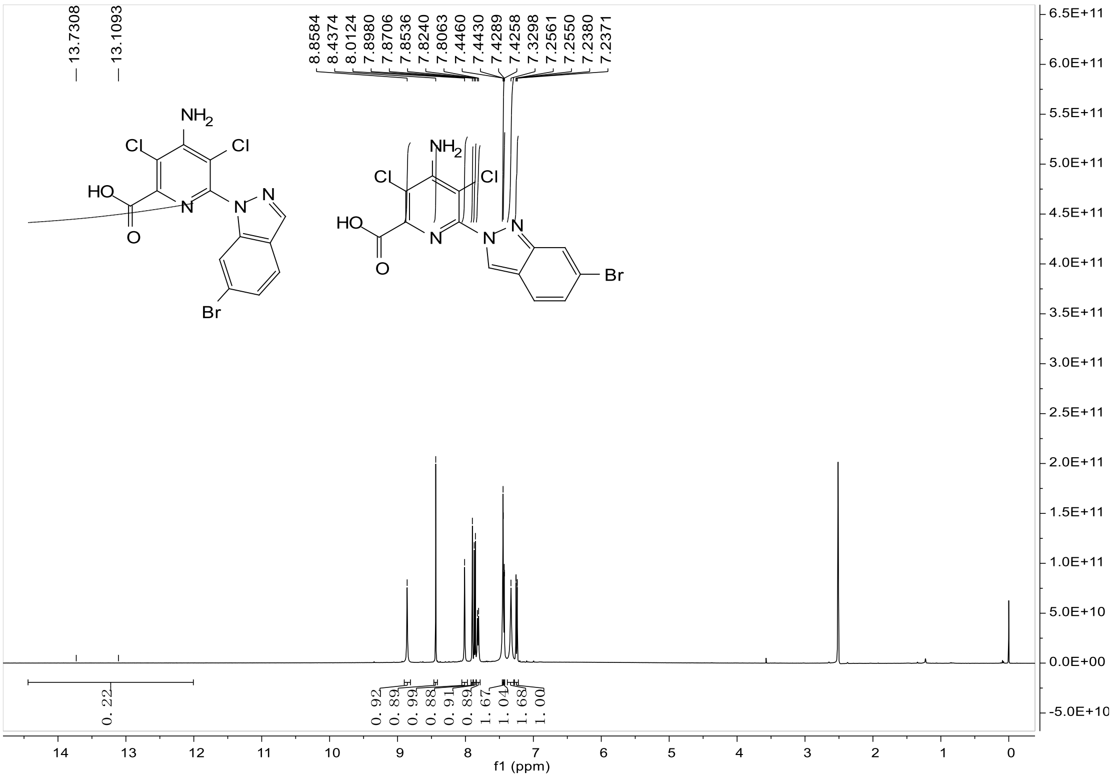

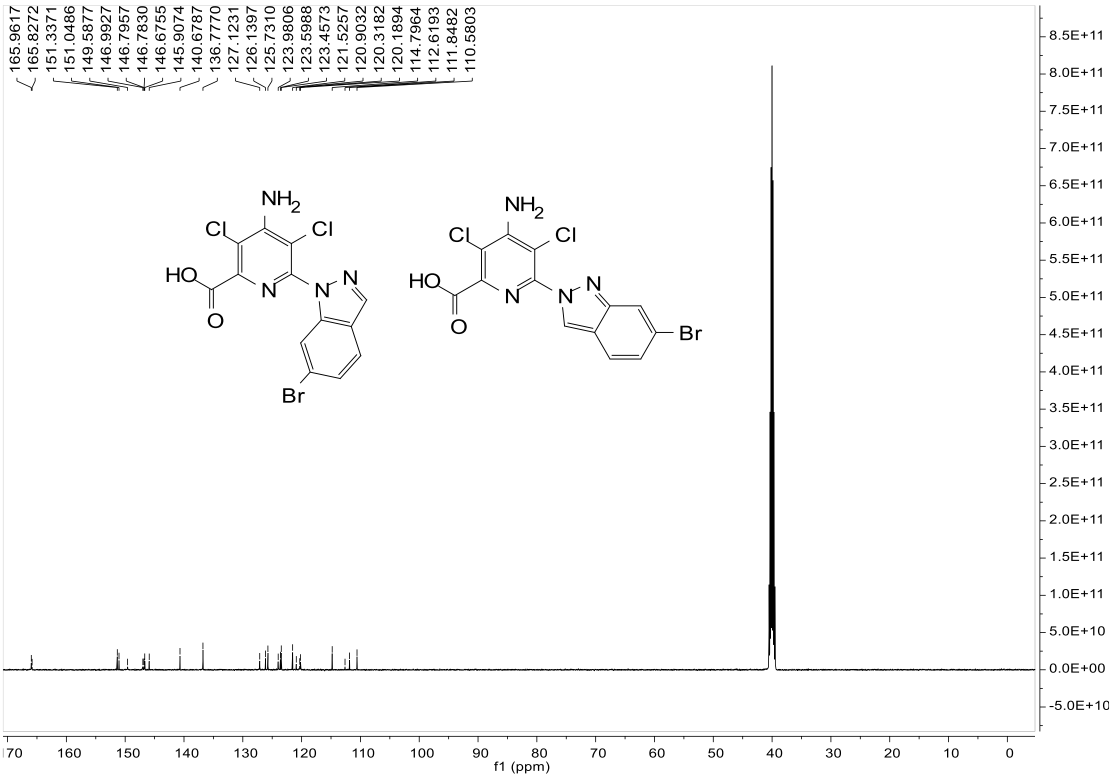

Compound 7d

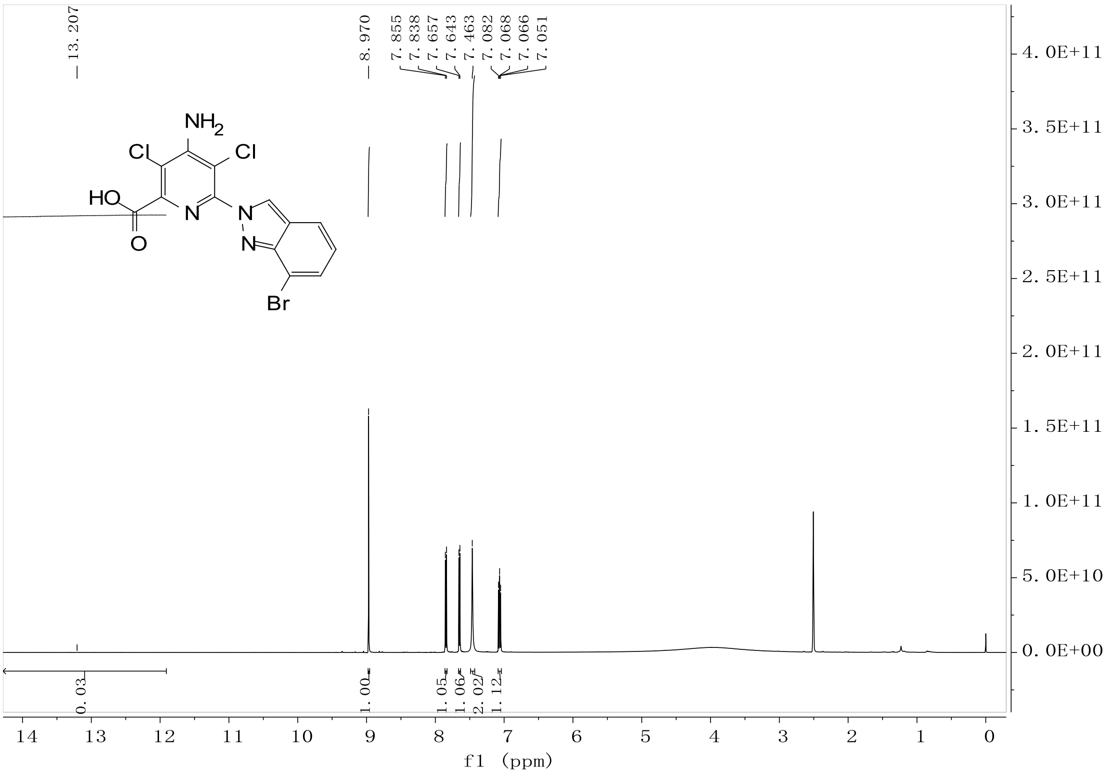

13.207
8.970
7.855
7.838
7.657
7.643
7.463
7.082
7.068
7.066
7.051
NH2
Cl
Cl
HO
N
N
N
O
Br
0.03
1.00
1.05
1.06
2.02
1.12
f1 (ppm)

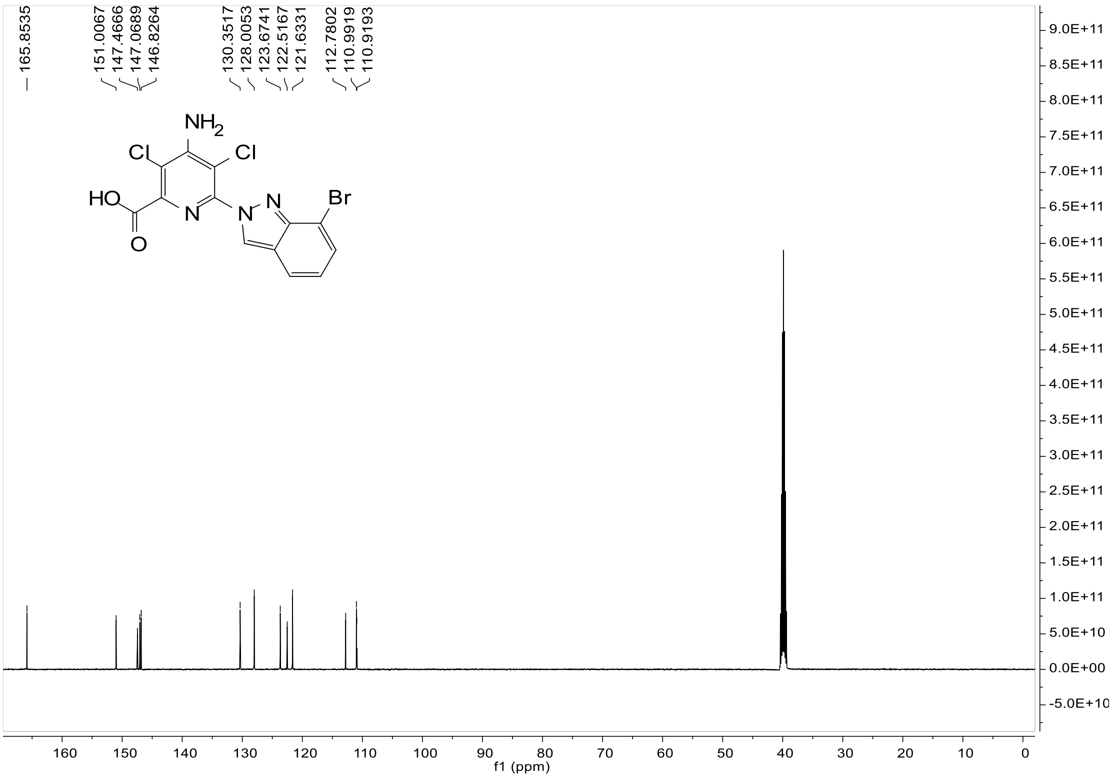

165.8535
151.0067
147.4666
147.0689
146.8264
130.3517
128.0053
123.6741
122.5167
121.6331
112.7802
110.9919
110.9193
NH2
Cl
Cl
HO
N
N
N
Br
O
f1 (ppm)
